# Supplementary material for: Single-Cell Transcriptomic Analysis of Tumor-Derived Fibroblasts and Normal Tissue-Resident Fibroblasts Reveals Fibroblast Heterogeneity in Breast Cancer
Source: Cancers (Basel). 2020 May 21;12(5):1307. doi: 10.3390/cancers12051307 (PMC7281266; doi:10.3390/cancers12051307)
Supplement: Supplementary file 1 [file cancers-12-01307-s001.pdf]

cancers

MDPI

Article

Single-Cell Transcriptomic Analysis of Tumor-Derived Fibroblasts and Normal Tissue-Resident Fibroblasts Reveals Fibroblast Heterogeneity in Breast Cancer

Aimy Sebastian, Nicholas R. Hum, Kelly A. Martin, Sean F. Gilmore, Ivana Peran, Stephen W. Byers, Elizabeth K. Wheeler, Matthew A. Coleman and Gabriela G. Loots

Supplementary Materials

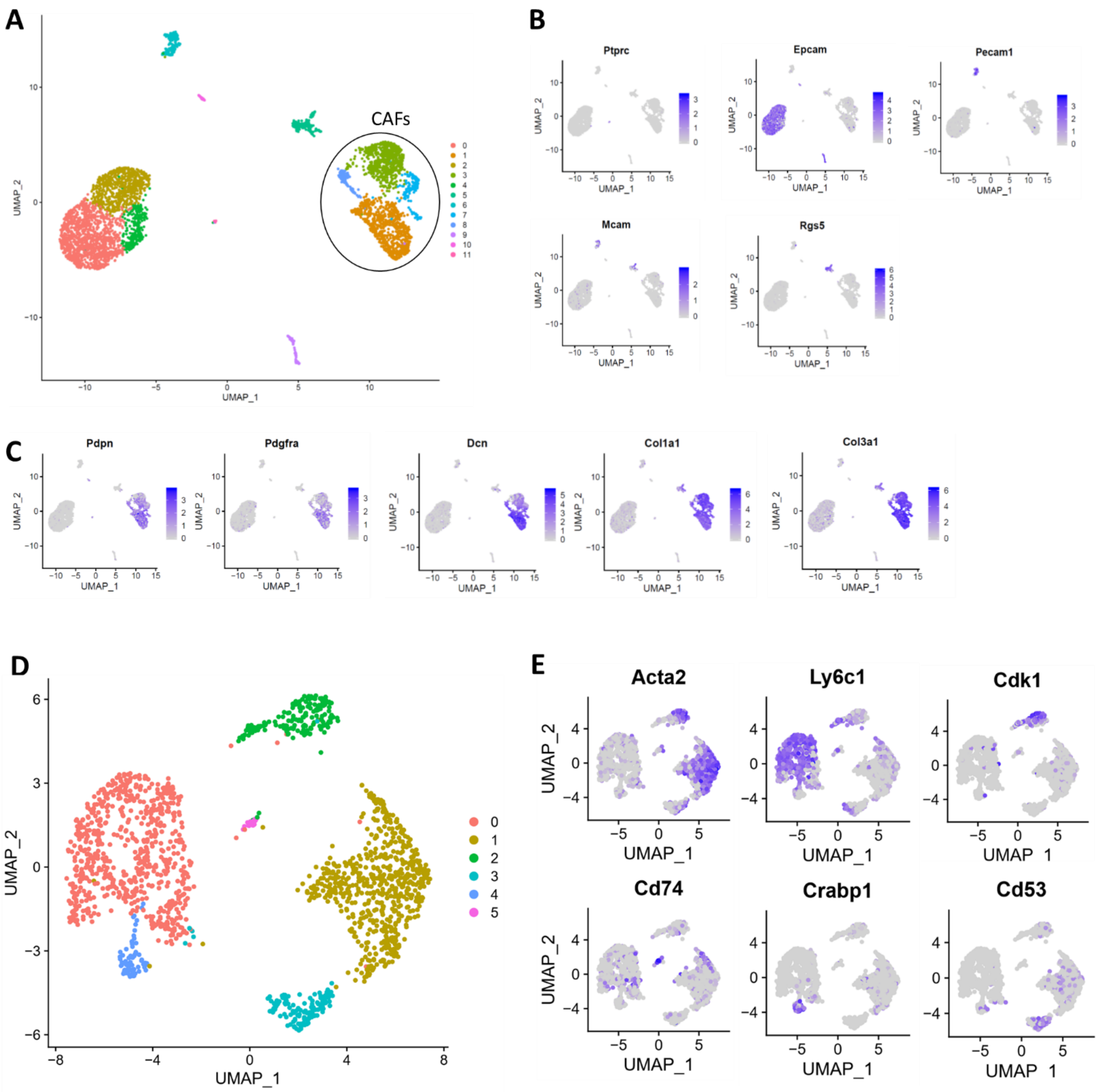

Figure S1. Single-cell sequencing of immune depleted stromal cell fraction. (**A**) UMAP plot showing various cell types identified in 4T1-Thy1.1 tumors after depleting blood cells, immune cells and Thy1.1 expressing cancer cells. (**B**) Feature plots showing the expression of cell type-specific markers Ptprc (immune cells), Epcam (epithelial cells), Pecam1 (endothelial cells), Mcam (endothelial cells and pericytes), Rgs5 (pericytes). (**C**) Feature plots showing the expression of CAF markers. (**D**) Six CAF clusters identified with 1000 most variable genes. (**E**) Feature plots showing the expression of CAF markers *Acta2*, *Ly6c1*, *Cdk1*, *Cd74*, *Crabp1* and *Cd53* in the CAF clusters identified with 1000 most variable genes.

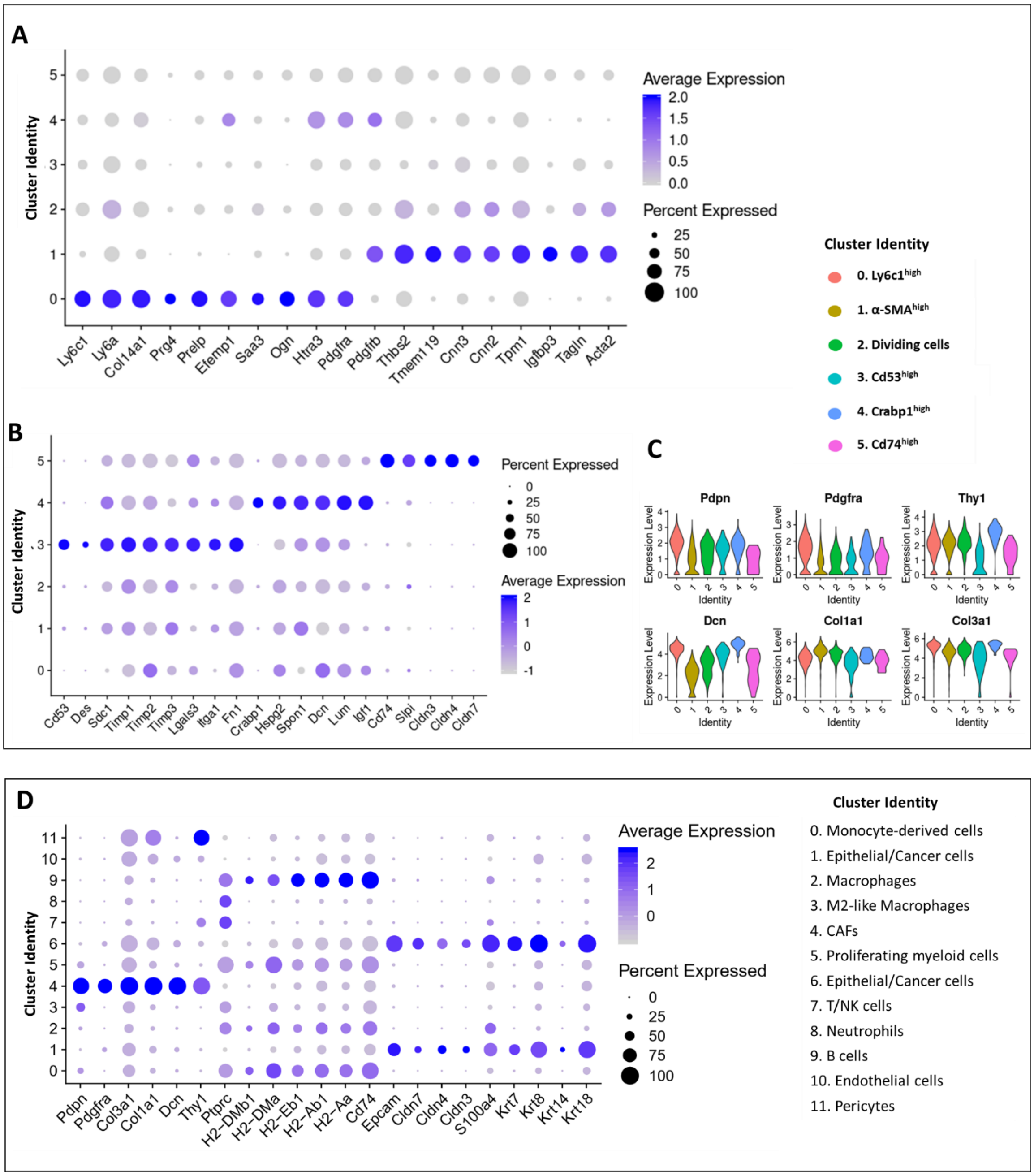

Figure S2. Markers of various CAF subtypes. (**A**) Dot plot showing the expression of selected markers of Ly6c1^{high} (cluster 0) and α-SMA^{high} (cluster 1) CAFs. Dot size represents the fraction of cells expressing a specific marker in a particular cluster and intensity of color indicates the average expression in that cluster. (**B**) Dot plot showing the expression of selected markers of Cd53^{high} (cluster 3), Crabp1^{high} (cluster 4) and Cd74^{high} (cluster 5) CAFs. Dot size represents the fraction of cells expressing a specific marker in a particular cluster and intensity of color indicates the average expression in that cluster. (**C**) Violin plot showing the expression of commonly used CAF markers in various CAF subtypes. (**D**) Dot plot showing the expression of pan-CAF markers and Cd74^{high} CAF markers in immune and epithelial cells from 4T1 tumors.

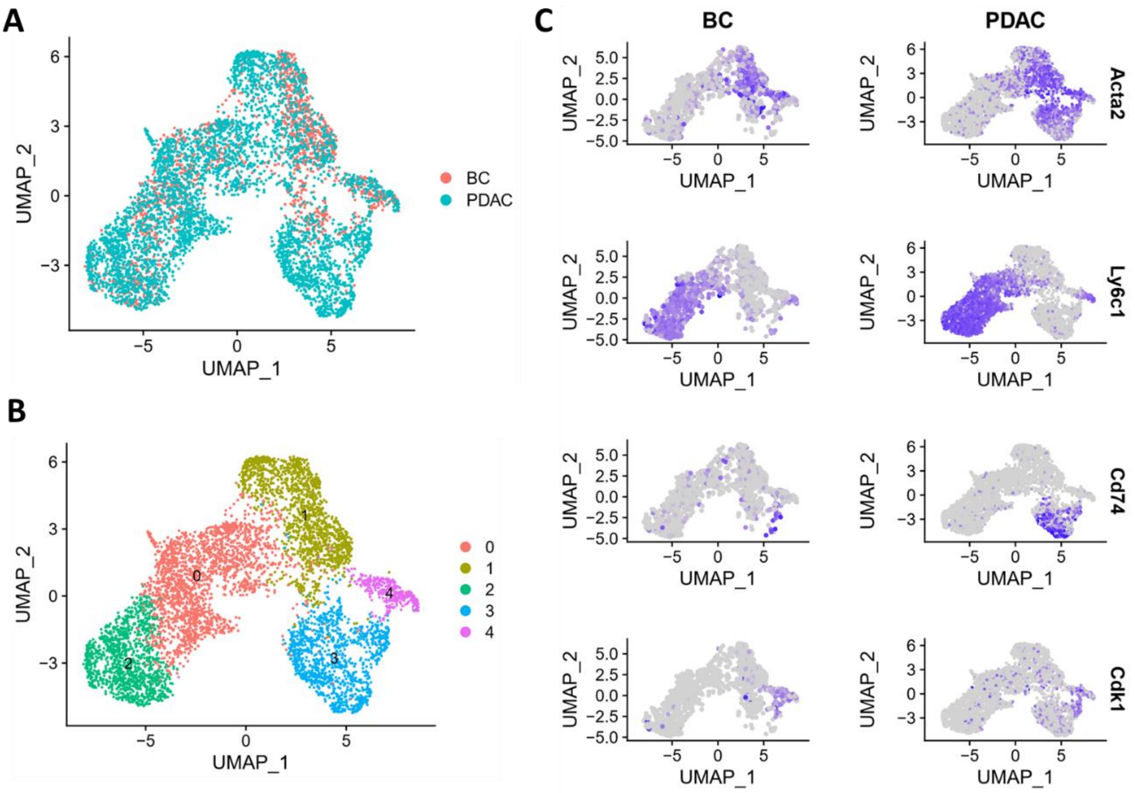

Figure S3. Comparative analysis of 4T1-derived CAFs (BC) and KPC -derived CAFs (PDAC). (**A**) UMAP plot of aligned BC and PDAC CAF subtypes colored based on tumor types. (**B**) UMAP plot of aligned BC and PDAC CAF subtypes colored based on CAF sub-type assignment. (**C**) Feature plot showing the expression of myCAF (*Acta2*), iCAF (*Ly6c1*), apCAF (*Cd74*) markers and cell cycle marker *Cdk1* in BC and PDAC derived CAFs.

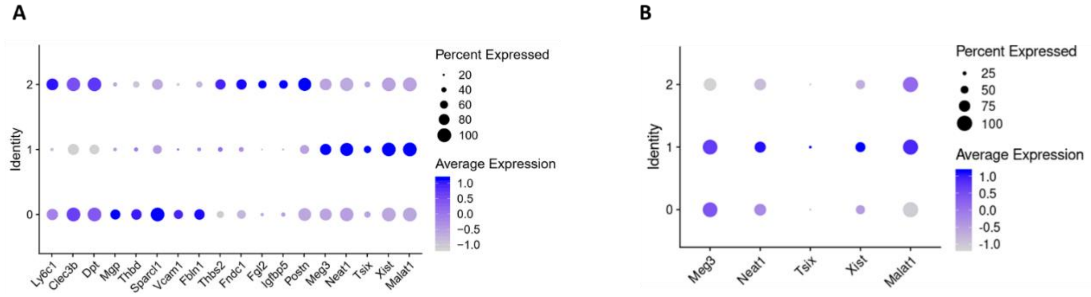

Figure S4. Expression profiles of tissue resident fibroblasts. (**A**) Dot plot showing the expression of genes differentially expressed between normal mammary fat pad-derived fibroblast clusters. Dot size represents the fraction of cells expressing a specific marker in a particular cluster and intensity of color indicates the average expression in that cluster. (**B**) Dot plot showing the expression of long non-coding RNAs in normal pancreas-derived fibroblasts. Dot size represents the fraction of cells expressing a specific marker in a particular cluster and intensity of color indicates the average expression in that cluster.

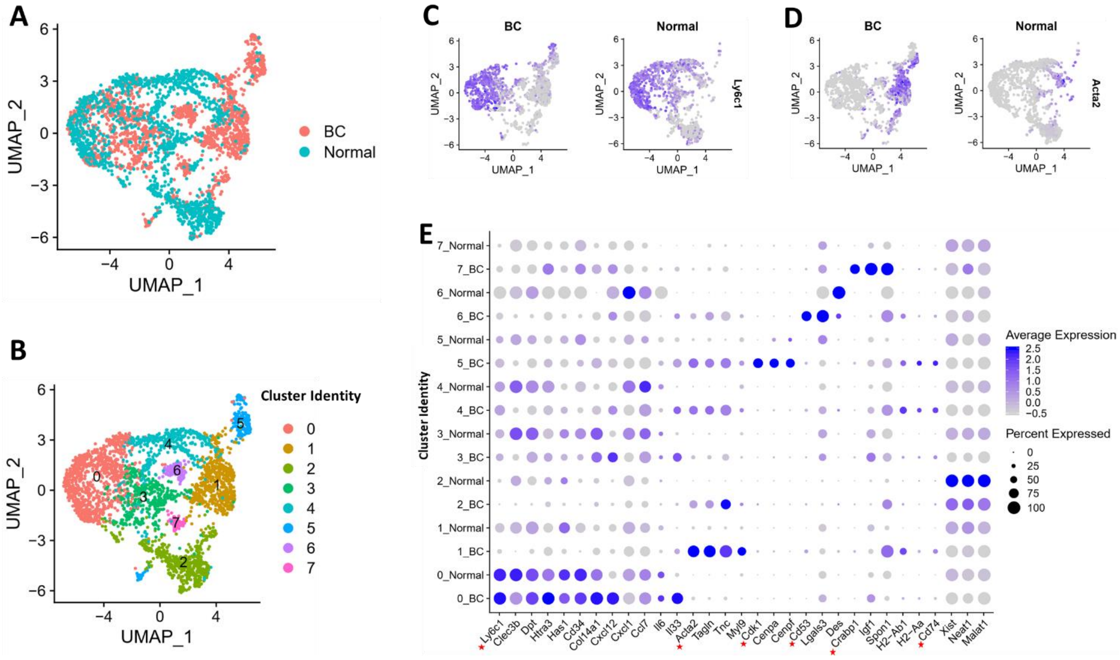

Figure S5. Comparative analysis of breast cancer-derived CAFs and normal mammary fat pad-derived fibroblasts. (**A**) UMAP plot showing aligned tumor-derived CAFs (BC) and normal mammary fat pad- derived fibroblasts (Normal), colored based on tissue type. Ly6c1^{high} CAFs from the tumor aligned with Ly6c1^{high} fibroblasts from normal mammary fat pad (cluster 0). (**B**) UMAP plot of aligned tumor-derived CAFs and normal mammary fat pad-derived fibroblasts colored based on CAF sub-type assignment. (**C**,**D**) Feature plot showing the expression of iCAF marker *Ly6c1* (**C**) and myCAF marker *Acta2* (**D**) in aligned tumor-derived CAFs and normal mammary fat pad-derived fibroblasts. (**E**) Dot plot showing the expression of selected CAF subtype markers in tumor-derived CAFs and normal mammary fat pad- derived fibroblast clusters. Ly6c1^{high} CAFs (cluster 0) from the tumor showed high similarity with Ly6c1^{high} fibroblasts (cluster 0) from normal mammary fat pad. Dot size represents the fraction of cells expressing a specific marker in a particular cluster and intensity of color indicates the average expression in that cluster. Representative markers of the six CAF clusters are highlighted with red stars.

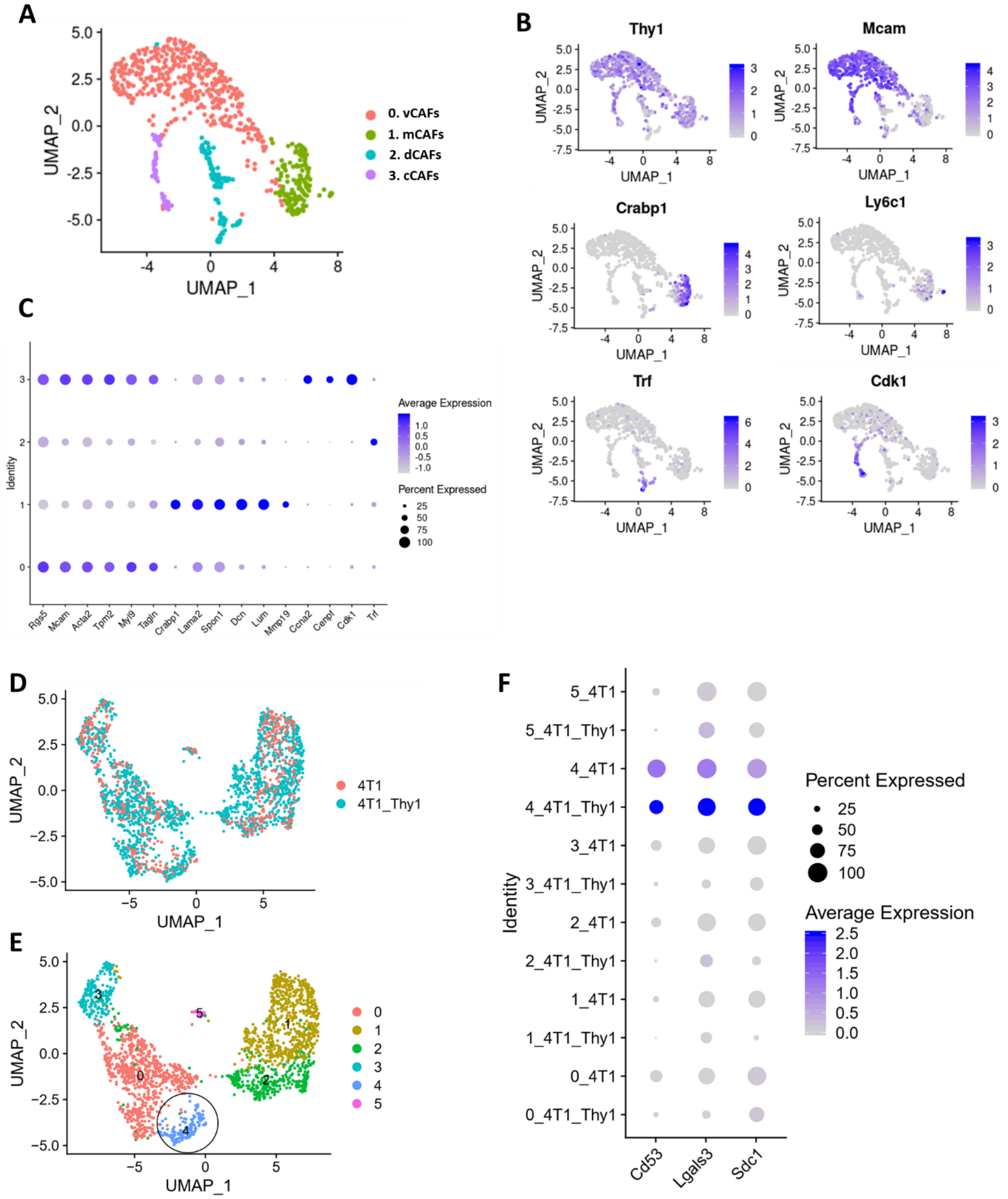

Figure S6. Identification Crabp1^{high} and Cd53^{high} CAFs in multiple breast cancer datasets. (**A**) UMAP plot showing various CAF subtypes in MMTV-PyMT mouse model of breast cancer. (**B**) Feature plots showing the expression of CAF marker Thy1 and selected subtype-specific markers in MMTV-PyMT mouse model of breast cancer. (**C**) Dot plot showing the expression of selected subtype markers (cluster 0: vCAFs; cluster 1: mCAFs; cluster 2: dCAFs; cluster 3 : cCAFs) in MMTV-PyMT mouse model of breast cancer. Dot size represents the fraction of cells expressing a specific marker in a particular cluster and intensity of color indicates the average expression in that cluster. (**D**) UMAP plot showing the alignment of 4T1-derived (4T1) and 4T1-Thy1.1 derived (4T1_Thy1) CAFs. (**E**) UMAP plot of aligned 4T1-derived and 4T1-Thy1.1 derived CAFs colored based on CAF sub-type assignment. Cd53^{high} CAF cluster (cluster 4) is highlighted. (**F**) Dot plot showing the expression of selected markers of Cd53^{high} CAFs in 4T1-derived and 4T1-Thy1.1 CAFs. Dot size represents the fraction of cells expressing a specific marker in a particular cluster and intensity of color indicates the average expression in that cluster.

Table S1. Genes enriched (fold change >1.25) in each 4T1 tumor-derived cluster compared to all other clusters.

Cluster ID	***Marker Genes***	***Fold Enrichment (log2)***	***p_val_adj***
0	Apoe	1.1854	0
0	Cd74	1.1450	1.72E-259
0	C1qb	1.1208	1.48E-287
0	C1qa	1.1146	9.41E-293
0	Trf	1.1110	7.43E-275
0	H2-Eb1	1.1074	6.05E-121
0	H2-Aa	1.0839	1.93E-143
0	C1qc	1.0692	2.78E-286
0	H2-Ab1	1.0588	1.61E-142
0	Ctss	1.0494	0
0	Ctsc	1.0056	0
0	Lyz2	0.9809	9.15E-303
0	Gatm	0.9723	0
0	Aif1	0.9365	6.53E-296
0	Bcl2a1b	0.8923	0
0	H2-DMa	0.8804	2.25E-278
0	Cxcl16	0.8627	1.75E-283
0	Arg1	0.8551	3.49E-250
0	Ly86	0.8375	5.40E-291
0	Ccl12	0.8176	7.83E-130
0	Ccl24	0.7850	5.22E-165
0	Clec4n	0.7722	9.44E-228
0	Lgmn	0.7475	1.40E-271
0	Ms4a6c	0.7427	1.27E-244
0	Ctsz	0.7354	2.43E-295
0	Ifi30	0.7277	1.11E-225
0	Psap	0.7210	1.10E-272
0	H2-Ea-ps	0.7120	7.03E-114
0	Cybb	0.7042	3.30E-226
0	Msr1	0.7033	1.63E-233
0	Fcgr3	0.6993	1.09E-247
0	Bcl2a1a	0.6955	1.19E-233
0	Csf1r	0.6922	5.22E-247
0	Cd83	0.6887	9.57E-153
0	Trem2	0.6871	1.88E-241
0	Rgs1	0.6636	5.90E-161
0	Bcl2a1d	0.6412	2.41E-219
0	Fcgr2b	0.6367	1.22E-224
0	Pf4	0.6347	1.26E-76
0	Mpeg1	0.6344	7.68E-204
0	Fcer1g	0.6304	5.71E-211
0	H2-DMb1	0.6230	6.47E-100
0	Cotl1	0.6113	1.12E-197
0	Cfp	0.6109	1.21E-182
0	Ctsa	0.6106	1.25E-209
0	Ctsb	0.6097	1.60E-222
0	Cd300d	0.6093	4.24E-200
0	Grn	0.6075	5.09E-228
0	Cyba	0.6072	1.93E-240
0	Tyrobp	0.6023	6.29E-211
0	Rgs10	0.5995	1.19E-182
0	Pld4	0.5967	3.99E-180
0	Tgfbi	0.5934	1.52E-165
0	Clec7a	0.5926	8.68E-135
0	Gm2a	0.5903	1.54E-179
0	Unc93b1	0.5885	8.14E-202
0	Sdc3	0.5882	2.06E-162
0	Il1b	0.583113095	1.44E-159

0	Fcgr4	0.581229022	2.06E-165
0	Hexb	0.580629381	1.44E-125
0	Dab2	0.580503883	4.20E-219
0	Lst1	0.579967496	1.26E-161
0	Acp5	0.576557046	1.09E-98
0	Mafb	0.569372897	3.30E-178
0	Ms4a6d	0.566825136	4.62E-179
0	Il1rn	0.561086629	1.84E-125
0	Mgl2	0.556826323	3.62E-94
0	Ccl6	0.55038164	1.38E-167
0	Ms4a6b	0.549184423	2.18E-166
0	Clec4a2	0.548583239	1.08E-162
0	C3ar1	0.546918267	3.90E-182
0	Cdkn1a	0.541874795	1.68E-133
0	Hexa	0.541629641	2.11E-167
0	Ms4a7	0.539769826	1.79E-97
0	Hebp1	0.535431525	2.25E-155
0	Efhd2	0.531586152	4.10E-164
0	Cd38	0.531084642	1.57E-149
0	Laptm5	0.523975896	3.14E-186
0	Ucp2	0.522521105	4.70E-162
0	Lair1	0.518422067	1.36E-169
0	Ccr5	0.511522623	1.16E-158
0	Fcgr1	0.508858778	9.41E-138
0	Ptgs1	0.506688523	7.54E-120
0	Mmp12	0.506314823	1.56E-21
0	Ccrl2	0.502975167	3.02E-122
0	Rgs2	0.49632989	1.77E-108
0	Il1a	0.490898815	1.36E-95
0	Gngt2	0.485336156	4.28E-140
0	Osm	0.48391042	6.29E-105
0	RP23-52N2.1	0.474167524	2.51E-42
0	Mmp13	0.472072873	4.00E-24
0	Clec4a3	0.469147763	1.45E-127
0	Ccl9	0.464869985	4.34E-137
0	Adgre1	0.464517194	3.17E-122
0	Pla2g7	0.464314726	1.20E-98
0	Cd14	0.462090579	2.00E-151
0	Cndp2	0.457391808	2.76E-130
0	Snx5	0.457308802	5.49E-125
0	Ntpcr	0.455425169	1.93E-91
0	Clta	0.452365717	7.68E-164
0	Ftl1	0.451590363	1.81E-197
0	Irf7	0.451032119	2.81E-35
0	Tbxas1	0.450238167	1.26E-126
0	Ncf4	0.449738581	3.33E-138
0	Mrc1	0.447419504	3.96E-195
0	Lat2	0.445300887	2.09E-123
0	Ms4a4c	0.442510205	4.72E-59
0	Creg1	0.439998307	2.90E-109
0	Cd68	0.438863045	1.69E-130
0	Tmsb4x	0.435248392	6.40E-157
0	Isg15	0.431919693	0.047273
0	Spi1	0.430478281	4.17E-138
0	Klf2	0.429600807	2.11E-47
0	Ctsh	0.419471567	2.29E-104
0	Ccr2	0.416236462	2.07E-85
0	Irf8	0.415326762	1.27E-122
0	Erp29	0.4148114	2.18E-104
0	Lamp1	0.413672642	3.21E-146
0	Pycard	0.411808176	5.02E-111

0	Plek	0.410416169	1.20E-142
0	Sdcbp	0.408664354	1.10E-137
0	Arrb2	0.407030308	3.33E-112
0	Arhgdib	0.406979173	1.79E-113
0	Sirpa	0.406589517	3.31E-107
0	Amica1	0.406519702	9.31E-117
0	Cd72	0.406049873	8.03E-89
0	Kctd12	0.405554388	1.06E-93
0	Slc15a3	0.404357917	8.72E-99
0	Ctsd	0.403963838	5.73E-107
0	Vsir	0.403743644	6.30E-115
0	Smdt1	0.403692581	7.76E-109
0	Fermt3	0.40290045	4.57E-126
0	Fam105a	0.401093874	5.91E-108
0	Lyn	0.400971202	1.26E-107
0	Fcrls	0.399803486	1.25E-70
0	Fabp5	0.399223603	1.01E-10
0	Gusb	0.398949923	2.51E-78
0	Man2b1	0.39872098	2.88E-88
0	Atp6v0b	0.397958548	2.01E-109
0	Cd84	0.390645097	9.07E-115
0	Csf2rb	0.389173223	3.54E-115
0	Tpp1	0.388529115	1.54E-79
0	Cd52	0.38498766	8.34E-124
0	RP24-176A1.3	0.383341051	1.50E-123
0	Pilra	0.383297283	4.36E-121
0	Fcgrt	0.382956452	2.18E-101
0	Mampt	0.382212082	2.83E-72
0	Ncf2	0.382184506	1.01E-136
0	Apobec1	0.377779383	1.03E-91
0	Ptpn18	0.376323573	6.76E-108
0	Sdf2l1	0.374696338	9.57E-65
0	Sat1	0.370954944	6.01E-103
0	Cltc	0.370872204	9.55E-67
0	Plekho1	0.368367436	1.68E-100
0	Ifrd1	0.368007887	4.62E-60
0	Runx3	0.364491932	5.25E-111
0	Nr4a3	0.364341434	6.27E-59
0	Gdi2	0.364187239	6.10E-84
0	Nlrp3	0.364006385	1.03E-73
0	Ifi27l2a	0.363950708	3.68E-80
0	Naaa	0.363890521	1.49E-78
0	Prdx5	0.362633844	2.30E-102
0	Abca1	0.362566497	5.75E-71
0	Rnf130	0.359815386	2.64E-68
0	Snx2	0.35747669	1.21E-66
0	Pkib	0.356055084	1.43E-108
0	H2-DMb2	0.355866983	6.15E-95
0	Lgals3bp	0.352520817	1.21E-41
0	Sla	0.350986046	4.30E-96
0	F7	0.349672468	4.08E-76
0	Nfe2l2	0.348986991	5.04E-54
0	Prkcd	0.348915364	1.77E-64
0	Atp1b3	0.346819196	2.49E-61
0	Fam96a	0.345191962	2.13E-63
0	Dok2	0.342991408	5.38E-80
0	Ptpn6	0.341951296	3.92E-83
0	Slc25a5	0.340804308	1.70E-72
0	Ifi44	0.336255299	9.49E-27
0	Itm2b	0.33542623	8.71E-79
0	Hcls1	0.334438421	8.79E-100

0	Renbp	0.332736979	8.46E-78
0	Cox5a	0.33254687	8.42E-80
0	Acly	0.331582096	2.80E-40
0	Ubl3	0.32980012	2.17E-61
0	RP24-261H7.9	0.329400201	3.70E-58
0	Zeb2	0.328597014	1.12E-72
0	Coro1a	0.328005011	5.77E-100
0	Ppfia4	0.327467958	1.14E-97
0	Tmem160	0.32715857	1.36E-65
0	Ets2	0.326484813	1.84E-73
0	Tlr2	0.326142913	3.49E-64
0	Fth1.1	0.325421152	6.36E-147
0	Ncf1	0.325333746	6.88E-89
0	Scimp	0.325221926	6.45E-85
0	Akr1a1	0.324384518	1.57E-78
0	Tmem189	0.323505155	3.89E-62
1	Wfdc2	1.9047	0
1	Krt18	1.8390	0
1	Lars2	1.5874	1.71E-135
1	Krt8	1.5272	0
1	Padi4	1.4254	0
1	RP24-127H11.10	1.4113	0
1	Clu	1.4024	0
1	Mgp	1.2881	0
1	Spint2	1.2529	0
1	Tm4sf1	1.2523	0
1	Cp	1.2438	0
1	Id3	1.2378	2.79E-200
1	S100a6	1.2111	0
1	Nupr1	1.2087	3.49E-257
1	Epcam	1.2076	0
1	H19	1.1884	4.60E-136
1	RP23-81C12.1	1.1717	1.92E-77
1	RP23-81C12.3	1.1478	1.90E-92
1	Sox4	1.1474	0
1	Hmga2	1.0611	0
1	Ccnd1	1.0502	8.18E-268
1	Slco2a1	1.0492	0
1	AC164564.3	1.0360	0
1	Nfib	1.0344	0
1	Phlda1	0.9901	5.56E-146
1	Fxyd3	0.9830	7.35E-214
1	Tslp	0.9567	0
1	Sesn3	0.9557	0
1	Rhoc	0.9535	3.98E-299
1	Gas5	0.9454	6.59E-271
1	Zfas1	0.9417	4.62E-209
1	Gm26825	0.9388	3.31E-119
1	Mtap	0.9212	7.58E-287
1	RP23-310J6.1	0.9130	6.82E-302
1	Maged1	0.9037	1.65E-257
1	Cd9	0.8903	6.69E-233
1	Rn7sk	0.8869	3.87E-70
1	Ank	0.8826	0
1	Spata7	0.8717	5.75E-187
1	Lgals1	0.8682	0
1	Mt2	0.8679	2.91E-63
1	Moxd1	0.8594	0
1	Amotl1	0.8403	0
1	Tnnt2	0.8194	0
1	Tbrg1	0.8186	1.16E-242

1	Tnfrsf12a	0.8139	1.20E-223
1	Mast4	0.8106	0
1	Prkg2	0.7988	0
1	Gadd45a	0.7978	1.10E-191
1	Krt7	0.7909	0
1	Cdkn2c	0.7882	1.10E-286
1	Prdx2	0.7881	7.33E-213
1	Gm47283	0.7796	1.86E-179
1	Map1b	0.7784	0
1	Urah	0.7726	0
1	RP23-8J15.7	0.7688	6.13E-105
1	Cald1	0.7686	6.77E-283
1	Sox9	0.7676	2.02E-291
1	Rps26	0.7482	0
1	Npm1	0.7472	1.35E-258
1	Lmna	0.7444	2.61E-166
1	Cpe	0.7418	0
1	Ltbp3	0.7376	4.44E-279
1	Slpi	0.7273	6.16E-96
1	Abhd2	0.7270	1.17E-162
1	Igfbp4	0.7265	1.86E-244
1	Itga3	0.7227	0
1	RP23-442M18.1	0.7156	0
1	RP23-234K24.8	0.7153	1.80E-135
1	Pdgfrl	0.7125	1.91E-295
1	Usp50	0.7109	3.01E-150
1	Ndufb9	0.6945	5.00E-193
1	Cldn4	0.6786	1.44E-269
1	Sdc2	0.6785	7.92E-243
1	Tmem176b	0.6724	3.56E-152
1	Rpl36	0.6699	2.46E-302
1	Taf1d	0.6661	1.35E-143
1	Gpx4	0.6658	3.62E-216
1	Pebp1	0.6580	7.92E-165
1	Bc1	0.6565	2.65E-109
1	Serpine2	0.6532	6.05E-140
1	Krtcap3	0.6477	0
1	Rpl37	0.6467	0
1	Lcn2	0.6457	1.26E-211
1	Eif3e	0.6424	1.19E-140
1	Trps1	0.6402	5.16E-149
1	Ptprk	0.6401	0
1	Naca	0.6380	4.34E-258
1	Vcam1	0.6359	6.54E-101
1	S100a4	0.6322	4.34E-108
1	RP23-8J15.6	0.6282	2.61E-79
1	Wnt7b	0.6229	0
1	Npnt	0.6215	8.66E-279
1	Rpl30	0.6152	2.48E-241
1	Cttn	0.6066	1.66E-200
1	Rpl26	0.6045	1.63E-241
1	Etv1	0.5972	1.37E-226
1	Snhg8	0.5969	6.16E-122
1	AC026478.1	0.5967	7.36E-252
1	Nfix	0.5943	5.09E-201
1	S100a10	0.5922	1.23E-158
1	Rps20	0.5916	2.32E-287
1	Itgb4	0.5906	0
1	Ercc1	0.5903	2.48E-193
1	Perp	0.5893	2.27E-299
1	Cd24a	0.5876	4.42E-191

1	H2-Q4	0.5872	7.39E-172
1	Rpl18a	0.583094889	0
1	Fut8	0.582650436	3.98E-193
1	Prss22	0.582533104	2.93E-250
1	Vim	0.579222632	7.96E-100
1	Ifi27	0.578483037	7.90E-120
1	Hmgn1	0.577072942	2.81E-132
1	Lama5	0.577034464	1.90E-300
1	Nedd4	0.576353243	1.78E-194
1	Rpl23	0.575973954	0
1	Anxa5	0.574741926	1.18E-119
1	Rps12	0.573017882	3.73E-248
1	Cystm1	0.571698253	4.94E-181
1	Car9	0.569939432	3.84E-212
1	Pafah1b3	0.567946691	7.04E-174
1	Rbp1	0.56598338	1.46E-168
1	Ddah2	0.564454687	1.03E-204
1	Nenf	0.563301372	3.02E-139
1	Ltbp1	0.560329356	1.16E-272
1	Slc25a4	0.556783331	6.25E-109
1	RP23-8J15.5	0.555458715	4.04E-55
1	Cyr61	0.553509089	9.56E-87
1	Jun	0.55240799	7.84E-46
1	Casp3	0.54916023	1.70E-168
1	Slc29a1	0.547040796	1.37E-132
1	Pbx1	0.545453462	9.07E-187
1	Cdk4	0.544917546	1.50E-115
1	Gata3	0.54203913	0
1	Csf3	0.537649087	2.80E-148
1	H1f0	0.536270477	1.31E-115
1	Baiap2	0.534612666	1.93E-134
1	Bin1	0.53305611	1.51E-130
1	Hoxa5	0.532166532	4.38E-299
1	RP23-8J15.3	0.53145392	3.55E-72
1	Dusp6	0.530895825	2.44E-110
1	Map1lc3a	0.530870347	3.79E-112
1	RP23-349B4.7	0.530708291	1.51E-128
1	Cnn3	0.527222483	1.32E-180
1	H3f3b	0.527109755	1.05E-190
1	Dst	0.526547266	7.00E-193
1	RP23-161B9.10	0.526127455	2.83E-112
1	Cited2	0.523565875	4.44E-76
1	Errfi1	0.523488605	1.42E-114
1	Uba52	0.522641275	4.94E-230
1	Rpl41	0.521944689	0
1	Rpl6	0.521226317	8.60E-217
1	Rps18	0.518929144	2.21E-242
1	Hotairm1	0.515543357	2.77E-168
1	Rps15	0.514449474	3.00E-254
1	Gja1	0.513960582	1.33E-146
1	Eif4a2	0.511817127	7.65E-99
1	Polr2f	0.509290991	1.09E-111
1	Eny2	0.508462137	2.71E-110
1	Rpl31	0.507701242	7.37E-202
1	Col18a1	0.506983599	6.04E-216
1	Spp1	0.506408719	6.65E-81
1	Fkbp3	0.504212198	1.31E-111
1	Ptprs	0.503411042	3.77E-157
1	Tmsb10	0.501106871	3.44E-144
1	Hnrnph1	0.499479661	4.21E-106
1	Rplp0	0.498072771	4.03E-180

1	Oaf	0.493941726	4.34E-139
1	Rpl28	0.490813278	4.61E-213
1	Gng11	0.490484105	8.05E-166
1	Plet1	0.488272262	8.92E-233
1	Dut	0.487182354	7.59E-119
1	Cd151	0.486572251	2.23E-168
1	Lamb1	0.486098774	1.14E-210
1	Rpl23a	0.48562527	4.11E-213
1	Rpl38	0.485454071	6.66E-224
1	Sqstm1	0.485310574	9.62E-84
1	Runx1	0.485251039	1.30E-83
1	RP23-336M12.2	0.484369106	1.64E-140
1	Tinagl1	0.481821399	6.36E-221
1	Hsp90ab1	0.480444863	2.27E-106
1	Cnpy2	0.479502213	1.90E-96
1	Ttc3	0.479333315	5.45E-172
1	Cfl2	0.479095843	6.14E-141
1	Rpl12	0.478802999	2.48E-169
1	Nudt4	0.475939543	5.61E-113
1	Asap1	0.474964957	2.09E-137
1	Nudt9	0.473352393	4.16E-113
1	RP23-79J21.3	0.465979834	1.67E-177
1	Tanc2	0.465900607	2.83E-151
1	Mif	0.465666508	2.96E-68
1	Ace	0.465581956	9.77E-188
1	Phip	0.464886595	1.96E-114
1	Pkd2	0.461640689	5.11E-140
1	Tsc22d1	0.460724073	6.67E-111
1	Epn2	0.458651927	5.77E-243
1	Rab34	0.45810966	3.34E-184
1	Rps10	0.457653388	1.00E-181
1	Eef1d	0.454913063	2.95E-101
1	Snhg1	0.453441608	1.84E-97
1	Wls	0.451932576	3.45E-87
1	Rasa2	0.451188239	2.01E-153
1	Bmp1	0.450444984	8.68E-159
1	Tmem176a	0.450314055	5.92E-109
1	Cldn3	0.449998493	6.96E-226
1	Krt19	0.44793104	2.92E-161
1	Tead1	0.447790121	3.07E-180
1	Rpl22	0.446445897	1.63E-163
1	Tspan4	0.445756075	2.45E-90
1	Ndufa4	0.445746493	1.68E-84
1	Kmt2e	0.443581871	1.20E-104
1	Cct5	0.441941311	1.67E-79
1	Tra2a	0.441734747	5.53E-92
1	Fhl2	0.441364665	5.50E-178
1	Tpm1	0.441031827	1.03E-134
1	Impdh2	0.440691641	4.58E-83
1	Rpl14	0.439700321	6.64E-199
1	Ltbp2	0.438558722	9.87E-241
1	RP23-251I6.1	0.437550465	2.90E-246
1	Hspb1	0.437339457	1.00E-52
1	Ahnak	0.437330368	9.45E-87
1	Insl6	0.437073897	4.74E-208
1	H2afv	0.43606188	5.23E-99
1	Tcf4	0.436032697	1.88E-87
1	Tns4	0.435018152	1.35E-195
1	Syt8	0.434951378	9.05E-216
1	Rpl5	0.432622048	1.19E-109
1	Actn1	0.432455434	1.40E-139

1	Rpl39	0.431834441	6.04E-198
1	Arhgap22	0.431721337	3.80E-164
1	Pabpc1	0.431451239	2.97E-81
1	Nfat5	0.431296201	4.78E-123
1	Hspa8	0.430401896	4.50E-37
1	Ndrg1	0.428972037	2.34E-28
1	Tubb5	0.428322272	1.13E-86
1	Klf5	0.428006824	6.31E-196
1	Efna5	0.427879117	2.97E-259
1	Wwtr1	0.427579998	2.34E-195
1	Foxc1	0.426976422	8.81E-250
1	Rps17	0.426322712	2.89E-158
1	Rpl15	0.42533544	1.71E-127
1	Fubp1	0.424294376	8.00E-85
1	Lsm7	0.423475663	3.83E-69
1	Tsen34	0.423419513	2.42E-106
1	Rpl37rt	0.422566343	1.57E-81
1	RP23-472H13.5	0.421595903	1.14E-201
1	Hcfc1r1	0.420255849	1.59E-68
1	AC152164.1	0.418854533	6.41E-113
1	Med24	0.417711718	4.31E-141
1	D030056L22Rik	0.416487096	4.63E-120
1	Mfge8	0.416064104	5.28E-126
1	Aplp2	0.415153779	7.79E-82
1	Rpl13	0.41498494	8.72E-185
1	Lad1	0.414596352	1.97E-229
1	Bok	0.412690537	7.77E-173
1	Prmt1	0.410414635	2.97E-82
1	Ndufa12	0.409658923	1.80E-72
1	Snrpf	0.408985053	7.62E-77
1	Col7a1	0.408219345	4.69E-178
1	Nav2	0.407466187	6.39E-222
1	Uqcc2	0.407190828	2.16E-62
1	Plp2	0.407083105	5.83E-104
1	Gnas	0.406948998	2.61E-78
1	Cox6c	0.406226956	2.96E-112
1	Rps28	0.405850249	8.84E-187
1	Rcn2	0.405045803	4.23E-88
1	Eef2	0.404067423	2.16E-113
1	Glo1	0.403918732	3.58E-87
1	Rnf11	0.403056089	1.07E-85
1	Nap1l1	0.402572778	3.78E-74
1	Rbp2	0.402120075	5.33E-218
1	Rps2	0.402081688	2.32E-106
1	Eno3	0.40166862	2.67E-168
1	Irx2	0.401476249	9.25E-205
1	Tead2	0.401002378	4.64E-235
1	Adgrl2	0.399221675	3.75E-198
1	Nfia	0.399059504	4.91E-127
1	Sfn	0.398841301	6.15E-122
1	Tmem238	0.39881807	3.83E-124
1	RP23-4P23.3	0.398122965	4.99E-87
1	Hmga1-rs1	0.397871691	1.53E-159
1	Rps19	0.397466752	3.63E-183
1	Rpl11	0.397379334	1.76E-177
1	Prkcdbp	0.397260488	2.13E-157
1	Rps7	0.396719579	1.30E-134
1	Acsl4	0.39655792	1.14E-82
1	Asap2	0.396341956	1.86E-244
1	Rpl19	0.396032589	1.14E-177
1	Myc	0.395849258	4.19E-93

1	Ubb	0.395568285	2.67E-66
1	Las1l	0.39531864	1.44E-138
1	AC102483.1	0.395209648	9.33E-85
1	Apbb2	0.393876787	2.34E-174
1	Skp1a	0.393226676	3.47E-78
1	Fam3c	0.392182507	1.15E-99
1	Serinc2	0.392041537	7.73E-255
1	Mrpl38	0.391960873	3.87E-121
1	Ptov1	0.391150147	9.01E-89
1	Fah	0.390839214	3.00E-185
1	Snrpd2	0.389797576	1.04E-70
1	Rpl27	0.388907282	9.48E-151
1	Phgdh	0.388413938	4.68E-162
1	Ppia	0.388235753	6.08E-109
1	Tpbg	0.388138489	1.80E-159
1	Card10	0.387689571	1.23E-207
1	Ankrd1	0.387306919	8.78E-127
1	Emp2	0.387019629	2.09E-204
1	Mtss1	0.385816655	6.79E-60
1	Rpl24	0.38366076	6.21E-121
1	Phpt1	0.38313701	6.92E-111
1	Fam102b	0.383022105	1.28E-97
1	Igf2r	0.382951817	4.16E-149
1	Nt5e	0.381963666	9.43E-115
1	Ddit4	0.380655562	3.71E-67
1	Kcnq1ot1	0.380441552	4.55E-83
1	Htra1	0.380103142	4.51E-123
1	Ptma	0.379553709	8.09E-112
1	Kcnn4	0.377385602	3.15E-145
1	Zfp503	0.377325922	8.06E-157
1	Dap	0.377019727	2.66E-71
1	Rps15a	0.376977591	9.59E-158
1	Polr2k	0.376759738	1.39E-63
1	Kank3	0.376635266	9.57E-146
1	Slc16a1	0.376142431	2.25E-108
1	Esyt2	0.375420569	4.65E-111
1	Tia1	0.374848673	1.09E-83
1	Etnk1	0.374557014	2.58E-80
1	Rpl9	0.373719581	9.24E-164
1	Igf1r	0.37335323	1.25E-109
1	Rasa1	0.373285813	1.32E-90
1	Rpl10	0.373277059	8.62E-125
1	Scx	0.373079516	6.44E-212
1	Rpl3	0.372658342	6.32E-91
1	Ctxn1	0.371540223	2.01E-200
1	Epha2	0.370452828	6.19E-141
1	Tbca	0.370024353	1.33E-85
1	Celf4	0.368759384	7.62E-199
1	Trip6	0.368054392	5.75E-155
1	Rps27	0.36802216	1.59E-175
1	Myl12a	0.366450077	6.84E-67
1	Ktn1	0.365387135	2.66E-93
1	Snrpg	0.364703465	1.24E-80
1	Azin2	0.36395398	5.79E-139
1	Jup	0.363711357	4.30E-132
1	Lamc2	0.363679202	2.78E-149
1	Ift43	0.36342444	8.83E-126
1	Aig1	0.363349207	1.95E-78
1	RP24-439I22.3	0.362831966	1.25E-72
1	Unc5b	0.361858164	9.57E-191
1	Hnrnpa1	0.361164512	1.39E-53

1	Tspan3	0.361146998	2.88E-75
1	Cd82	0.360836821	9.19E-119
1	Calm2	0.360399302	1.80E-73
1	Eif3h	0.360387235	1.06E-72
1	Plpp2	0.360204749	7.88E-142
1	Paics	0.359710587	4.80E-76
1	Stt3a	0.3593963	1.27E-62
1	Zwint	0.358954898	7.76E-90
1	Tfap2a	0.358860599	3.03E-231
1	Ier2	0.358518565	9.20E-19
1	Nop58	0.358197971	2.61E-70
1	Ift172	0.356712608	3.28E-156
1	Nfe2l1	0.356509987	6.66E-95
1	Igf2bp2	0.356031635	1.04E-194
1	Atpif1	0.355496751	2.94E-61
1	Dmp1	0.355459755	5.93E-201
1	Snhg6	0.354947436	1.15E-64
1	Rpl34	0.354500244	9.55E-158
1	Mgat4b	0.354305707	2.04E-100
1	Cd63	0.352841204	9.46E-66
1	Nr2f1	0.352523266	2.88E-162
1	Serf1	0.350678389	1.38E-78
1	Aimp1	0.349918277	9.19E-70
1	Rpl7	0.348714735	1.70E-106
1	Mrps6	0.34845036	1.67E-101
1	Fnbp1l	0.348353298	9.55E-159
1	Psip1	0.348183207	1.60E-97
1	Ncl	0.347928263	3.60E-48
1	Wdr36	0.34784849	6.03E-119
1	Hsd17b10	0.347320864	6.64E-75
1	Zfp706	0.346117058	3.25E-54
1	Srsf11	0.346114042	1.03E-58
1	RP23-8J15.4	0.345548307	0.001092
1	Gnb2l1	0.345273285	2.21E-108
1	Gm9864	0.345161404	5.32E-192
1	Cldn7	0.345152022	3.20E-171
1	Rps5	0.344023945	6.21E-137
1	Itpr3	0.342918481	2.54E-159
1	Ccbe1	0.341664003	3.17E-171
1	Rpl21	0.341069888	1.59E-116
1	Efna1	0.339953942	2.38E-172
1	Sema3f	0.338975389	6.31E-236
1	Tsc22d2	0.338958212	4.28E-91
1	Polr2e	0.338532177	6.90E-63
1	Ip6k2	0.338306458	3.22E-120
1	Lsr	0.337834222	2.27E-147
1	Rps8	0.336786171	9.82E-139
1	Mrpl13	0.336381023	3.22E-73
1	Ahnak2	0.336267065	1.34E-149
1	Wdr60	0.336085554	3.37E-141
1	Pdgfa	0.335932442	1.41E-102
1	Pnrc1	0.335440684	4.19E-41
1	Rps6	0.335262049	2.66E-93
1	Ifi203	0.335241214	7.36E-56
1	Sod1	0.334646369	7.10E-77
1	Npdc1	0.334645471	5.81E-137
1	RP23-263B18.4	0.334098672	4.84E-59
1	Rpsa	0.333827525	5.89E-93
1	Cxadr	0.333822956	1.06E-205
1	Ywhaq	0.333446253	7.82E-48
1	Clip4	0.333283513	1.05E-126

1	Zfhx3	0.333219646	7.77E-61
1	Rps26-ps1	0.33305954	1.47E-57
1	Arl2	0.333051867	1.73E-80
1	Srek1ip1	0.332655745	3.96E-81
1	Hspb8	0.331230265	4.45E-114
1	Nav1	0.331202523	6.26E-103
1	Gpa33	0.330833722	3.02E-223
1	Cdc42ep1	0.33049725	2.65E-191
1	Pola2	0.33018489	6.36E-10
1	Smg6	0.329287069	8.69E-128
1	RP23-269H21.1	0.329029465	5.66E-65
1	Ano1	0.328860132	1.06E-182
1	Thap3	0.32851411	4.75E-95
1	Vmp1	0.327834497	1.30E-25
1	Rpl36a	0.326967154	7.38E-101
1	Cfdp1	0.326116953	2.24E-63
1	Sgms2	0.325597068	6.04E-137
1	Aldh2	0.32513669	6.91E-53
1	Spint1	0.324395861	7.85E-216
1	Srpr	0.323794893	4.49E-85
1	Prkca	0.323368691	4.76E-146
1	Eif3l	0.323085963	3.94E-65
2	Cxcl3	1.9372	2.88E-58
2	Plac8	1.8349	4.10E-179
2	Srgn	1.4065	1.05E-234
2	Thbs1	1.3611	6.66E-37
2	Lgals3	1.1852	3.90E-217
2	Cd14	1.1207	5.80E-137
2	Cxcl2	1.1130	2.32E-51
2	Ccr2	1.1094	2.01E-143
2	Ccl24	1.1004	1.65E-23
2	Tgfbi	1.0741	1.24E-113
2	F10	1.0734	7.05E-135
2	Inhba	1.0362	1.68E-07
2	Tgm2	1.0261	2.36E-53
2	Ly6c2	1.0021	6.49E-110
2	Hilpda	0.9774	5.99E-34
2	Plaur	0.9765	3.91E-82
2	Il1b	0.9370	1.98E-76
2	Ms4a4c	0.9248	2.78E-63
2	Cd44	0.9205	3.44E-87
2	Rgcc	0.9118	3.94E-49
2	Btg1	0.8852	1.77E-130
2	Clec4e	0.8745	2.78E-102
2	Cebpb	0.8328	4.25E-181
2	Ptgs2	0.8319	6.72E-20
2	Alox5ap	0.8274	2.60E-132
2	Ero1l	0.8199	9.82E-35
2	Adam8	0.7999	7.72E-60
2	Ltb4r1	0.7956	1.30E-127
2	Fam49b	0.7941	3.52E-100
2	Cd53	0.7893	4.51E-135
2	Ccl9	0.7856	2.28E-69
2	Clec4d	0.7603	7.35E-92
2	Arg1	0.7458	1.62E-10
2	Napsa	0.7407	7.83E-107
2	Bnip3	0.7379	3.91E-25
2	Id2	0.7327	2.28E-29
2	Lst1	0.7287	7.84E-58
2	Fcer1g	0.7172	1.85E-128
2	Cd52	0.7163	8.25E-100

2	Anxa2	0.7147	8.52E-79
2	Tyrobp	0.7143	1.36E-130
2	Fxyd5	0.7095	1.40E-113
2	H2-Ab1	0.7090	4.77E-18
2	Smox	0.7089	4.83E-33
2	Pim1	0.7067	6.09E-65
2	Hopx	0.6980	3.89E-61
2	Pgk1	0.6976	7.90E-68
2	Plin2	0.6939	1.70E-48
2	Hp	0.6916	9.23E-72
2	Picalm	0.6898	8.24E-93
2	Arg2	0.6850	2.68E-54
2	Card19	0.6845	8.49E-39
2	Itgam	0.6814	9.13E-54
2	H2-Aa	0.6760	2.07E-09
2	Vegfa	0.6740	6.43E-29
2	Coro1a	0.6727	1.20E-66
2	Cd74	0.6703	7.59E-29
2	Lilr4b	0.6631	2.67E-58
2	Csf2rb	0.6535	1.06E-48
2	Osm	0.6508	4.01E-28
2	Mmp19	0.6427	2.58E-24
2	Emilin2	0.6371	5.70E-71
2	Cytip	0.6288	4.91E-48
2	Mcl1	0.6268	1.51E-80
2	Fem1c	0.6210	1.03E-34
2	Zeb2	0.6164	2.16E-44
2	Tgfb1	0.6159	1.15E-28
2	Clec7a	0.6079	2.11E-28
2	C5ar1	0.6069	2.65E-40
2	Ms4a6d	0.6039	3.82E-50
2	Crip1	0.6033	2.13E-62
2	Msrb1	0.5994	1.67E-52
2	Jarid2	0.5959	3.79E-36
2	Basp1	0.5956	6.38E-22
2	Arrdc4	0.5929	2.01E-16
2	Psap	0.5920	6.99E-110
2	Socs3	0.5904	1.76E-11
2	Ccr1	0.5879	6.64E-36
2	Slfn2	0.581433709	1.03E-25
2	Slc2a1	0.580001129	7.04E-15
2	Itgb2	0.578229404	5.19E-37
2	Malt1	0.57708651	1.54E-14
2	Sirpb1c	0.576999561	3.88E-53
2	S100a4	0.573393972	2.96E-33
2	Fosl2	0.573209642	3.23E-28
2	Fcgr3	0.570921291	5.12E-62
2	Ifi27l2a	0.563395693	3.00E-42
2	Prdx5	0.557064933	1.31E-75
2	Eno1	0.539367676	1.98E-53
2	Cybb	0.53742022	5.12E-24
2	Cstb	0.536543274	1.60E-52
2	Xist	0.536388646	3.08E-70
2	Agpat9	0.533974479	1.75E-43
2	Cdkn1a	0.529496154	3.40E-48
2	Kdm7a	0.528453121	1.54E-15
2	Clec4a1	0.528065586	2.64E-21
2	Ap3s1	0.524466827	1.13E-15
2	Neat1	0.523960169	2.11E-36
2	Glrx	0.523387806	2.23E-13
2	Pkm	0.522345233	8.34E-78

2	Lrrfip1	0.51548049	3.61E-10
2	Hif1a	0.515229389	5.85E-29
2	Ly6e	0.508355844	0.001509
2	Adgre5	0.504037871	1.45E-17
2	Gch1	0.501927644	1.52E-20
2	Rnh1	0.496275589	3.32E-11
2	Smpdl3a	0.49444655	9.65E-18
2	Tpd52	0.493015172	1.37E-34
2	Gngt2	0.491869383	6.41E-08
2	Gm2a	0.491112737	1.41E-29
2	Fth1.1	0.490554159	2.02E-29
2	Ier3	0.489343124	7.30E-30
2	Adssl1	0.488680846	1.23E-22
2	Fbxl5	0.488045051	5.11E-09
2	Fcgr1	0.487795682	5.12E-14
2	Actb	0.486522573	9.39E-42
2	Gpx1	0.485455416	6.13E-65
2	Ccr5	0.485262405	1.45E-22
2	Pycard	0.482431288	5.76E-18
2	Cd68	0.481752665	2.81E-27
2	H2-Ea-ps	0.481453575	6.49E-07
2	P4hb	0.481344389	4.81E-31
2	Soat1	0.480633597	1.76E-11
2	Actg1	0.47934948	6.12E-67
2	Nfil3	0.47895099	3.86E-13
2	Clec4a3	0.476727904	0.008964
2	Eif4ebp1	0.473715936	8.94E-16
2	RP24-176A1.3	0.472246071	8.49E-09
2	Gpi1	0.471918957	3.85E-19
2	Pid1	0.469427068	3.39E-17
2	Ftl1	0.461288944	5.28E-62
2	Bst1	0.460896689	4.15E-26
2	Cd300lf	0.46029555	2.78E-34
2	Tes	0.459828956	7.01E-09
2	Ndrg1	0.459249314	0.000394
2	Furin	0.458166913	2.26E-09
2	Gapdh	0.457133364	8.01E-38
2	Grina	0.453339534	1.56E-17
2	Il1rn	0.452745295	4.69E-10
2	Ccl6	0.448781787	4.07E-25
2	Prdx6	0.446433896	4.17E-06
2	Ptpn1	0.440683068	0.006499
2	Cox17	0.440554406	4.03E-30
2	Taldo1	0.438505067	5.07E-15
2	Igsf6	0.438346152	7.03E-22
2	Runx3	0.436806791	1.37E-13
2	Slc16a3	0.436309242	9.95E-22
2	H2-DMa	0.435247988	9.05E-17
2	Ncf2	0.434308744	7.13E-16
2	Plek	0.433305854	1.55E-28
2	Cyba	0.429018236	4.64E-62
2	Tmem189	0.428817119	2.55E-08
2	Snx18	0.427818816	1.87E-07
2	Rab20	0.425783647	1.89E-11
2	Rbms1	0.424039442	2.65E-18
2	Cxcr4	0.421953997	5.18E-18
2	RP24-344N22.3	0.419440142	7.71E-14
2	Mxi1	0.419091372	0.00465
2	Mafb	0.418326804	1.85E-15
2	Capza2	0.416190163	1.35E-25
2	Gna13	0.415084357	0.000441

2	Atox1	0.41498751	7.72E-38
2	Ptprc	0.414610551	5.24E-27
2	Chd7	0.411906675	0.001071
2	S100a11	0.41033962	2.42E-40
2	Tspo	0.409338338	5.48E-37
2	Sat1	0.408601476	5.10E-29
2	Sdcbp	0.406750859	3.56E-49
2	Emb	0.406316592	5.67E-17
2	Zfp36l1	0.404025544	2.98E-06
2	Lcp1	0.403971857	2.57E-28
2	Samhd1	0.402643339	0.003217
2	Laptm5	0.399359991	4.46E-37
2	Tpi1	0.399217769	5.83E-20
2	Aldoa	0.398986268	1.25E-36
2	Pgam1	0.398088034	1.55E-34
2	Mgst1	0.397879844	0.004715
2	Nfkbia	0.394695707	7.51E-09
2	Ifitm3	0.394054931	1.16E-23
2	Vamp4	0.394048106	5.39E-05
2	Spi1	0.392564001	4.92E-18
2	Ube2a	0.39176184	0.00017
2	Tm6sf1	0.388070792	2.20E-05
2	Lrp1	0.38798654	1.90E-06
2	Arpc2	0.387505552	2.08E-47
2	Pfkp	0.385317919	5.13E-05
2	Ncf4	0.38271569	1.35E-16
2	Pde4b	0.37887531	2.27E-06
2	H3f3a	0.377020606	4.02E-52
2	Emp1	0.376966871	1.61E-09
2	RP23-411N10.2	0.376437266	1.61E-09
2	Higd1a	0.374640439	1.66E-11
2	Arpc5	0.374224889	1.72E-27
2	Mkrn1	0.370369614	0.00112
2	Pla2g7	0.368290561	7.63E-07
2	Sh3bgrl3	0.36764837	6.41E-30
2	Irf5	0.366348995	0.000177
2	Csf2rb2	0.366280893	5.19E-09
2	Cndp2	0.365594256	1.07E-10
2	Gpr35	0.36462917	9.67E-09
2	Gda	0.364314215	1.04E-11
2	Ptbp3	0.361054902	6.00E-08
2	Iqgap1	0.359505984	9.64E-11
2	Lsp1	0.358662338	0.004747
2	RP23-264H19.2	0.358555795	1.66E-05
2	Fau	0.358524118	1.03E-105
2	Vsir	0.357203147	2.75E-09
2	Milr1	0.355992455	0.000713
2	Mpeg1	0.352167333	9.51E-12
2	Esd	0.351670534	0.013509
2	Emp3	0.35126379	3.00E-07
2	Capg	0.350084645	1.62E-12
2	Actr3	0.349188458	1.16E-10
2	Ostf1	0.343530992	1.03E-20
2	Malat1	0.342881273	1.24E-67
2	Lyn	0.340559245	7.62E-05
2	Sem1	0.33911875	8.18E-53
2	Vim	0.338969311	1.00E-42
2	Litaf	0.337679902	6.62E-12
2	Crem	0.335958556	0.000377
2	Gmfg	0.334642056	3.41E-08
2	Chmp4b	0.333817868	2.02E-05

2	Ninj1	0.332432009	0.0075
2	Slc15a3	0.331699894	0.008028
2	Npc2	0.331629802	1.33E-26
2	Msr1	0.330610366	2.44E-15
2	Mrpl52	0.330454667	4.35E-17
2	RP23-381B19.2	0.325831963	0.007238
2	Atp2b1	0.324193934	8.88E-10
2	Lyz2	0.324177371	2.25E-36
3	Ccl8	3.0525	2.82E-268
3	Cd163	2.2977	0
3	Sepp1	2.0442	1.60E-277
3	Mrc1	1.9200	1.63E-276
3	Wfdc17	1.7672	1.04E-190
3	Cbr2	1.6421	0
3	Ccl7	1.6363	1.06E-149
3	Rnase2a	1.6343	7.51E-86
3	Hmox1	1.6249	2.67E-146
3	Gas6	1.5637	0
3	Fcgrt	1.5414	1.82E-253
3	Maf	1.5210	4.43E-258
3	Pf4	1.4747	5.29E-175
3	Folr2	1.4332	1.86E-284
3	Fcna	1.4260	2.26E-193
3	Ms4a7	1.3480	9.13E-219
3	RP24-286C21.1	1.3459	2.56E-170
3	Ccl6	1.3335	1.76E-170
3	Npl	1.3234	1.88E-293
3	F13a1	1.3086	3.06E-123
3	Lgmn	1.3067	4.56E-228
3	Stab1	1.2910	4.78E-200
3	Ctsb	1.2746	3.05E-244
3	Fcgr2b	1.2242	9.76E-220
3	Ednrb	1.2159	2.98E-228
3	Ccl12	1.1994	2.35E-112
3	Dab2	1.1766	8.05E-173
3	Ccl9	1.1254	4.74E-150
3	Ccl2	1.0924	4.17E-70
3	Rnase4	1.0764	5.26E-145
3	Mafb	1.0537	1.41E-135
3	Fabp5	1.0291	8.25E-65
3	C1qa	1.0272	3.87E-139
3	Il4ra	1.0185	4.39E-122
3	Grn	0.9867	8.26E-176
3	C1qc	0.9758	1.88E-131
3	Timp2	0.9718	5.72E-121
3	Ap2a2	0.9597	3.92E-102
3	Ctsd	0.9468	2.24E-142
3	Ltc4s	0.9290	7.24E-67
3	Cela1	0.9265	4.33E-167
3	Lamp1	0.9235	4.01E-168
3	Tmem8	0.9186	2.12E-223
3	Fcrls	0.9067	2.53E-72
3	Apoe	0.9061	1.45E-118
3	Trem2	0.8867	1.80E-108
3	Ap1b1	0.8814	4.28E-117
3	Ninj1	0.8778	8.08E-93
3	Cltc	0.8737	8.76E-105
3	Stard8	0.8699	1.29E-162
3	Snx2	0.8671	3.21E-92
3	Glul	0.8621	6.38E-72
3	Pltp	0.8607	2.79E-84

3	Ccl4	0.8455	6.24E-14
3	Ctsl	0.8399	2.10E-110
3	Ctsa	0.8285	3.26E-120
3	Tpp1	0.8129	1.79E-91
3	Mfsd1	0.8106	7.26E-94
3	Ch25h	0.8041	7.71E-46
3	Itm2b	0.7976	2.02E-145
3	Hexa	0.7968	8.95E-103
3	C3ar1	0.7950	1.99E-91
3	C1qb	0.7895	4.29E-93
3	Sgpl1	0.7883	1.14E-79
3	Klf4	0.7791	4.62E-40
3	Igf1	0.7561	5.82E-104
3	Gda	0.7553	7.18E-83
3	Ms4a6d	0.7532	2.71E-93
3	Tcn2	0.7477	9.36E-69
3	Frmd4b	0.7305	2.36E-89
3	Ifi27l2a	0.7275	2.92E-15
3	Bmp2k	0.7230	8.45E-71
3	Nxpe5	0.7215	8.74E-135
3	Egr1	0.7131	4.92E-15
3	Cfh	0.7104	1.24E-42
3	Plau	0.7080	1.22E-27
3	Adgre1	0.7078	4.08E-84
3	Ap2s1	0.7069	2.47E-87
3	Eps15	0.7004	3.68E-69
3	Chchd10	0.6897	2.28E-12
3	Atp6v1a	0.6772	1.34E-62
3	Wwp1	0.6687	2.23E-50
3	Qk	0.6641	3.49E-53
3	Ms4a6c	0.6583	4.29E-83
3	Gns	0.6572	6.15E-51
3	Abca1	0.6539	1.61E-54
3	Ap2m1	0.6521	4.48E-57
3	Ifi207	0.6491	1.78E-47
3	Dhrs3	0.6458	2.93E-59
3	Scarb2	0.6444	5.09E-41
3	Laptm5	0.6441	1.41E-78
3	Csf1r	0.6420	1.08E-72
3	Man1a	0.6398	4.18E-45
3	Snx5	0.6383	5.90E-57
3	Sdc3	0.6358	1.03E-53
3	Plek	0.6350	1.02E-48
3	Hal	0.6324	6.67E-30
3	Slc7a8	0.6282	7.89E-80
3	Sat1	0.6278	1.05E-80
3	Pmp22	0.6263	6.11E-17
3	Hsbp1	0.6243	2.90E-55
3	Qsox1	0.6237	4.63E-43
3	Ehd4	0.6210	1.25E-51
3	Atp6ap1	0.6209	1.38E-57
3	Emp1	0.6179	3.45E-47
3	Adam9	0.6154	4.13E-49
3	Fam213b	0.6144	8.66E-59
3	Rab1a	0.6088	2.56E-56
3	Pirb	0.6008	1.80E-52
3	Ndufa1	0.6000	2.53E-15
3	Fchsd2	0.5972	4.91E-69
3	Itsn1	0.5955	2.42E-54
3	Gnpda1	0.5940	8.00E-45
3	Arl8a	0.5916	2.65E-40

3	Arg1	0.584182828	1.15E-38
3	Creg1	0.583519439	3.25E-42
3	Mcfd2	0.583350047	1.05E-32
3	Rcn3	0.581280756	2.16E-68
3	Snx6	0.580987558	6.48E-40
3	Tns1	0.580604578	1.16E-74
3	Snx3	0.580065021	1.16E-56
3	Serinc3	0.578016116	1.66E-47
3	Snx8	0.57398645	1.92E-56
3	Rab11fip5	0.5735955	2.04E-45
3	Rassf2	0.57301233	1.33E-30
3	Osbpl9	0.572942663	1.68E-28
3	Clta	0.571671724	6.68E-85
3	RP23-92G13.4	0.569571759	7.11E-35
3	Bin1	0.568797561	1.17E-32
3	Pdpn	0.566923745	6.39E-30
3	Rap1b	0.565653421	2.33E-64
3	Sdcbp	0.564889756	1.24E-73
3	Sirpa	0.562512001	1.68E-42
3	Cd68	0.561044901	1.13E-44
3	Gatm	0.560543943	1.64E-40
3	Klf2	0.560421764	1.01E-09
3	Hebp1	0.559918548	3.51E-46
3	Nrp1	0.559397473	5.18E-60
3	Pitpna	0.558919689	5.75E-35
3	Rab11a	0.555474666	5.88E-49
3	Blvrb	0.55285483	1.44E-34
3	Cfp	0.552369577	1.32E-51
3	Mtss1	0.551277605	5.91E-30
3	Smagp	0.547488867	4.42E-105
3	Zfp36	0.547217426	5.70E-18
3	Kctd12	0.546863738	2.80E-34
3	Ftl1	0.543776549	1.29E-94
3	Slc43a2	0.542830245	2.88E-44
3	Pld3	0.540712137	1.31E-33
3	Furin	0.539612102	2.06E-17
3	Ctss	0.536896158	7.96E-62
3	Pla2g15	0.536366978	1.53E-48
3	Cd38	0.532013824	2.45E-34
3	Vat1	0.531133037	2.13E-27
3	Hspa1b	0.530620013	6.86E-15
3	Colec12	0.52883681	4.81E-36
3	Atp6v0b	0.526143915	7.31E-55
3	Plin2	0.523830631	4.25E-45
3	Cd84	0.518892262	5.22E-58
3	Rab3il1	0.515443924	5.92E-44
3	Sypl	0.514119104	2.03E-28
3	Stx12	0.513508191	2.24E-22
3	Atp6ap2	0.51289476	1.47E-39
3	Camk1	0.510271156	9.28E-27
3	Il10rb	0.509720516	3.82E-38
3	Cxcr4	0.508398107	1.98E-44
3	Tgfbr2	0.506824468	2.89E-43
3	Atp1b3	0.504792144	2.23E-32
3	Efhd2	0.502187262	2.24E-44
3	Acp2	0.501594603	3.16E-39
3	Tmem106a	0.500803718	2.89E-36
3	Tmem2	0.499928503	1.30E-30
3	Ptpn18	0.499884527	4.01E-46
3	Unc93b1	0.499357231	6.06E-38
3	Slc6a6	0.499324516	4.71E-30

3	Ccl3	0.498489563	3.43E-17
3	Cd53	0.495701436	1.83E-54
3	Fn1	0.494193235	4.68E-19
3	Cd200r1	0.493114331	7.37E-58
3	App	0.492978916	7.57E-48
3	Lamp2	0.491945637	2.19E-35
3	Fkbp15	0.490356533	1.19E-40
3	Scoc	0.489679364	1.66E-14
3	Tmem256	0.489007088	9.04E-56
3	Rgl1	0.487395896	2.25E-33
3	Rhob	0.486673188	2.92E-16
3	Clcn5	0.486386677	2.84E-55
3	Myo5a	0.484455168	5.78E-34
3	Gja1	0.483132686	1.38E-06
3	Adam15	0.480980003	1.79E-25
3	Arpc3	0.480466543	2.75E-48
3	Atp6v1b2	0.480064827	1.45E-30
3	Hpgds	0.477076878	1.15E-51
3	Itgam	0.476920211	1.91E-34
3	Rnf130	0.474660919	5.33E-32
3	Arap1	0.474498331	7.94E-30
3	Nrros	0.474209062	3.46E-35
3	Necap2	0.471728326	1.91E-25
3	Plk2	0.469837982	1.13E-12
3	Lyz2	0.468830602	5.17E-57
3	Gpt2	0.467652692	1.04E-72
3	Nrp2	0.465428514	4.75E-20
3	Secisbp2l	0.4639464	1.11E-26
3	RP23-29H5.3	0.460715708	1.34E-19
3	Slc9a9	0.457904632	6.08E-61
3	Mylip	0.457314869	1.40E-16
3	Cd93	0.457289986	2.19E-37
3	Klhl9	0.457154733	6.33E-13
3	Tmem141	0.455293016	6.92E-37
3	Pdia4	0.45316861	2.17E-19
3	Osm	0.451767621	9.54E-13
3	Tsc22d3	0.450945359	4.01E-15
3	Hspa1a	0.449924874	5.30E-30
3	Rab5c	0.449779357	2.86E-26
3	Hk2	0.44954191	2.50E-09
3	Hfe	0.448970585	1.71E-34
3	Wdfy2	0.446499351	2.39E-32
3	Amdhd2	0.44638221	2.93E-34
3	Vsir	0.445452812	1.06E-28
3	Wnk1	0.4443912	2.49E-31
3	Itga9	0.441519589	2.21E-49
3	Eps8	0.440378826	1.23E-11
3	8-Mar	0.440167087	1.99E-24
3	Snx27	0.437885222	1.44E-11
3	Tbxas1	0.437689317	1.98E-27
3	Fcgr3	0.437430462	5.83E-39
3	Siglec1	0.436518861	4.90E-34
3	Arl6ip1	0.434885337	3.94E-36
3	Slc48a1	0.432938734	0.006224
3	Metrnl	0.431610772	2.57E-24
3	Ap1s2	0.431320389	1.55E-27
3	Dazap2	0.43072221	7.02E-31
3	Rnasel	0.430287959	2.73E-42
3	Atp6v1h	0.427838709	4.85E-14
3	Itm2c	0.426874043	1.64E-21
3	Etv1	0.426797906	1.05E-16

3	Tbc1d14	0.426668122	2.45E-28
3	Dbi	0.424821961	4.29E-38
3	Aplp2	0.424332924	1.04E-15
3	Picalm	0.424101468	1.30E-35
3	Serpinb6a	0.424015023	5.56E-18
3	Lrg1	0.422097855	5.21E-57
3	Rcan1	0.419272062	2.15E-08
3	Ncf1	0.417665476	9.65E-21
3	Ssh2	0.41657402	7.22E-23
3	Enpp1	0.413185236	4.28E-47
3	Ehd1	0.41239725	3.65E-16
3	Abhd12	0.410324009	1.94E-17
3	Pla2g7	0.408778378	3.61E-25
3	Atp6v1g1	0.4081224	5.86E-54
3	Atp6v0a1	0.407487337	2.03E-35
3	Sash1	0.407012315	2.43E-24
3	Tmem37	0.406172159	1.68E-21
3	Tmem50a	0.405898398	1.20E-25
3	Ubl3	0.405743384	9.01E-22
3	Oxct1	0.405366269	1.43E-18
3	Cd302	0.405279074	2.02E-19
3	RP24-261H7.9	0.40353309	5.21E-21
3	Akr1a1	0.402834424	3.01E-34
3	Canx	0.402652749	2.97E-24
3	Arl8b	0.401997226	9.13E-18
3	Vamp8	0.40188037	3.35E-39
3	Atp6v0d1	0.400818191	4.81E-17
3	M6pr	0.398123172	3.32E-17
3	Rnf13	0.397632173	5.41E-14
3	Sh3bgrl	0.397059567	2.20E-14
3	Smpdl3a	0.395545647	1.58E-24
3	Fcer1g	0.395193474	5.60E-35
3	Cndp2	0.394623337	5.49E-22
3	Idh1	0.393984111	5.17E-11
3	Ctnnb1	0.393299045	5.89E-25
3	Slc7a7	0.391933762	7.58E-31
3	Pea15a	0.391597824	1.41E-09
3	Npc1	0.391565252	1.75E-22
3	Dbnl	0.39154946	8.08E-14
3	Rragc	0.390869007	4.87E-16
3	Mcl1	0.390546294	1.37E-29
3	Fam105a	0.390048425	2.08E-23
3	Klf6	0.389568197	6.46E-09
3	Sec14l1	0.38939791	9.37E-14
3	Pepd	0.388357003	1.10E-11
3	Dusp1	0.388331734	1.08E-05
3	Impact	0.388151773	6.85E-11
3	Tnfsf12	0.387937721	8.10E-34
3	Anpep	0.387055952	2.88E-20
3	Gna12	0.386660942	9.48E-14
3	Cops9	0.386379465	7.67E-35
3	Blvra	0.38561138	5.37E-12
3	Ptprj	0.384984849	2.50E-24
3	Marcks	0.384064333	2.82E-20
3	Atp6v1e1	0.382989237	2.31E-19
3	RP23-440D19.2	0.379998444	3.95E-12
3	Sdc4	0.377913937	2.06E-21
3	Mef2a	0.377902923	2.87E-13
3	Mgat1	0.377179051	6.24E-14
3	Plekhb2	0.377034043	3.14E-12
3	Zeb2	0.376830329	9.70E-17

3	Slc12a7	0.376808034	1.16E-21
3	Hip1	0.376096916	4.40E-10
3	C5ar1	0.375523507	6.75E-36
3	Rhoq	0.373342138	2.71E-09
3	Sgpp1	0.372151896	4.37E-08
3	Dpp7	0.371753877	1.24E-30
3	Ptbp3	0.371522924	7.40E-14
3	Tgoln1	0.371469839	1.26E-19
3	RP24-482L4.3	0.371265029	1.10E-35
3	Dennd5a	0.371173639	2.04E-06
3	Scamp2	0.370970369	1.69E-06
3	Srgn	0.370270325	1.68E-17
3	Gusb	0.366781627	2.22E-21
3	Fcho2	0.366737386	5.27E-16
3	Fam63a	0.366410085	7.20E-15
3	Rab7	0.364422821	2.97E-19
3	Cmtm6	0.363872905	3.34E-05
3	Zfp36l1	0.362894651	6.38E-21
3	Sh3glb1	0.362267787	3.93E-19
3	Rin2	0.3613675	8.14E-06
3	Cab39	0.358805365	5.80E-11
3	Sulf2	0.358640211	8.35E-06
3	Acp5	0.358132107	1.17E-06
3	Hacd4	0.35800197	2.07E-14
3	Aftph	0.357055236	4.65E-11
3	Hsp90b1	0.356709007	4.74E-19
3	Lipa	0.356684246	5.22E-13
3	Plod1	0.356640995	6.94E-15
3	Dcxr	0.35645831	1.01E-19
3	Gltp	0.356203445	8.47E-14
3	Anxa4	0.355556118	3.72E-10
3	Rab31	0.35490174	5.41E-06
3	Cdc42	0.354691099	1.17E-39
3	Irf2bp2	0.351777652	2.95E-16
3	Atp13a2	0.35173872	6.95E-22
3	Rab14	0.350737745	1.47E-15
3	Vamp3	0.35005221	3.31E-14
3	Uap1l1	0.348415285	5.89E-10
3	St6gal1	0.348354272	4.08E-17
3	P2rx4	0.347700553	7.44E-10
3	Twf1	0.346780998	9.38E-11
3	Ctsz	0.345752451	1.25E-30
3	Neu1	0.345410384	2.18E-12
3	Renbp	0.344175422	5.27E-17
3	Scamp1	0.343294374	2.87E-12
3	Agtrap	0.341811357	1.98E-06
3	Hist1h1c	0.341152228	3.43E-08
3	Sptssa	0.340215291	4.26E-13
3	Man1c1	0.340139919	1.02E-22
3	Mpp1	0.339497771	2.96E-12
3	Tmem86a	0.339439305	1.21E-18
3	Tnfrsf21	0.338412388	4.19E-16
3	Bach1	0.336906133	1.77E-07
3	Ncoa4	0.336833542	7.77E-10
3	Nceh1	0.335623408	1.87E-11
3	Ccr5	0.335316894	2.50E-13
3	P2ry6	0.334316089	3.27E-12
3	Pdia6	0.334029446	1.65E-13
3	Vps4b	0.333990555	1.07E-05
3	Wdr26	0.333805452	2.29E-11
3	Hbegf	0.333241558	0.005741

3	Gpr146	0.333025797	1.52E-19
3	Zfand5	0.331303536	2.81E-09
3	Hist1h2bc	0.329019266	8.59E-18
3	Dnase2a	0.328926566	6.69E-10
3	Tmem30a	0.32889987	2.10E-05
3	Cebpa	0.328526541	4.71E-16
3	Gng12	0.327636888	1.28E-09
3	Gnb1	0.327305309	8.55E-13
3	Inpp5d	0.326297618	2.54E-22
3	Osbpl11	0.325880187	1.89E-21
3	Flcn	0.325395836	3.80E-10
3	Snx9	0.32401749	7.76E-06
4	Col1a2	3.9462	0
4	Col1a1	3.8578	0
4	Col3a1	3.8461	0
4	Sparc	3.7649	0
4	Rarres2	3.1105	0
4	Dcn	3.0747	0
4	Fstl1	2.8948	0
4	Bgn	2.8372	0
4	Mfap5	2.6721	0
4	Gpx3	2.6031	0
4	Postn	2.5398	0
4	Col5a2	2.5390	0
4	Serpinh1	2.4981	0
4	Meg3	2.4429	0
4	Ccl11	2.4070	0
4	Timp1	2.3986	4.69E-270
4	Serpinf1	2.2706	0
4	Pi16	2.2671	1.10E-299
4	Aebp1	2.2610	0
4	Serping1	2.2438	0
4	Ly6a	2.2081	0
4	Clec3b	2.2024	0
4	Thbs2	2.1436	0
4	Lox	2.1353	0
4	Col12a1	2.1070	0
4	Fbn1	2.0939	0
4	Igfbp7	2.0800	0
4	Col6a3	2.0749	0
4	Tnc	2.0636	5.96E-235
4	Saa3	2.0475	1.41E-38
4	C3	1.9993	3.19E-211
4	Dpt	1.9879	0
4	Htra3	1.9803	0
4	Cthrc1	1.9735	0
4	Sfrp2	1.9593	0
4	Mmp2	1.9527	0
4	Serpine1	1.9510	5.28E-211
4	Fbln2	1.9472	0
4	Lum	1.9453	0
4	Col5a1	1.9262	0
4	Ptx3	1.9168	0
4	Col6a2	1.9122	0
4	Cpxm1	1.9094	0
4	Gsn	1.8956	1.73E-143
4	Col14a1	1.8906	0
4	Thy1	1.8654	0
4	Col6a1	1.8481	0
4	Pcolce	1.8038	0
4	Loxl1	1.7745	0

4	Acta2	1.7583	1.41E-182
4	Ly6c1	1.7101	1.38E-238
4	Ppic	1.7003	0
4	Nid1	1.6952	0
4	Tagln	1.6747	1.83E-272
4	Lpl	1.6625	0
4	Nbl1	1.6464	0
4	Csrp2	1.5916	0
4	Ccdc80	1.5904	0
4	Rcn3	1.5738	0
4	Col5a3	1.5725	0
4	Sod3	1.5717	0
4	Itm2a	1.5679	0
4	Snhg18	1.5307	0
4	Cyr61	1.5102	0
4	Wisp2	1.5028	0
4	Ckap4	1.4590	0
4	Prss23	1.4490	0
4	Igfbp6	1.4441	6.03E-272
4	S100a16	1.4424	0
4	Plat	1.4401	0
4	Igfbp4	1.4385	1.48E-79
4	Fibin	1.4301	0
4	Mmp3	1.4237	1.28E-124
4	Mt2	1.4016	6.16E-99
4	Prrx1	1.3975	0
4	Tnfaip6	1.3871	0
4	Selm	1.3845	0
4	Gas1	1.3743	0
4	Serpina3n	1.3591	0
4	Lbp	1.3585	0
4	Aspn	1.3446	0
4	Cxcl12	1.3430	0
4	Ogn	1.3360	0
4	Efemp2	1.3350	0
4	Loxl2	1.3121	0
4	Tgfbr2	1.2878	5.35E-204
4	Egr1	1.2759	4.73E-176
4	Cxcl14	1.2591	3.82E-17
4	Cd34	1.2560	0
4	Tpm2	1.2438	0
4	Sparcl1	1.2420	0
4	C4b	1.2412	0
4	Sfrp4	1.2350	1.36E-300
4	Adamts2	1.2344	0
4	RP23-99G21.3	1.2325	0
4	Il11ra1	1.2279	0
4	C1qtnf6	1.2224	0
4	Col8a1	1.2217	1.37E-153
4	Hspb1	1.2174	1.52E-190
4	Abi3bp	1.2065	0
4	Cald1	1.2041	2.30E-249
4	Adamts5	1.2021	0
4	Tuba1a	1.2012	3.14E-202
4	Ebf1	1.1981	0
4	Pdgfra	1.1934	0
4	Bmp1	1.1929	0
4	Ctgf	1.1905	1.83E-240
4	Tmed3	1.1877	6.42E-244
4	Gadd45g	1.1801	1.60E-159
4	C1s1	1.1795	0

4	Tmem45a	1.1761	0
4	Il33	1.1726	0
4	Kdelr2	1.1715	9.01E-200
4	Rcn1	1.1707	0
4	Plod2	1.1586	0
4	Rian	1.1528	0
4	Ndufa4l2	1.1512	1.31E-225
4	Ctsk	1.1478	0
4	Pla1a	1.1380	0
4	Gpx8	1.1268	0
4	Rnase4	1.1233	7.83E-198
4	Mfap2	1.1224	0
4	Gpx7	1.1217	0
4	Tnxb	1.1180	0
4	Cd248	1.1024	0
4	Vkorc1	1.0955	7.64E-262
4	Eln	1.0912	3.96E-273
4	Mmp23	1.0887	0
4	Prkcdbp	1.0867	1.75E-273
4	Fgfr1	1.0847	0
4	Timp3	1.0843	0
4	Angptl4	1.0833	0
4	Itih5	1.0832	0
4	Prelp	1.0832	0
4	Calu	1.0662	1.21E-250
4	Nedd4	1.0628	2.29E-277
4	Pdpn	1.0628	1.11E-296
4	Fndc1	1.0612	0
4	Mgst1	1.0583	3.02E-199
4	Serpine2	1.0574	4.91E-57
4	Fkbp9	1.0527	0
4	Sfrp1	1.0478	4.53E-141
4	Spon1	1.0463	9.57E-110
4	Fkbp7	1.0432	0
4	Antxr1	1.0303	0
4	Plpp3	1.0268	0
4	Rhoj	1.0245	0
4	Fbln1	1.0236	0
4	Heg1	1.0225	0
4	Medag	1.0152	0
4	Lrrc17	1.0124	0
4	Efemp1	1.0122	0
4	Ptrf	1.0101	8.10E-219
4	Fkbp10	1.0095	0
4	Kdelr3	1.0093	0
4	Bicc1	0.9915	0
4	Fkbp11	0.9908	6.87E-283
4	Tceal8	0.9812	8.85E-257
4	Copz2	0.9804	0
4	Cygb	0.9718	0
4	Olfml3	0.9700	0
4	Tpm1	0.9670	4.23E-130
4	Hspg2	0.9648	0
4	Lrrc15	0.9527	0
4	Pcsk5	0.9477	0
4	Svep1	0.9377	0
4	Lhfp	0.9313	0
4	Rbms3	0.9190	0
4	Sulf1	0.9170	0
4	Creb3l1	0.9146	0
4	Ugdh	0.9120	3.50E-166

4	Mmp14	0.9046	2.90E-175
4	Csrp1	0.9010	5.62E-100
4	Grb10	0.9002	0
4	Nrep	0.9000	0
4	Cxcl1	0.8981	9.99E-66
4	Ddah1	0.8943	0
4	Htra1	0.8903	7.11E-211
4	Dclk1	0.8819	0
4	Serpinb6a	0.8792	5.73E-159
4	Fxyd1	0.8731	0
4	Txndc5	0.8709	1.05E-214
4	Prg4	0.8677	0
4	Tgfbr3	0.8659	0
4	Arf4	0.8636	1.11E-171
4	Car4	0.8630	4.55E-201
4	P4ha2	0.8499	0
4	Zbtb20	0.8490	7.14E-192
4	Oaf	0.8481	2.22E-172
4	Npdc1	0.8478	4.41E-281
4	Cdh11	0.8467	0
4	Vcan	0.8395	0
4	Ccl2	0.8361	3.86E-54
4	Palld	0.8295	1.24E-205
4	Cnn3	0.8247	1.83E-186
4	Gpm6b	0.8212	0
4	Nov	0.8205	1.80E-290
4	Pros1	0.8114	0
4	Mfap4	0.8045	0
4	Gnas	0.7944	1.69E-134
4	Figf	0.7914	0
4	Mrc2	0.7891	0
4	Tfpi2	0.7860	0
4	Ptgis	0.7860	0
4	C1ra	0.7850	0
4	Rbp1	0.7831	8.15E-151
4	Loxl3	0.7750	3.94E-216
4	Map1lc3a	0.7701	2.88E-157
4	Gstm2	0.7650	0
4	P3h3	0.7649	0
4	Cebpd	0.7576	3.33E-156
4	Errfi1	0.7567	6.79E-110
4	Mt1	0.7559	2.97E-18
4	Gpc3	0.7524	0
4	Ackr3	0.7508	1.97E-177
4	Fbn2	0.7495	0
4	Hsd11b1	0.7461	0
4	Sema3c	0.7451	0
4	Fst	0.7437	0
4	Adamts15	0.7420	0
4	Pmp22	0.7416	2.99E-243
4	Mfge8	0.7386	1.80E-156
4	Morf4l2	0.7381	2.12E-133
4	Col4a1	0.7378	2.10E-104
4	Nenf	0.7362	2.74E-130
4	Col16a1	0.7361	0
4	Maged2	0.7356	0
4	Klf9	0.7356	2.19E-302
4	Laptm4a	0.7328	1.54E-139
4	Tmem263	0.7299	9.47E-296
4	Tpm4	0.7285	1.59E-77
4	Igf1	0.7276	6.31E-119

4	Jun	0.7268	2.33E-86
4	Srpx2	0.7259	0
4	Fndc3b	0.7225	3.79E-157
4	Ikbip	0.7221	1.64E-260
4	Gng11	0.7215	3.03E-119
4	Adgrd1	0.7161	0
4	Crtap	0.7117	3.22E-194
4	Lrp1	0.7116	7.88E-131
4	Oat	0.7104	8.44E-171
4	Lama4	0.7097	0
4	Marcks	0.7097	2.43E-78
4	Nsg1	0.7066	0
4	Col15a1	0.7062	5.65E-290
4	Angptl2	0.7041	2.72E-174
4	Has1	0.7028	0
4	Scn7a	0.6973	0
4	Arl1	0.6943	6.68E-146
4	Gfpt2	0.6933	0
4	Pmepa1	0.6931	2.74E-90
4	Fn1	0.6902	2.90E-81
4	Fermt2	0.6898	2.84E-271
4	Sar1a	0.6881	7.99E-143
4	Vgll3	0.6877	0
4	Tceal9	0.6871	7.06E-117
4	Islr	0.6829	0
4	Cmah	0.6827	0
4	Gng12	0.6823	2.41E-127
4	Adamts1	0.6800	1.29E-178
4	Cryab	0.6740	7.10E-72
4	Lman1	0.6727	1.63E-132
4	Adm	0.6714	1.17E-104
4	Mxra8	0.6691	0
4	Gem	0.6689	6.42E-231
4	Cyp1b1	0.6684	0
4	Mtch1	0.6670	8.24E-122
4	Pxdn	0.6641	0
4	Tmem119	0.6640	5.25E-251
4	Il6st	0.6628	2.35E-188
4	Kdelr1	0.6613	2.96E-143
4	Cyp7b1	0.6604	0
4	Dpysl3	0.6599	0
4	Ostc	0.6564	5.56E-112
4	Dcbld2	0.6542	1.10E-213
4	Sptbn1	0.6518	2.78E-142
4	Wisp1	0.6512	0
4	Gas7	0.6489	1.24E-62
4	Olfml2b	0.6487	0
4	Slc25a4	0.6412	7.30E-107
4	Timp2	0.6410	4.06E-73
4	Vasn	0.6409	1.40E-286
4	Ost4	0.6393	4.08E-119
4	Gxylt2	0.6382	0
4	Ddr2	0.6376	5.93E-278
4	Spon2	0.6374	0
4	Slc43a3	0.6365	0
4	P4ha3	0.6343	0
4	Adamtsl1	0.6326	0
4	Lima1	0.6325	1.78E-256
4	Eif2s2	0.6288	1.29E-92
4	Prnp	0.6267	5.90E-142
4	Lpar1	0.6256	0

4	Rrbp1	0.6252	1.16E-103
4	Lamc1	0.6223	5.58E-195
4	Ccl7	0.6193	2.70E-79
4	Ly6e	0.6192	1.29E-94
4	Il1r1	0.6192	0
4	Plagl1	0.6178	0
4	Cd302	0.6164	1.07E-145
4	Chl1	0.6143	0
4	Cyb5a	0.6142	1.20E-102
4	Ssr3	0.6131	7.24E-104
4	Surf4	0.6105	6.75E-121
4	P3h1	0.6092	0
4	Adam12	0.6077	0
4	Tfpi	0.6038	0
4	Hdlbp	0.6030	8.33E-116
4	Pgf	0.6027	5.89E-22
4	Cyb5r3	0.6025	1.06E-128
4	Hes1	0.6022	1.35E-72
4	Ift20	0.6015	3.59E-124
4	Akr1b8	0.5987	1.14E-169
4	Fzd1	0.5976	0
4	Ssr2	0.5975	3.24E-83
4	Slit3	0.5967	0
4	Chpf	0.5963	2.18E-215
4	Arhgap29	0.5963	0
4	Ptk7	0.5961	1.92E-150
4	Sdc1	0.5899	1.92E-70
4	Pam	0.5889	4.91E-105
4	Ryk	0.5888	8.95E-227
4	Pofut2	0.5881	6.70E-152
4	Fos	0.5876	1.49E-40
4	Dusp1	0.5867	1.58E-33
4	Tppp3	0.5858	2.06E-62
4	P3h4	0.582540072	0
4	Apod	0.582115589	0
4	Slfn5	0.581666966	2.92E-137
4	Lsp1	0.58108191	6.91E-126
4	Cd55	0.57745162	0
4	Tshz2	0.57728272	0
4	Dad1	0.576765647	1.33E-88
4	Cd3e	0.57517899	2.61E-204
4	Flrt2	0.573355008	0
4	Sdc2	0.572665778	1.60E-156
4	Ppib	0.571720003	9.36E-91
4	Cd63	0.569803987	2.45E-78
4	Krtcap2	0.568960343	2.52E-102
4	Sec24d	0.565600687	2.02E-203
4	Pdlim1	0.563674756	1.26E-280
4	Gstm1	0.562406152	7.14E-118
4	Nfia	0.560648388	7.56E-103
4	Dlc1	0.5555693	0
4	Praf2	0.55491771	1.10E-207
4	Pdgfrb	0.554778404	0
4	Ptgfrn	0.55322079	2.63E-151
4	Thbs3	0.55299195	0
4	Yipf5	0.551605437	1.64E-149
4	Atf5	0.55042137	7.25E-157
4	Ncam1	0.550405161	0
4	Phlda3	0.549814041	3.56E-159
4	Parva	0.548279818	3.60E-228
4	Pdlim4	0.546598811	2.34E-184

4	Scara3	0.540669325	2.22E-199
4	Myl9	0.540288686	4.32E-253
4	Foxp1	0.539621902	1.35E-147
4	Frmd6	0.5392442	9.05E-230
4	Ak1	0.539033525	0
4	Uap1	0.538282515	7.51E-93
4	Dnajc25	0.537287679	3.85E-212
4	Ndn	0.537158276	0
4	Osmr	0.535902063	1.78E-226
4	Swi5	0.531209168	4.25E-83
4	Sepw1	0.529733373	6.83E-88
4	Hacd1	0.529527702	7.83E-181
4	Tmed2	0.528685094	3.99E-90
4	Tmem167	0.528266523	3.15E-90
4	Tspan6	0.528197693	0
4	Clmp	0.523587511	0
4	Entpd2	0.523393115	0
4	Eva1b	0.523307145	5.58E-97
4	Srpx	0.520670244	0
4	Nnmt	0.516182449	0
4	Pcolce2	0.515001854	2.35E-121
4	Egfr	0.514023208	0
4	Slc39a14	0.512136221	1.87E-98
4	Man2a1	0.509587292	4.95E-89
4	Lamb1	0.507932127	7.03E-148
4	Ext1	0.507022281	9.99E-124
4	Dram1	0.507013201	1.57E-265
4	Dpep1	0.505083036	0
4	Wipi1	0.504932321	5.13E-226
4	C1rb	0.504907537	0
4	Tmed9	0.504541029	1.34E-77
4	Maged1	0.501963513	2.38E-109
4	Wnt2	0.50030807	0
4	Itga5	0.499643288	1.37E-138
4	Cdc42ep5	0.499095889	9.14E-238
4	Glipr2	0.496963273	6.08E-161
4	Fam114a1	0.496635754	0
4	Lpp	0.496434177	6.37E-97
4	Nme2	0.493128915	1.19E-66
4	RP24-370G18.1	0.491947303	0
4	Tgfb1i1	0.491669951	0
4	Thbs1	0.489389899	3.74E-60
4	Adamts4	0.489387019	0
4	Crip2	0.486436972	4.51E-129
4	Crispld2	0.485161027	0
4	Scara5	0.484309765	2.11E-187
4	C1s2	0.482470833	0
4	Mxra7	0.481950844	0
4	Cpxm2	0.479437914	0
4	Emb	0.478794857	5.89E-48
4	Srm	0.478705244	3.92E-85
4	Ssr4	0.477097107	5.09E-68
4	Gpr153	0.476931069	0
4	Lrrn4cl	0.475777147	0
4	Slc39a1	0.474018661	1.25E-60
4	Igfbp3	0.473296595	1.72E-149
4	En1	0.473036983	0
4	Fhl1	0.472496021	0
4	Rbfox2	0.471988097	3.05E-178
4	Col4a2	0.470818556	8.32E-127
4	Fscn1	0.468268553	5.93E-162

4	Rpl39l	0.466733956	0
4	Rbpms	0.466199818	0
4	Pgrmc1	0.465966103	4.86E-98
4	Twist1	0.465075763	1.28E-194
4	Cnih1	0.464518021	2.96E-101
4	Ociad2	0.463082475	1.56E-229
4	Mnda	0.460609769	3.50E-56
4	Tmem100	0.460105135	0
4	Nfix	0.459938241	8.93E-117
4	Col6a5	0.458012657	0
4	Kctd11	0.457913109	5.50E-182
4	Vimp	0.457911924	6.70E-71
4	Slc38a10	0.457318615	3.37E-111
4	Calr	0.456323803	5.40E-70
4	Amotl2	0.454656849	5.42E-206
4	15-Sep	0.454570889	4.09E-68
4	Dpp4	0.454565834	0
4	Fam195b	0.452895785	6.96E-80
4	Adgra2	0.452216541	0
4	Bckdhb	0.451924731	5.54E-131
4	Rbp4	0.450711105	0
4	Cav1	0.450293268	1.13E-258
4	Sec62	0.448545551	1.63E-64
4	Zeb1	0.447409092	0
4	Sec31a	0.447341699	1.34E-119
4	Gja1	0.446851981	1.62E-75
4	Crip1	0.445489313	1.39E-46
4	Ddb1	0.445481007	2.22E-96
4	Ecscr	0.443920946	5.94E-262
4	S100a11	0.443732953	1.05E-71
4	Ramp2	0.441037953	3.54E-262
4	Pdia5	0.440044011	8.54E-263
4	Ppa1	0.438918953	1.36E-66
4	Creb3l2	0.437756715	2.20E-194
4	Sbsn	0.437374811	0
4	Bcat1	0.436681335	0
4	Serf1	0.435987415	8.46E-95
4	11-Sep	0.435364313	4.75E-86
4	Wnt5a	0.434999808	8.06E-263
4	Prrc1	0.434986874	1.26E-158
4	Hs6st2	0.434561363	0
4	Lamb2	0.434156068	6.48E-222
4	Phldb2	0.433646995	2.57E-287
4	Dkk3	0.433305054	0
4	Prrx2	0.432873434	2.01E-287
4	Bdh2	0.432554157	0
4	Gnpnat1	0.432500383	2.70E-120
4	Golim4	0.432297106	1.09E-162
4	Tpbg	0.432218944	1.57E-164
4	Rab2a	0.431749275	1.81E-71
4	Fap	0.430578576	0
4	Lix1l	0.428855315	1.76E-158
4	Ctdsp2	0.428444552	2.09E-110
4	Myadm	0.427609982	1.49E-97
4	Tspan4	0.42736708	6.49E-62
4	Itgb1	0.425751429	4.21E-54
4	Adam19	0.425330211	7.24E-225
4	Aldh7a1	0.425298328	2.33E-165
4	Yif1a	0.42461439	3.40E-93
4	Ppfibp1	0.42265895	1.95E-123
4	Sec61b	0.422630159	7.92E-54

4	Plod1	0.42065798	2.93E-73
4	Ugcg	0.420035102	4.27E-78
4	Epdr1	0.420033227	3.13E-279
4	Ssr1	0.419889131	1.64E-61
4	Sfr1	0.418691475	1.45E-59
4	Nxn	0.418074981	4.07E-161
4	Dapk1	0.417869929	4.56E-81
4	Hmgn1	0.416862437	1.13E-52
4	Rexo2	0.41674309	5.40E-61
4	Pdlim2	0.415886061	4.65E-115
4	Axl	0.4152126	2.80E-52
4	Emilin1	0.414887989	1.91E-136
4	Hspb8	0.414530518	1.23E-127
4	Armcx3	0.414338539	2.05E-213
4	Serf2	0.414226293	2.07E-84
4	AC163329.3	0.413777553	1.26E-253
4	Ifi205	0.413395022	8.64E-228
4	Cpz	0.413099528	0
4	Hoxc8	0.412487965	0
4	Ptov1	0.412088181	1.85E-83
4	Fkbp14	0.411544708	0
4	Ms4a4d	0.409044459	0
4	Sec13	0.408678727	1.23E-63
4	Bnc2	0.407977452	0
4	Cnpy2	0.405930335	7.21E-66
4	Sec61a1	0.405740823	2.93E-88
4	Bsg	0.404328783	8.72E-29
4	Dnajb1	0.404044355	2.48E-19
4	Rhoc	0.404001306	1.33E-64
4	Cercam	0.403513342	0
4	Il6	0.403502098	7.21E-69
4	Galnt16	0.402475638	0
4	Golt1b	0.400939444	2.06E-115
4	Eef1g	0.400801125	9.16E-61
4	Sec22b	0.400333134	1.86E-87
4	Fzd2	0.40005417	8.58E-254
4	Dsel	0.400014949	0
4	Plpp5	0.399746221	6.88E-141
4	Rps4l	0.399375537	0
4	Ptges	0.396467114	1.99E-141
4	Lgi2	0.396344171	0
4	Tubb2a	0.396130653	5.66E-67
4	P4hb	0.395700292	7.78E-48
4	Csf1	0.393575411	1.83E-101
4	Erlec1	0.392975111	3.59E-110
4	Rabac1	0.392060169	7.23E-62
4	Igsf3	0.388647269	1.47E-198
4	Gorasp2	0.388265169	1.97E-82
4	Bmper	0.386868803	0
4	Ebf2	0.386298206	0
4	Dbn1	0.386137459	9.66E-133
4	Mdk	0.386054251	0
4	Rpl22l1	0.385588687	1.23E-60
4	Ehd2	0.385030888	7.82E-139
4	Hcfc1r1	0.384997133	1.80E-48
4	Nckap1	0.384917962	4.57E-124
4	Hebp2	0.384615702	2.43E-144
4	Cmtm3	0.383873337	6.80E-69
4	Hsp90b1	0.383522588	9.04E-51
4	Ifi27	0.383172603	1.28E-62
4	Enah	0.381601808	6.02E-173

4	Lrrc32	0.381504251	0
4	Cd81	0.380435443	1.31E-42
4	Fkbp1a	0.380350427	1.54E-54
4	Pdlim7	0.379161722	9.63E-71
4	Ddah2	0.37836872	1.35E-87
4	Dap	0.377940113	8.72E-57
4	Sh3pxd2a	0.377275673	4.76E-141
4	Tpst1	0.377022414	8.54E-157
4	Aif1l	0.375851691	2.31E-137
4	Manf	0.375725868	2.66E-39
4	RP23-453B4.1	0.375500061	0
4	Rora	0.375045191	1.07E-250
4	Tubb6	0.373126039	1.35E-26
4	Lrrc59	0.372697448	8.25E-52
4	Steap1	0.372411872	1.58E-178
4	Col27a1	0.371522443	0
4	Dynlrb1	0.370335209	1.32E-53
4	Cnn2	0.369552471	4.92E-28
4	Mgarp	0.369298765	3.81E-131
4	Epb41l3	0.367751501	0
4	Inhba	0.367120162	1.74E-15
4	Anxa2	0.365774062	2.10E-35
4	Vim	0.364596304	5.93E-50
4	P4ha1	0.364046896	1.96E-46
4	Tubb2b	0.363712158	7.29E-175
4	Gpr180	0.363707289	3.46E-153
4	Reep5	0.361440238	3.20E-45
4	Osr2	0.360377189	2.03E-110
4	Il3ra	0.356870855	1.10E-171
4	Sec23a	0.355664931	6.51E-130
4	Arsi	0.355560666	0
4	Copb2	0.354401778	1.17E-68
4	Ggh	0.353689091	5.30E-94
4	Rnd3	0.353403167	5.75E-111
4	Pdia3	0.353321645	2.79E-44
4	Id3	0.352989789	8.68E-50
4	Actn1	0.352554273	3.01E-43
4	Tnfrsf1a	0.352166538	2.80E-75
4	Tax1bp3	0.352100779	1.87E-58
4	Kremen1	0.349614489	4.69E-187
4	Ddost	0.349465263	1.52E-43
4	Svil	0.348859402	2.04E-62
4	Ergic1	0.348816598	6.74E-94
4	Myh9	0.348567127	1.41E-46
4	Cdr2l	0.348497826	0
4	Chst12	0.348201895	1.26E-140
4	Bag3	0.348126483	9.85E-56
4	Ace	0.347058672	9.94E-56
4	RP23-204I16.3	0.346753093	0
4	Gars	0.346394777	1.87E-66
4	Me1	0.346257466	3.55E-162
4	Tmeff2	0.345965618	0
4	Pkd2	0.345477373	1.27E-97
4	Ormdl3	0.345044458	2.38E-165
4	Arf5	0.343420487	3.98E-49
4	F3	0.342667827	9.37E-138
4	Ggt5	0.342472459	4.27E-94
4	RP23-3N21.9	0.342433656	0
4	B3gnt9	0.342212477	0
4	Nucb1	0.341115368	5.38E-65
4	Mrfap1	0.340841997	9.44E-40

4	Sh3gl1	0.339176316	4.06E-94
4	Ybx3	0.33881779	1.68E-54
4	Cfh	0.338599816	1.56E-49
4	Tmem5	0.337060087	1.62E-73
4	Tram1	0.336597361	3.23E-53
4	Adamts12	0.335445048	0
4	Duoxa1	0.334764803	0
4	Rpn2	0.334529395	1.76E-44
4	Cltb	0.333819516	3.86E-69
4	Cadm3	0.333650515	0
4	Jak1	0.333532926	1.92E-60
4	Sgce	0.333381994	0
4	Tead1	0.333265622	2.87E-98
4	Tln2	0.332707652	1.71E-256
4	Pcdh19	0.332491998	0
4	Twist2	0.332409359	8.51E-280
4	RP23-159L13.1	0.332027398	0
4	Osr1	0.331989468	7.77E-222
4	Lmo4	0.331730878	2.10E-69
4	Fgf7	0.331519766	0
4	Cped1	0.331267124	3.00E-218
4	Scd2	0.331077453	6.38E-37
4	Ilk	0.331036867	7.26E-53
4	Ssc5d	0.330630227	0
4	Rras	0.330175612	4.01E-77
4	Plpp1	0.330100222	1.36E-171
4	Ldlr	0.329713772	4.70E-06
4	Nucb2	0.329106152	9.01E-72
4	Steap3	0.328683873	5.42E-169
4	Scarf2	0.328083119	2.49E-132
4	Sra1	0.327746319	3.12E-57
4	Rcn2	0.32770165	6.27E-53
4	Rab34	0.327514707	4.35E-86
4	S100a13	0.326995211	8.67E-33
4	Nudcd2	0.32631097	1.11E-61
4	Kank2	0.325955669	5.89E-144
4	Cnrip1	0.324400473	0
4	Tmem59	0.323718606	4.39E-37
4	Slit2	0.32347215	5.12E-256
4	Inhbb	0.323257586	2.06E-152
5	Hist1h2ap	1.7242	3.69E-170
5	Pclaf	1.4554	0
5	Stmn1	1.1492	3.35E-235
5	Hmgb2	1.0991	2.22E-164
5	Tuba1b	1.0685	1.07E-189
5	Birc5	0.9877	4.18E-204
5	C1qb	0.9779	4.77E-129
5	C1qc	0.9393	7.97E-123
5	Top2a	0.9308	2.30E-203
5	H2afz	0.9129	2.33E-159
5	C1qa	0.8970	7.70E-113
5	Gatm	0.8610	2.06E-131
5	Ube2c	0.8375	9.25E-157
5	Ccl12	0.8271	2.49E-73
5	Smc2	0.7935	2.16E-213
5	Ctsc	0.7789	3.66E-110
5	Msr1	0.7442	4.05E-110
5	Dek	0.7148	2.30E-121
5	Fcrls	0.7089	7.40E-106
5	Mki67	0.7074	3.44E-240
5	Ifi30	0.7071	3.27E-105

5	Ran	0.6918	1.28E-120
5	Aif1	0.6911	6.25E-96
5	Cenpa	0.6776	9.93E-101
5	Nusap1	0.6715	1.01E-212
5	Cdca3	0.6409	1.90E-218
5	Lyz2	0.6385	1.08E-71
5	Cx3cr1	0.6383	1.64E-134
5	Rrm2	0.6341	5.15E-164
5	Ccna2	0.6236	7.05E-199
5	Pcna	0.6232	3.78E-84
5	RP23-387F9.3	0.6157	2.29E-101
5	H2afx	0.6153	1.46E-103
5	Rrm1	0.6104	1.82E-194
5	Cdca8	0.6085	3.48E-183
5	Ptgs1	0.6067	1.22E-125
5	Cotl1	0.5988	1.31E-86
5	Ntpcr	0.5944	2.71E-111
5	Hmgb1	0.5862	5.96E-89
5	Tmpo	0.5862	4.42E-107
5	Bcl2a1a	0.582870342	9.42E-116
5	Bcl2a1b	0.582093832	1.02E-82
5	Ranbp1	0.576153339	6.15E-81
5	Cox5a	0.574383134	4.30E-101
5	Smc4	0.571787621	4.18E-101
5	Cks1b	0.570884119	9.76E-107
5	Cxcl16	0.57036164	7.13E-86
5	Kpna2	0.568824868	3.25E-101
5	Racgap1	0.567967083	1.21E-178
5	Ly86	0.563302466	1.84E-95
5	Nucks1	0.560329519	1.88E-88
5	Tmsb4x	0.559773409	3.20E-97
5	Pttg1	0.559055029	1.64E-110
5	Mgl2	0.556491432	8.75E-94
5	Ms4a6c	0.553806954	9.50E-75
5	Serinc3	0.545347581	6.37E-80
5	Cdk1	0.539652736	5.11E-116
5	Ms4a6b	0.539593446	1.02E-84
5	Hnrnpab	0.532801595	1.06E-76
5	Ctss	0.528159606	3.37E-62
5	Ybx1	0.521297339	1.20E-95
5	Gmnn	0.519949383	2.89E-131
5	Tubb5	0.51834594	1.45E-79
5	Ube2s	0.517806008	1.72E-68
5	Hmgn2	0.514284615	2.99E-76
5	Bcl2a1d	0.512836108	5.51E-95
5	Mcm3	0.51106323	1.07E-128
5	Fam96a	0.510773999	1.57E-76
5	Clec4n	0.509899533	1.96E-70
5	Hexb	0.508818296	4.84E-79
5	Fam111a	0.508211966	5.30E-149
5	Anp32b	0.506774557	3.84E-75
5	Ctsz	0.505511503	3.13E-72
5	Sdf2l1	0.505203196	1.05E-77
5	Slc25a5	0.504726738	1.38E-73
5	Asf1b	0.502752027	7.11E-207
5	Arl6ip1	0.501817742	1.12E-50
5	Tpx2	0.494004272	6.30E-164
5	Erp29	0.493938488	4.02E-81
5	Anp32e	0.489893379	5.95E-77
5	Tacc3	0.487290437	7.00E-160
5	Lsm5	0.485326806	7.84E-76

5	Apoe	0.485269323	3.61E-64
5	Lig1	0.483889514	1.05E-112
5	Syce2	0.48227753	2.85E-207
5	Ptma	0.481536645	1.49E-97
5	Spc24	0.481337409	1.70E-146
5	Cks2	0.479687319	1.12E-73
5	Smdt1	0.476591041	1.16E-72
5	Mcm5	0.475222809	9.44E-120
5	Spc25	0.47284319	4.30E-169
5	Rgs10	0.470619311	4.03E-75
5	Pld4	0.468530386	1.76E-89
5	Tyms	0.467655717	9.14E-100
5	Stra13	0.466392934	6.79E-80
5	Arhgdib	0.462177597	1.59E-68
5	Tk1	0.459923259	1.28E-133
5	Tmem14c	0.457663955	2.22E-66
5	Dnajc9	0.456592302	3.73E-114
5	Pmf1	0.455171183	8.61E-104
5	Dctpp1	0.454563363	7.10E-86
5	Snx2	0.453027411	9.03E-74
5	Fcgr1	0.448862224	2.87E-89
5	H2afy	0.447405378	5.54E-66
5	RP23-381B19.2	0.443833543	1.56E-88
5	Trf	0.442962967	3.56E-46
5	Gusb	0.442269976	7.98E-77
5	Csf1r	0.438998526	1.87E-64
5	Cmc2	0.434611915	3.12E-96
5	Ccnb2	0.430700241	2.04E-87
5	Incenp	0.429867836	1.40E-141
5	Hpgds	0.429845452	1.30E-117
5	Cfp	0.429245886	9.86E-69
5	Ccnb1	0.428099866	3.67E-130
5	Ccdc34	0.42563381	4.65E-78
5	Lepr	0.423331674	1.95E-34
5	Mcm6	0.420755903	1.10E-77
5	Fam105a	0.420549616	6.09E-88
5	Aurkb	0.419395647	1.22E-132
5	Eif1ad	0.417353007	1.28E-105
5	Hnrnpa3	0.416614224	4.75E-57
5	Unc93b1	0.415361261	1.50E-66
5	Cenpw	0.414344187	3.13E-107
5	Pycard	0.414291841	2.02E-69
5	Snrpb	0.4130417	5.37E-52
5	Dck	0.41079539	4.49E-91
5	Atp5g3	0.410733356	1.41E-53
5	Fen1	0.410544788	2.67E-147
5	Alyref	0.409643828	7.45E-65
5	RP24-176A1.3	0.409219335	2.02E-74
5	Pbk	0.409173293	5.63E-107
5	Rad21	0.408935914	4.70E-51
5	Set	0.408068636	2.44E-50
5	Prc1	0.406680944	2.66E-116
5	Slamf9	0.406541759	4.72E-128
5	Grn	0.406174323	4.18E-52
5	Cycs	0.405978378	5.67E-47
5	Cenpe	0.405332209	2.05E-125
5	Adamdec1	0.40410436	1.07E-53
5	Clec4a2	0.403924531	6.73E-81
5	Lgmn	0.402608066	6.07E-52
5	Atp5a1	0.401583459	1.81E-50
5	Ncf4	0.400585691	1.80E-57

5	Dtymk	0.399316362	5.90E-66
5	Tkt	0.398474267	1.37E-53
5	Kif11	0.397788674	8.65E-162
5	Mcm7	0.39669456	3.70E-85
5	Rnaseh2c	0.395595384	7.02E-70
5	Hn1	0.395347954	6.50E-44
5	Mis18a	0.39517464	1.29E-84
5	Lsm2	0.394082745	7.10E-77
5	Uhrf1	0.393050514	9.55E-102
5	Cd72	0.391107024	3.34E-87
5	Ndufb8	0.387123034	8.36E-52
5	Mrpl51	0.38473922	8.75E-60
5	Tubb4b	0.382830975	9.65E-50
5	H2-DMa	0.381624549	7.76E-58
5	Hexa	0.380251566	1.28E-51
5	Mad2l1	0.380056063	3.42E-127
5	Cenpf	0.380001377	9.12E-78
5	Pa2g4	0.378954489	1.55E-46
5	Hat1	0.377179401	1.28E-79
5	Adgre1	0.376835366	2.27E-70
5	Cfl1	0.376663858	2.95E-70
5	Naaa	0.374196724	7.38E-61
5	Ms4a6d	0.366951292	6.72E-51
5	Cybb	0.366473494	3.92E-63
5	Ptpn6	0.365567214	4.96E-69
5	Psmb9	0.365556751	1.91E-46
5	Hnrnpf	0.363144788	5.06E-44
5	Ezh2	0.362708981	1.30E-76
5	Atad2	0.361975666	1.06E-63
5	Sae1	0.361074152	7.18E-64
5	Ucp2	0.360871776	3.27E-44
5	Rfc5	0.360357601	6.48E-110
5	Cd81	0.358734515	3.39E-32
5	Mrpl18	0.356975757	7.70E-48
5	Hprt	0.354369523	4.48E-52
5	Hcls1	0.354056125	8.45E-69
5	Arrb2	0.352668403	9.12E-68
5	Bola2	0.352206942	3.81E-42
5	Hnrnpa2b1	0.352132088	2.41E-45
5	Tuba1c	0.350540515	7.82E-41
5	Hist1h2ae	0.349831533	3.55E-81
5	Uqcr10	0.349435103	5.10E-53
5	Siva1	0.349178584	3.11E-41
5	Rangap1	0.344705375	3.89E-71
5	Atp5o	0.343313732	1.33E-43
5	Usp1	0.342987193	8.19E-69
5	Actb	0.341888178	5.89E-38
5	Rnase2a	0.341051888	1.58E-38
5	Cbx3	0.338734971	8.50E-43
5	Tmco1	0.338324617	9.95E-43
5	Rpn1	0.33832	2.27E-44
5	Rfc2	0.337981856	4.54E-66
5	Dnmt1	0.337967945	3.31E-74
5	Tipin	0.337931553	8.66E-53
5	AC114920.2	0.33758189	5.07E-51
5	Serbp1	0.336986831	2.61E-37
5	Snrpd1	0.336194808	3.11E-45
5	Knstrn	0.335594761	2.24E-84
5	Nhp2	0.334760013	1.02E-41
5	Rgs1	0.334706719	8.50E-51
5	Sdhd	0.334508148	2.45E-41

5	Atp1b3	0.333729864	7.47E-34
5	Cenpm	0.333666656	1.29E-152
5	Bub1b	0.333168819	1.94E-124
5	Fkbp2	0.331417992	1.52E-41
5	Cenph	0.330824163	1.40E-106
5	Cyba	0.330523066	8.90E-36
5	Tagln2	0.329805374	6.47E-39
5	Mrpl28	0.329671893	8.09E-49
5	Xist	0.32957429	1.83E-31
5	Ndc1	0.329343285	1.78E-95
5	Coro1a	0.329206819	1.22E-43
5	Ptpn7	0.326779761	4.51E-114
5	Mef2c	0.323515634	5.86E-69
5	RP24-482A24.5	0.323506606	4.98E-35
5	Apitd1	0.323448086	4.94E-88
6	Krt8	1.5465	1.02E-193
6	Hist1h2ap	1.4992	6.35E-221
6	Krt18	1.4990	2.03E-174
6	Ccnd1	1.4176	1.96E-202
6	Cks1b	1.3364	1.91E-227
6	Hmgb2	1.3332	1.43E-170
6	Top2a	1.3165	5.85E-291
6	Tubb5	1.2389	9.34E-157
6	Lmna	1.2174	1.05E-163
6	Lgals1	1.1933	2.65E-175
6	S100a6	1.1861	8.90E-144
6	Ube2c	1.1655	8.87E-229
6	Mtap	1.1481	2.22E-227
6	Vim	1.1363	3.72E-140
6	Birc5	1.1247	2.26E-259
6	Tubb4b	1.1015	8.18E-177
6	RP24-127H11.10	1.0920	7.86E-222
6	Cdk1	1.0801	3.73E-259
6	Wfdc2	1.0750	1.24E-161
6	Stmn1	1.0619	1.39E-176
6	Phgdh	1.0344	0
6	Tnfrsf12a	1.0165	5.16E-171
6	Smc4	1.0144	1.85E-218
6	Tm4sf1	1.0034	4.33E-176
6	H2afz	1.0013	3.87E-149
6	Cks2	0.9913	8.54E-183
6	Hmgn1	0.9868	1.18E-156
6	Cenpf	0.9657	0
6	Cenpa	0.9503	1.80E-154
6	Ube2s	0.9499	1.84E-137
6	Cdc20	0.9458	0
6	H2afv	0.9448	4.11E-143
6	Hmgb1	0.9387	2.31E-145
6	Tuba1b	0.9384	6.08E-129
6	Npm1	0.9238	1.24E-148
6	Oaf	0.9182	1.12E-176
6	S100a10	0.9149	1.17E-130
6	Pclaf	0.9087	2.30E-224
6	S100a4	0.9071	1.23E-109
6	Tk1	0.9059	0
6	Dut	0.9005	1.01E-163
6	H1f0	0.8879	3.06E-107
6	Prkg2	0.8773	2.06E-280
6	Pttg1	0.8742	3.64E-212
6	Impdh2	0.8705	8.15E-148
6	Psip1	0.8654	3.39E-233

6	Ercc1	0.8490	5.42E-185
6	Fkbp3	0.8414	8.29E-155
6	Nop58	0.8381	3.98E-171
6	Spint2	0.8380	4.98E-170
6	Hmga1-rs1	0.8227	1.00E-291
6	Ccnb2	0.8179	6.41E-290
6	Ptma	0.8173	9.61E-154
6	Ncl	0.8138	3.50E-105
6	Ndufb9	0.8134	4.33E-128
6	Hmgn2	0.8107	8.98E-134
6	Ppia	0.8063	3.30E-164
6	Krt7	0.7972	1.92E-203
6	Ran	0.7965	3.72E-120
6	Odc1	0.7894	1.15E-117
6	Slc25a4	0.7894	3.71E-112
6	Ranbp1	0.7874	7.83E-113
6	Eif5a	0.7851	8.49E-105
6	Tbrg1	0.7833	1.36E-132
6	Lyar	0.7827	5.55E-163
6	Cdk4	0.7814	2.01E-124
6	Epcam	0.7676	1.15E-195
6	Prdx2	0.7652	1.24E-118
6	Pebp1	0.7636	8.65E-116
6	Cd9	0.7600	2.85E-92
6	Hspa8	0.7384	7.85E-86
6	Rrm2	0.7336	1.23E-260
6	Htra1	0.7302	1.33E-181
6	Hmga2	0.7246	3.09E-238
6	Rps2	0.7158	1.33E-122
6	Ccdc34	0.7090	1.81E-170
6	Smc2	0.7089	5.74E-265
6	Phlda1	0.7060	5.49E-65
6	Clic1	0.7048	1.34E-101
6	Hsp90ab1	0.7012	2.99E-114
6	Erh	0.6993	1.50E-108
6	Ccna2	0.6938	0
6	Pabpc1	0.6904	6.05E-104
6	Anln	0.6901	0
6	Calm2	0.6878	3.97E-99
6	Cenpe	0.6854	0
6	Prmt1	0.6836	2.54E-129
6	Set	0.6829	1.57E-93
6	Igfbp4	0.6799	3.24E-106
6	Dtymk	0.6785	1.46E-129
6	Nap1l1	0.6783	3.49E-109
6	H2afx	0.6780	2.45E-184
6	Cct5	0.6771	2.38E-104
6	Tuba1a	0.6737	1.11E-109
6	RP23-387F9.3	0.6726	3.34E-116
6	Bok	0.6691	1.57E-303
6	Lsm2	0.6688	1.09E-154
6	Spc24	0.6679	0
6	Anxa5	0.6648	5.75E-89
6	Sfn	0.6642	0
6	Hdgf	0.6634	1.61E-108
6	Eno3	0.6623	9.26E-257
6	Plp2	0.6619	9.89E-132
6	Anp32b	0.6587	5.45E-98
6	Atp5b	0.6580	6.95E-102
6	Lockd	0.6554	6.04E-256
6	Hnrnpa1	0.6525	1.99E-101

6	RP23-336M12.2	0.6517	1.47E-112
6	Prc1	0.6509	0
6	Itga3	0.6502	6.63E-248
6	Ppa1	0.6485	3.96E-147
6	Tsen34	0.6477	1.38E-145
6	Tinagl1	0.6476	8.04E-246
6	AC164564.3	0.6459	1.86E-179
6	Plet1	0.6457	5.82E-253
6	Rplp0	0.6422	1.90E-123
6	Ywhaq	0.6361	1.20E-94
6	Hint1	0.6327	7.73E-118
6	Hspd1	0.6314	7.49E-79
6	Tmpo	0.6311	1.81E-144
6	Kcnn4	0.6304	3.91E-249
6	Cep57	0.6284	1.96E-220
6	Kate2	0.6279	1.23E-162
6	Ddah2	0.6267	8.52E-157
6	Sumo2	0.6219	4.65E-95
6	Srsf3	0.6195	2.49E-89
6	Dctpp1	0.6182	1.60E-115
6	Maged1	0.6162	4.31E-110
6	Dcps	0.6158	2.39E-168
6	Nhp2	0.6147	2.49E-80
6	Ccnb1	0.6081	2.67E-295
6	Kpna2	0.6076	2.24E-168
6	Tipin	0.6074	8.33E-171
6	Pbk	0.6057	0
6	Cct4	0.6045	4.38E-88
6	Map1b	0.6045	1.29E-195
6	Nusap1	0.6039	6.70E-287
6	Cpe	0.6026	9.59E-214
6	Dpy30	0.6005	6.69E-123
6	Cbx3	0.5971	2.06E-83
6	Snrpf	0.5961	3.93E-96
6	Tyms	0.5942	1.38E-209
6	Eif3l	0.5914	2.69E-111
6	Hmgb3	0.5905	1.04E-264
6	Prdx4	0.5892	6.34E-96
6	Mrpl13	0.5877	4.23E-126
6	Dek	0.5860	3.14E-101
6	Cct2	0.584473823	1.82E-94
6	Serbp1	0.582342344	1.35E-89
6	Cdca8	0.58151005	1.75E-302
6	Hsp90aa1	0.581241477	9.49E-68
6	Ndufa4	0.576355756	3.13E-82
6	Usp50	0.57474993	5.93E-73
6	Tuba1c	0.573730133	2.87E-86
6	Cct3	0.573692598	1.22E-90
6	Ddx39	0.573270621	1.26E-130
6	Ubald2	0.571370663	8.08E-70
6	Tpm1	0.57128346	7.92E-134
6	Col18a1	0.569537124	8.58E-254
6	H19	0.565196337	1.54E-122
6	Rhoc	0.564559035	3.04E-86
6	Tnnt2	0.564036568	3.25E-147
6	Eif3e	0.561182471	3.95E-76
6	Vdac3	0.560440071	6.41E-91
6	Cd24a	0.558394335	7.73E-119
6	Mcm6	0.558336896	1.36E-198
6	Cd151	0.556278317	2.13E-156
6	Cenpw	0.555515138	7.89E-248

6	Id3	0.553871433	4.25E-78
6	Dynll1	0.552805393	9.59E-56
6	Eny2	0.552557039	2.09E-91
6	Hmmr	0.551758043	0
6	Vars	0.550294414	7.44E-176
6	Tcp1	0.548012045	2.06E-89
6	Gpx4	0.547996358	1.85E-87
6	U2af1	0.546298848	6.67E-93
6	Sms	0.546283396	2.93E-175
6	Anp32e	0.543878448	8.87E-100
6	Sox9	0.543347656	2.18E-201
6	Glo1	0.542085431	7.18E-102
6	Slbp	0.541656685	2.19E-97
6	Paics	0.540004041	5.66E-107
6	Tacc3	0.538697952	3.03E-298
6	Txn1	0.538608408	4.63E-88
6	Pa2g4	0.536224042	8.93E-94
6	Cox6c	0.533909875	7.94E-99
6	Tmem238	0.5329301	7.84E-168
6	Ank	0.532347449	2.41E-163
6	Cnn3	0.531489091	7.09E-132
6	Cald1	0.530517828	6.20E-110
6	Slco2a1	0.530196536	4.43E-186
6	Tspan4	0.530000832	6.96E-101
6	Cnih4	0.529429488	8.69E-79
6	G3bp1	0.529293862	2.35E-88
6	Nucks1	0.529249024	6.33E-91
6	Hells	0.528750175	2.87E-252
6	Banf1	0.528206942	1.65E-85
6	Fkbp4	0.527680264	2.37E-104
6	Tomm22	0.524807445	3.22E-83
6	Dap	0.523825081	5.05E-83
6	Myl12a	0.522502937	6.85E-75
6	Anxa2	0.521557586	5.77E-66
6	Cttn	0.520706053	1.93E-147
6	Alyref	0.51998777	3.68E-100
6	Nop56	0.519700833	9.48E-110
6	Rpl6	0.519377927	3.15E-101
6	Plpp2	0.519049669	7.48E-195
6	Snrpg	0.518809316	1.58E-86
6	Nme2	0.518718823	3.00E-68
6	Ubb	0.517693293	9.11E-52
6	Ywhae	0.517079953	6.05E-80
6	Nudt21	0.516364724	2.31E-90
6	Nme1	0.516287639	2.36E-54
6	Hnrnpa3	0.514462427	1.93E-76
6	RP23-310J6.1	0.512506482	3.50E-154
6	Ppid	0.511553416	3.47E-128
6	Tpx2	0.510531098	4.09E-299
6	Fosl1	0.510311176	1.27E-220
6	Psmd7	0.50904876	1.43E-82
6	Hmgn3	0.506374129	9.88E-208
6	Polr2f	0.506217718	2.43E-74
6	Nol7	0.505686201	2.93E-94
6	Nfix	0.505623342	7.46E-137
6	Gnas	0.505576101	4.62E-64
6	Pcna	0.505340133	5.57E-73
6	Atad2	0.501876881	4.46E-177
6	Cct7	0.501717799	2.36E-76
6	Rpsa	0.500657398	1.91E-107
6	Ppp1r14b	0.500503234	1.46E-62

6	Ngfrap1	0.498766704	3.69E-107
6	Casp3	0.498408959	1.10E-152
6	Tspan3	0.498360983	9.57E-98
6	Psmb7	0.49771501	1.87E-75
6	Snrpe	0.495845171	9.99E-72
6	Rcn2	0.494779252	3.05E-91
6	Spata7	0.494295329	4.99E-119
6	Eif4a1	0.4939151	9.96E-75
6	Gmnn	0.493911422	8.65E-179
6	Mki67	0.493705321	7.30E-250
6	Ilf2	0.493316815	7.43E-147
6	Ppil1	0.492870649	1.38E-187
6	Gcat	0.49046077	5.86E-229
6	Ndufa12	0.489780546	5.45E-73
6	Sfr1	0.489553206	1.59E-76
6	H3f3b	0.488744194	5.45E-69
6	Rbbp7	0.487187572	9.54E-109
6	Psma5	0.486942457	1.74E-71
6	Exosc8	0.486348624	1.36E-157
6	Cct6a	0.486264817	1.28E-76
6	Gm26825	0.485897313	7.39E-83
6	Polr2e	0.483038209	4.55E-87
6	Ankrd1	0.481293803	8.68E-196
6	Racgap1	0.479079085	1.60E-302
6	Hnrnpab	0.479058697	7.67E-68
6	Atp5c1	0.477101626	9.46E-67
6	Cenpv	0.474218801	1.64E-268
6	Nup37	0.472463035	5.33E-260
6	Bub3	0.471570083	2.79E-81
6	Knstrn	0.470663668	2.91E-268
6	Zfp706	0.470262733	7.66E-71
6	Wnt7b	0.468227974	6.74E-238
6	Snrpd2	0.468170504	8.30E-69
6	Rtn4	0.46788116	5.00E-65
6	Nfib	0.466834558	1.70E-151
6	Nasp	0.466801745	9.90E-136
6	Dnajb1	0.466514248	2.43E-20
6	Snrpd3	0.466163714	4.83E-69
6	Sox4	0.465744624	3.64E-106
6	Tsc22d1	0.464538354	3.81E-96
6	Hn1	0.46430243	8.48E-52
6	Mrps6	0.463682323	2.98E-142
6	Hdac2	0.463210629	5.58E-109
6	Nedd4	0.460491532	7.63E-109
6	Tmsb10	0.460151099	8.17E-56
6	Pdap1	0.459560667	1.71E-77
6	Pin1	0.459398552	5.39E-101
6	Rpa3	0.457826747	3.20E-112
6	Snrpd1	0.456853482	3.04E-73
6	Cbx1	0.456673484	1.69E-105
6	Amotl1	0.455129934	4.84E-165
6	Sgol1	0.454772138	0
6	Adss	0.453776043	1.27E-122
6	Npnt	0.453475064	1.32E-205
6	Prss22	0.452882065	2.07E-206
6	Sae1	0.452195613	5.80E-139
6	Anapc5	0.451793893	8.58E-88
6	Trim28	0.451280676	9.76E-108
6	Rpl5	0.450609871	1.85E-63
6	Rpl14	0.449483374	5.16E-90
6	Ezr	0.448470136	6.04E-83

6	Cd82	0.448312601	1.43E-147
6	Med24	0.447083755	2.18E-164
6	Steap2	0.446687401	2.76E-247
6	S100a11	0.445921435	3.85E-52
6	Lsm4	0.445245533	1.46E-63
6	Tagln2	0.44424422	2.77E-50
6	Nudc	0.443374482	3.19E-75
6	Rad21	0.442688162	2.11E-99
6	Mast4	0.442539946	1.32E-177
6	Ak2	0.440948355	8.79E-67
6	Lig1	0.440379243	8.56E-148
6	C1qbp	0.439479993	1.35E-67
6	Wwtr1	0.437487788	1.46E-165
6	Lad1	0.436801222	6.16E-260
6	Rpl30	0.435716139	1.34E-72
6	Hoxa5	0.435416349	3.31E-250
6	Ncapg	0.435098893	0
6	Ssrp1	0.434357452	2.83E-94
6	Psma6	0.434267421	2.77E-61
6	Mcm7	0.43367158	3.24E-152
6	Tomm20	0.43338501	8.53E-59
6	Ndufaf2	0.43268274	1.15E-131
6	Rpl15	0.432500531	1.72E-62
6	Prkcdbp	0.431275986	3.90E-124
6	Mgst3	0.431077363	3.45E-124
6	RP23-418M3.5	0.430578804	7.01E-142
6	Kif23	0.430113167	0
6	Srsf7	0.429501834	4.70E-66
6	RP24-78F23.1	0.429292068	0
6	Lsm5	0.429180276	6.26E-68
6	Ybx3	0.428719357	2.08E-80
6	Pola2	0.427905015	1.12E-74
6	Hjurp	0.427898452	6.64E-179
6	Naca	0.425136994	9.28E-72
6	Gata3	0.424721387	2.17E-227
6	Psmc2	0.423314724	7.97E-95
6	Rps10	0.423295564	3.73E-80
6	Zwint	0.422356099	4.49E-108
6	Plk1	0.421932134	0
6	Galk1	0.4219103	6.67E-96
6	Rpl4	0.420905939	1.08E-61
6	Pmf1	0.420654468	2.03E-144
6	Brix1	0.42060545	1.45E-133
6	Rdx	0.420400111	2.79E-78
6	Cfdp1	0.420306503	1.84E-79
6	Rpl7	0.41998072	2.93E-75
6	Sarnp	0.419091931	1.03E-66
6	Ube2n	0.418906224	6.80E-67
6	Ppa2	0.418422616	3.12E-148
6	Mrpl42	0.418259207	1.37E-66
6	Ssb	0.417297245	1.65E-61
6	Pafah1b3	0.416083134	2.09E-102
6	Commd1	0.416063323	8.38E-77
6	Gar1	0.414799734	6.58E-122
6	Actn1	0.41461594	2.33E-118
6	Aurkb	0.412799891	2.02E-254
6	Apex1	0.412472972	2.28E-96
6	Acot7	0.412067466	1.39E-141
6	Cnn2	0.411965653	1.02E-72
6	Metap2	0.411903619	1.02E-57
6	Lsm7	0.411648297	7.97E-64

6	Prdx1	0.410918783	5.30E-58
6	Ruvbl1	0.409196176	4.79E-127
6	Uqcrc1	0.409103528	3.80E-57
6	Abhd2	0.409081079	5.96E-87
6	Mcm5	0.408967101	9.40E-181
6	Fscn1	0.408821475	3.82E-172
6	Psat1	0.407930104	4.02E-179
6	Usp1	0.40767745	2.55E-149
6	Snrpb	0.406772863	6.59E-55
6	Plk4	0.406120115	3.35E-286
6	Lsm8	0.405221665	4.36E-101
6	Srpr	0.405151573	6.68E-103
6	Ier2	0.403926374	2.98E-27
6	Lamb1	0.40390318	1.02E-136
6	Padi4	0.403605183	2.44E-181
6	Magoh	0.402840931	7.38E-76
6	Plec	0.402480756	3.78E-105
6	Nenf	0.402306429	2.68E-56
6	Rbmxl1	0.401969199	1.22E-119
6	Sdc2	0.400947598	1.96E-125
6	Nudt4	0.400128589	5.54E-83
6	Orc6	0.399763502	2.26E-180
6	Pold2	0.398465467	4.35E-219
6	Eif3g	0.398189056	1.08E-66
6	Mrpl12	0.397489602	1.05E-62
6	Mad2l1	0.396901053	1.10E-255
6	Prdx6	0.395621978	3.69E-54
6	AC114920.2	0.39549909	4.96E-91
6	Kank3	0.394729212	1.04E-202
6	Ddx39b	0.394451487	3.24E-65
6	Gtf2a2	0.394405537	1.39E-84
6	Rnps1	0.394383531	7.96E-98
6	Psmd12	0.394249409	8.88E-99
6	Cnbp	0.393681597	1.56E-47
6	Trim59	0.393644983	7.72E-221
6	Fam64a	0.39334987	0
6	Hddc2	0.393128899	2.37E-132
6	Cdkn2c	0.391998975	5.91E-111
6	Psmb3	0.391901974	9.31E-46
6	15-Sep	0.391785622	8.77E-47
6	Aimp1	0.39042616	2.75E-70
6	Cd63	0.390306879	2.75E-38
6	Sigirr	0.3899854	3.28E-294
6	Fbl	0.389960466	3.32E-59
6	Cacybp	0.389693823	3.05E-49
6	Pi4k2b	0.38966488	4.41E-117
6	Dst	0.389626407	1.45E-131
6	Spc25	0.389433539	4.43E-226
6	Ruvbl2	0.388302059	1.23E-152
6	Hnrnpd	0.388286281	3.21E-81
6	Mtmr2	0.387957882	4.03E-154
6	Anapc11	0.387036041	7.22E-58
6	Rbm3	0.386808438	5.76E-53
6	Krtcap3	0.385831344	2.36E-222
6	Rsl1d1	0.38556495	1.21E-85
6	Jun	0.385119802	2.83E-27
6	Rpl3	0.383777623	3.55E-54
6	RP23-463F7.8	0.383505092	5.33E-72
6	Lsm3	0.382918736	2.28E-103
6	Shmt2	0.382686039	1.03E-126
6	H2-Q4	0.38142445	1.85E-87

6	Anxa1	0.380488756	1.95E-49
6	Skp1a	0.380335366	1.10E-64
6	Las1l	0.380264254	8.20E-150
6	Kif22	0.380133479	1.93E-291
6	Uqcc2	0.380051196	8.68E-48
6	Rps12	0.379986033	1.46E-71
6	Eef2	0.379432629	3.65E-52
6	Rps2-ps10	0.379431851	1.01E-66
6	Arl6ip1	0.378708658	3.50E-18
6	Msln	0.378104872	9.94E-272
6	Rab34	0.377668109	2.22E-141
6	Cfl2	0.377371657	2.78E-115
6	Eno1	0.376918918	9.09E-34
6	Cdca3	0.376649002	2.08E-240
6	Rpl12	0.376597292	3.44E-57
6	Eef1g	0.376593734	4.07E-41
6	Phb2	0.37569565	4.70E-52
6	Tcf3	0.375646417	8.35E-139
6	Hbegf	0.374063176	4.72E-80
6	Rala	0.373917191	9.17E-67
6	Eif1ax	0.372606753	1.07E-62
6	Mrpl18	0.372600561	9.75E-60
6	Psma7	0.372425608	4.75E-44
6	Slc29a1	0.372354209	2.28E-96
6	Ift43	0.371845754	3.89E-139
6	Mrpl38	0.370720964	5.63E-112
6	Mtdh	0.37060264	9.21E-45
6	Itga6	0.3702762	5.01E-170
6	Larp7	0.369955229	3.07E-152
6	Lsr	0.369712588	2.17E-205
6	Uchl5	0.369598106	9.50E-115
6	Srm	0.369491805	5.06E-67
6	Uox	0.368980126	1.42E-257
6	Hnrnpa2b1	0.368532454	6.05E-49
6	Rbm8a	0.36847514	2.70E-50
6	Tmem128	0.368239883	8.04E-101
6	Dbf4	0.367986434	3.50E-215
6	Klf5	0.36762252	2.26E-208
6	Psmd1	0.367615913	6.13E-87
6	Psma3	0.367561416	3.45E-44
6	Bcas2	0.367287836	1.92E-60
6	Fam216a	0.366893335	1.37E-235
6	Btf3	0.366828442	5.99E-55
6	Vdac1	0.366682971	2.42E-55
6	Perp	0.366301621	6.90E-208
6	Taf1d	0.365756311	2.89E-74
6	Eif2s1	0.365302793	1.35E-76
6	Gja1	0.364764956	5.38E-89
6	Ktn1	0.363892171	2.20E-101
6	Nudcd2	0.363276863	1.63E-80
6	Pcmt1	0.361870963	3.91E-85
6	Snrpc	0.361269179	4.79E-61
6	Psmb4	0.36124428	8.18E-48
6	Mdh1	0.360979619	3.66E-57
6	Rfc3	0.360612306	2.34E-175
6	Klhdc2	0.360436629	9.81E-112
6	Ube2i	0.35987594	9.31E-51
6	Rfc5	0.359501552	9.67E-201
6	Fam102b	0.358732096	5.99E-111
6	Ppih	0.358577081	1.45E-126
6	Acp1	0.358244745	1.03E-91

6	Cenph	0.357329362	1.25E-298
6	Rbp2	0.357096958	1.15E-243
6	Eif3i	0.357031157	3.44E-44
6	Apoo	0.356973571	8.10E-175
6	Ywhah	0.356470986	1.23E-43
6	Exosc7	0.35591911	2.73E-135
6	Idh2	0.355610173	1.56E-72
6	Sgol2a	0.355520975	0
6	Ywhaz	0.355210507	1.73E-49
6	Ltbp1	0.355044953	4.90E-167
6	Wdr43	0.354907125	5.76E-115
6	Serpinb6a	0.354792394	6.48E-62
6	Kars	0.354625869	9.22E-115
6	Mpzl1	0.354181758	1.35E-207
6	Ezh2	0.353761	8.49E-176
6	Angptl2	0.353758241	6.80E-170
6	Psmb2	0.353551301	8.28E-42
6	Cnpy2	0.353488811	2.12E-50
6	Myef2	0.353439773	1.12E-151
6	Actl6a	0.35312674	2.76E-120
6	Ift172	0.352767596	1.05E-170
6	Snrpa1	0.352249884	2.32E-76
6	Glrx5	0.352177013	4.98E-51
6	Srpk1	0.352143072	2.34E-121
6	Hnrnph1	0.351241548	1.27E-57
6	Nsmce4a	0.351105757	3.41E-114
6	Eef1d	0.35094021	1.42E-43
6	Baiap2	0.350568714	2.27E-82
6	Atp5g1	0.349924047	2.74E-34
6	Ppib	0.348857598	2.73E-38
6	Kpnb1	0.348712804	2.58E-95
6	Rpl28	0.348707432	1.79E-62
6	Cdkn3	0.348690543	3.53E-272
6	Ect2	0.348490343	0
6	Hspe1	0.348292931	5.05E-32
6	Ppm1g	0.347704504	1.48E-100
6	Gm8186	0.346578989	2.25E-72
6	Dynlt1f	0.346448078	2.15E-122
6	Sf3b4	0.345892581	8.08E-116
6	Aurka	0.34558194	1.07E-221
6	Ptges3	0.343917615	4.76E-51
6	Dkc1	0.343627794	2.71E-173
6	Kif15	0.343618353	0
6	Nono	0.343453732	2.02E-54
6	RP23-9L16.5	0.343255598	2.19E-102
6	Psma4	0.341386529	1.82E-38
6	Nudt9	0.341134956	7.80E-67
6	Ifi27	0.34102548	1.42E-39
6	Fdps	0.341018854	9.82E-68
6	Emp2	0.340134676	4.36E-187
6	Eif3h	0.339650681	3.15E-39
6	Mif	0.339403758	6.51E-32
6	Snu13	0.33848356	6.45E-42
6	Gars	0.337505222	1.12E-93
6	Rpl31	0.336886223	2.01E-55
6	Aprt	0.336128228	1.82E-35
6	Sumo1	0.335853214	6.80E-45
6	Gins2	0.335784033	7.58E-173
6	Siva1	0.335312589	1.57E-51
6	Ssr2	0.334543959	3.71E-44
6	Asap2	0.334445434	3.25E-196

6	Slc25a3	0.334134232	8.02E-46
6	Tmprss11e	0.332986118	0
6	Ebna1bp2	0.332926189	3.48E-67
6	Asns	0.332926031	5.47E-144
6	Gnb2l1	0.332484577	1.16E-51
6	Pcolce2	0.33227998	1.03E-205
6	Srsf11	0.33218157	1.11E-54
6	Rrm1	0.332170271	1.95E-154
6	Reep4	0.332068282	2.19E-164
6	Mbd3	0.331442751	8.56E-76
6	Chrac1	0.331108817	1.80E-89
6	Prim1	0.330480345	1.12E-237
6	Smc3	0.330394802	1.44E-91
6	Nae1	0.330289272	1.37E-141
6	Serinc2	0.329780764	3.92E-215
6	Cit	0.329467267	8.40E-300
6	Tmem237	0.329156247	1.95E-182
6	Dnajc9	0.328886993	9.56E-140
6	Tfdp1	0.328738078	3.37E-106
6	Ckap2	0.328618979	2.67E-298
6	Dazap1	0.328506687	6.85E-83
6	10-Sep	0.328269439	2.00E-176
6	Fah	0.328040222	6.28E-156
6	Cct8	0.326995363	6.32E-42
6	Ola1	0.326725165	1.69E-74
6	Tecr	0.326640686	1.10E-51
6	Polr2c	0.326552749	5.80E-73
6	Ndufv2	0.326470569	1.41E-45
6	Phb	0.325087569	1.15E-91
6	Fhl2	0.325044978	9.02E-153
6	RP23-251I6.1	0.324920035	4.02E-191
6	Rnf187	0.324830317	2.19E-68
6	Ube2t	0.324406305	5.11E-268
6	Gmps	0.324286334	8.96E-113
6	Tgfa	0.32422729	1.31E-235
6	Smc6	0.324214022	3.20E-69
6	Pls3	0.323772408	5.38E-118
6	Etv4	0.323592303	4.72E-263
6	Fam3c	0.323233387	2.69E-90
7	Ltb	2.3281	5.59E-287
7	Nkg7	2.2311	3.86E-303
7	Ctla2a	2.1826	9.01E-88
7	Ikzf2	1.8822	5.46E-31
7	Cd3g	1.8635	0
7	Gzmb	1.8376	3.41E-201
7	Trbc2	1.8343	0
7	Tnfrsf18	1.8234	0
7	Cd3d	1.7886	0
7	RP23-52N2.1	1.7551	4.09E-39
7	Il2rb	1.7216	0
7	Ptprcap	1.7140	0
7	Tnfrsf9	1.6788	1.49E-69
7	Icos	1.6656	0
7	Ms4a4b	1.6136	3.26E-170
7	Gimap3	1.5916	0
7	Tnfrsf4	1.5665	4.75E-224
7	Trbc1	1.5346	0
7	Vps37b	1.4897	3.26E-40
7	Cd2	1.4746	1.24E-211
7	Hcst	1.4730	7.95E-66
7	Il2ra	1.4613	1.20E-253

7	Gimap1	1.4595	0
7	Il1rl1	1.4375	8.95E-91
7	Ets1	1.4312	1.42E-43
7	Cd69	1.4185	3.45E-97
7	Cd3e	1.3618	3.07E-111
7	Ccnd2	1.3603	7.91E-48
7	Pdcd4	1.3103	1.67E-23
7	Satb1	1.3099	9.59E-146
7	Peli1	1.3044	1.45E-26
7	Tnfaip3	1.2880	1.86E-25
7	Trac	1.2561	9.36E-197
7	Lat	1.2481	0
7	Bcl2	1.2412	3.41E-25
7	Il7r	1.2280	2.52E-70
7	Ablim1	1.2121	4.96E-80
7	Rora	1.1967	1.19E-27
7	Akap13	1.1867	6.61E-22
7	Lck	1.1823	3.98E-223
7	Gata3	1.1752	8.57E-08
7	Skap1	1.1416	2.35E-288
7	Thy1	1.1331	6.81E-50
7	Cd28	1.1292	7.55E-240
7	Mbnl1	1.1266	3.49E-24
7	Stk17b	1.1072	1.76E-29
7	Crem	1.0623	1.22E-09
7	Rac2	1.0622	7.57E-38
7	Ptpn22	1.0480	3.13E-73
7	Fam107b	1.0459	2.27E-17
7	Dennd4a	1.0368	1.99E-13
7	Rgs1	1.0367	5.21E-09
7	Nr3c1	1.0296	0.000974
7	P2ry10	1.0257	1.43E-132
7	Gimap4	1.0246	2.33E-200
7	Ctsw	1.0217	8.14E-214
7	Ramp1	1.0136	3.96E-28
7	Inpp4b	0.9953	4.22E-61
7	Zc3hav1	0.9901	1.12E-09
7	H2-Q7	0.9817	0.00016
7	Il2rg	0.9649	3.08E-20
7	Ppp1r16b	0.9649	4.71E-155
7	Shisa5	0.9620	5.11E-17
7	Selplg	0.9599	5.09E-23
7	Nfkbid	0.9534	0.000147
7	Itgb7	0.9388	8.09E-41
7	D16Ertd472e	0.9376	2.86E-14
7	Tspan13	0.9366	3.05E-10
7	Arhgap45	0.9268	1.97E-09
7	Samsn1	0.9174	0.000371
7	Celf2	0.9168	3.55E-08
7	Ptprc	0.9150	1.90E-23
7	1-Sep	0.9100	2.50E-74
7	Rhoh	0.8998	5.37E-10
7	Pim1	0.8978	3.25E-06
7	Xist	0.8860	1.36E-36
7	Emb	0.8583	2.29E-05
7	Cd52	0.8537	8.46E-28
7	Ifngr1	0.8328	0.015913
7	Vgll4	0.8318	6.17E-07
7	Tmsb10	0.8229	2.02E-50
7	Rapgef6	0.8211	0.039098
7	Clk1	0.7903	0.000733

7	Ptpn18	0.7683	1.57E-13
7	RP23-71J17.1	0.7656	9.13E-12
7	Rps27	0.7400	3.79E-90
7	Nfkbia	0.7294	9.42E-15
7	Dnaja1	0.7238	1.58E-10
7	Fyb	0.7205	0.000444
7	Itgal	0.7139	0.000105
7	H2-K1	0.7130	1.56E-36
7	Cytip	0.6717	0.001398
7	Btg1	0.5970	1.55E-15
7	Pglyrp1	0.5915	2.37E-37
7	Ddx5	0.573493693	4.77E-14
7	Rbm39	0.55783139	2.81E-08
7	H2-D1	0.547354815	2.05E-24
7	Tmsb4x	0.546195396	6.91E-38
7	Rpl17	0.542959652	5.77E-42
7	Rps15a	0.541063985	1.36E-50
7	Rps13	0.527356354	1.44E-47
7	Rpl13a	0.504743169	1.67E-17
7	Rps23	0.499795868	6.90E-52
7	Junb	0.486182125	6.61E-06
7	Rpl23a	0.483461841	3.01E-40
7	Rpl37a	0.481019535	2.85E-58
7	Rps24	0.474821495	1.08E-38
7	Sub1	0.467373784	2.40E-08
7	Rpl21	0.46161267	4.96E-21
7	Rpl34	0.457582322	7.09E-41
7	Rpl27	0.444962493	2.91E-22
7	Furin	0.441385528	0.000473
7	Rpl18a	0.431370068	3.15E-41
7	Sh3bgrl3	0.430438287	3.04E-05
7	Uqcrh	0.429086999	1.25E-11
7	Coro1a	0.428947991	0.011956
7	Rpl18	0.420732261	8.39E-21
7	Rps4x	0.417487727	1.24E-28
7	Rps3	0.416409418	3.85E-27
7	Rps14	0.409845175	1.61E-29
7	Rps7	0.402803438	2.52E-19
7	Rpl12	0.396433026	1.01E-07
7	Rps21	0.396302739	2.99E-21
7	Rps6	0.39603683	2.12E-10
7	Snrpg	0.392386402	4.96E-05
7	Pfdn5	0.385746041	8.40E-07
7	Irf8	0.379526279	0.048381
7	Fau	0.37844694	1.79E-32
7	Rpl36a	0.373462029	1.04E-14
7	Rpl11	0.369829261	7.15E-24
7	Uba52	0.365831645	4.87E-25
7	Rps11	0.364791673	1.15E-21
7	Rpl9	0.364665664	3.34E-20
7	Oaz1	0.349563039	1.38E-14
7	Rps15	0.347076107	2.29E-20
7	Malat1	0.344777054	2.34E-19
7	Rpl4	0.34334344	3.93E-07
7	B2m	0.333273947	1.21E-17
7	Rpl27a	0.33281336	1.96E-16
7	Klf3	0.331330549	0.017959
7	Eif3f	0.327486432	0.014242
7	Rplp2	0.327191862	1.81E-23
8	S100a9	5.3302	1.33E-145
8	S100a8	5.2712	9.20E-138

8	Retnig	4.4005	8.15E-214
8	Wfdc21	3.8465	0
8	G0s2	3.8385	0
8	Stfa3	3.5683	7.84E-284
8	RP24-83D14.4	3.3831	4.83E-262
8	Ifitm1	3.1678	8.39E-149
8	Wfdc17	2.8117	2.30E-100
8	Ifitm6	2.7870	2.48E-67
8	Pglyrp1	2.7792	0
8	Lcn2	2.6192	1.43E-106
8	Prok2	2.6129	0
8	Stfa2	2.5509	0
8	Stfa2l1	2.3239	0
8	Hdc	2.3113	0
8	Csf3r	2.1339	4.09E-228
8	Cxcl2	2.0687	7.38E-49
8	Lrg1	2.0219	3.99E-109
8	Hp	1.9969	2.04E-116
8	Ccl3	1.9961	1.75E-11
8	Clec4e	1.9026	3.94E-97
8	Il1r2	1.8768	3.00E-120
8	Msrb1	1.7780	5.51E-97
8	Mxd1	1.7550	1.71E-85
8	Alox5ap	1.7351	1.03E-72
8	Asprv1	1.7313	1.60E-286
8	Grina	1.7288	9.27E-83
8	Hcar2	1.6603	1.67E-85
8	Acod1	1.6597	2.64E-178
8	Upp1	1.5644	5.53E-52
8	Clec4d	1.5284	4.60E-65
8	Slc16a3	1.5284	4.38E-59
8	Mcemp1	1.5210	2.22E-96
8	Cd300lf	1.5155	8.28E-58
8	Lmnb1	1.4993	1.12E-46
8	Igfbp6	1.4916	1.31E-54
8	Samsn1	1.4693	3.88E-50
8	Il1b	1.4632	1.00E-41
8	S100a11	1.4568	1.29E-76
8	Cxcr2	1.4563	3.16E-271
8	Cebpb	1.3727	1.27E-96
8	RP23-101F14.3	1.3610	4.51E-32
8	Trem1	1.3586	6.88E-35
8	Mmp8	1.3423	1.45E-86
8	Emilin2	1.3174	4.29E-49
8	Slfn4	1.3087	5.87E-87
8	Fbxl5	1.3048	6.55E-21
8	Fpr2	1.3046	7.14E-102
8	Mirt2	1.2969	7.74E-220
8	Sell	1.2816	9.58E-126
8	Pnrc1	1.2760	5.23E-27
8	Cd14	1.2724	7.98E-57
8	Stx11	1.2622	4.19E-66
8	Plaur	1.2613	6.62E-28
8	Il1f9	1.2432	4.64E-246
8	Hilpda	1.2288	3.99E-10
8	Sorl1	1.2211	1.09E-33
8	Tarm1	1.2164	2.17E-38
8	Fth1.1	1.2019	4.89E-70
8	Cd24a	1.2014	1.28E-34
8	Chil1	1.1911	0
8	Litaf	1.1905	3.13E-49

8	Ly6g	1.1762	4.30E-287
8	Map1lc3b	1.1711	2.74E-36
8	Slc2a3	1.1570	7.21E-85
8	Rac2	1.1558	4.00E-25
8	Pim1	1.1234	8.49E-23
8	Steap4	1.1229	8.55E-61
8	Ifitm2	1.1159	1.39E-60
8	Pfkfb3	1.1102	1.65E-13
8	RP23-214L4.1	1.1082	4.49E-07
8	Cd52	1.0998	1.44E-48
8	Cd33	1.0864	1.03E-10
8	Prr13	1.0795	5.26E-14
8	Gmfg	1.0636	2.90E-24
8	Dedd2	1.0613	2.00E-11
8	Lilr4b	1.0561	9.35E-26
8	Hist2h2aa2	1.0518	6.39E-11
8	Ypel3	1.0396	1.73E-11
8	Mcl1	1.0186	2.26E-44
8	Zyx	1.0145	1.33E-06
8	Ets2	1.0114	5.57E-12
8	Selplg	1.0058	5.50E-14
8	Arg2	1.0015	3.04E-17
8	Mmp9	0.9969	3.63E-28
8	Lcp1	0.9907	4.37E-37
8	Ppp1r3b	0.9893	5.34E-24
8	Prdx5	0.9666	2.46E-52
8	Hcst	0.9643	6.28E-22
8	Id1	0.9538	2.01E-15
8	Tgoln1	0.9433	0.019209
8	Rab20	0.9345	1.72E-06
8	Btg2	0.9211	0.028442
8	Ptprc	0.8821	1.07E-15
8	Tpd52	0.8818	1.39E-13
8	Ostf1	0.8783	1.36E-24
8	Vasp	0.8617	0.002271
8	Snap23	0.8601	0.001013
8	Ier3	0.8582	1.08E-14
8	RP24-530N5.3	0.8486	8.99E-08
8	Tspan13	0.8268	1.34E-06
8	Ccr1	0.8258	2.68E-06
8	Samd9l	0.8254	1.43E-07
8	Pygl	0.8128	5.75E-08
8	Tspo	0.8091	3.82E-24
8	Tyrobp	0.8059	1.22E-52
8	RP23-411N10.2	0.7960	6.44E-06
8	Csrnp1	0.7940	0.044035
8	Gpsm3	0.7850	8.15E-05
8	Lsp1	0.7839	0.001629
8	C5ar1	0.7800	4.26E-06
8	Ptafr	0.7717	0.004519
8	Gpcpd1	0.7626	1.98E-08
8	Srgn	0.7615	2.73E-50
8	Cdkn1a	0.7594	3.32E-06
8	Cd44	0.7577	6.06E-12
8	Adipor1	0.7556	1.39E-06
8	Coro1a	0.7549	1.05E-19
8	Clec7a	0.7528	0.031259
8	Mrpl33	0.7423	5.80E-13
8	Gadd45a	0.7357	0.011385
8	Ltb4r1	0.7259	5.32E-06
8	Plek	0.7203	0.00027

8	Fxyd5	0.7072	6.09E-19
8	Sat1	0.6982	2.18E-22
8	Picalm	0.6909	4.28E-12
8	Cd53	0.6860	6.24E-21
8	H3f3a	0.6855	1.93E-41
8	Cd9	0.6767	1.25E-17
8	Slpi	0.6721	5.04E-12
8	Dusp1	0.6694	1.67E-05
8	Cox17	0.6668	3.59E-09
8	Btg1	0.6643	7.50E-15
8	Malat1	0.6453	8.85E-45
8	Lst1	0.6292	4.71E-06
8	Gabarap	0.6251	4.98E-29
8	Adam8	0.6119	0.0025
8	Rsrp1	0.6113	0.000274
8	Fcer1g	0.6086	2.91E-26
8	S100a6	0.5969	9.40E-19
8	Spi1	0.5958	0.010725
8	Ncf4	0.5894	0.000475
8	Selk	0.567442076	0.003351
8	Eif1	0.556940163	7.91E-35
8	Hmgb2	0.541064594	0.000148
8	Sdcbp	0.512664146	2.31E-08
8	Actg1	0.503020451	2.79E-29
8	Ddx5	0.501895624	3.43E-12
8	Atp6v1g1	0.497173993	3.38E-07
8	Ftl1	0.482283157	2.96E-18
8	Pfn1	0.412853487	3.89E-19
8	Junb	0.406444878	0.003165
8	Arpc3	0.402635914	5.69E-05
8	Sh3bgrl3	0.392556716	0.019053
8	Txn1	0.379342086	2.75E-05
8	Fcgr3	0.36724405	0.013786
8	Aldoa	0.335722788	0.034801
9	Ighm	3.7590	1.05E-21
9	Ccl17	2.3532	2.27E-101
9	Cst3	2.0878	7.03E-14
9	H2-Eb1	1.5664	9.29E-20
9	H2-Aa	1.5147	2.98E-23
9	Mzb1	1.4231	4.15E-165
9	Cd74	1.4227	5.00E-33
9	H2-Ab1	1.3713	6.15E-17
9	Cytip	1.3172	1.55E-59
9	Rnase6	1.3131	9.68E-111
9	Lsp1	1.2984	1.60E-34
9	H2-Ea-ps	1.2961	1.30E-34
9	Iglc3	1.2297	1.24E-243
9	Tmem123	1.1734	2.02E-05
9	Tbc1d4	1.1684	7.22E-12
9	Irf8	1.1576	0.000326
9	Syngr2	1.1460	7.92E-23
9	Tspan13	1.1189	6.01E-47
9	Dnajc7	1.0768	2.17E-06
9	H2-DMb2	1.0571	1.82E-19
9	Napsa	1.0266	6.06E-47
9	Plac8	1.0149	1.44E-09
9	Pkib	1.0131	1.07E-12
9	Bcl2a1d	0.9745	1.14E-05
9	Selplg	0.9622	8.27E-25
9	Cd200	0.9549	1.49E-44
9	Ramp3	0.9462	5.69E-53

9	Rabgap1l	0.9279	9.45E-20
9	Ccnd2	0.9242	4.44E-17
9	Crip1	0.9213	4.74E-09
9	Smc6	0.8978	1.13E-06
9	Bcl11a	0.8975	5.62E-26
9	RP23-69P8.2	0.8750	7.82E-07
9	Plbd1	0.8656	5.70E-06
9	Rel	0.8469	1.49E-10
9	St8sia4	0.8412	9.23E-16
9	Stk17b	0.8145	2.60E-07
9	Calm1	0.7964	2.68E-07
9	Ckb	0.7694	2.75E-07
9	Gnb4	0.7634	0.000107
9	Cd37	0.7630	3.06E-08
9	H2-DMb1	0.7616	0.000104
9	Cbfa2t3	0.7553	3.28E-109
9	Rogdi	0.7484	2.36E-17
9	Abhd17b	0.7403	0.007616
9	Tmsb4x	0.7301	7.73E-08
9	Tnip3	0.7224	4.78E-12
9	Myo1g	0.7163	2.19E-21
9	Ptprcap	0.7075	3.28E-48
9	Batf3	0.7021	8.92E-25
9	Traf1	0.6957	0.001065
9	Sub1	0.6936	3.15E-19
9	Flt3	0.6780	4.27E-290
9	Etv3	0.6623	2.43E-08
9	P2ry10	0.6623	1.03E-89
9	Mef2c	0.6619	2.36E-06
9	Itga4	0.6480	6.58E-17
9	Mbnl1	0.6438	1.66E-08
9	Itgb7	0.6300	1.27E-42
9	Psmb8	0.6280	6.52E-08
9	Map4k1	0.6113	3.90E-33
9	Arhgap30	0.6092	7.26E-07
9	Malt1	0.6072	8.84E-06
9	Wdfy4	0.6067	3.43E-19
9	Arpc5l	0.6062	0.000126
9	Crem	0.57699566	1.66E-05
9	Tbc1d8	0.575313199	2.49E-29
9	Coro1a	0.565839811	2.37E-07
9	Dock10	0.557279055	1.49E-07
9	Cd48	0.553283129	5.54E-08
9	Psme1	0.544492897	4.30E-05
9	Bloc1s2	0.544194584	7.79E-07
9	Nfkb1	0.529133082	0.00751
9	Xist	0.525330373	1.42E-10
9	Ptprc	0.506918906	3.35E-05
9	Sec11c	0.503445053	0.02796
9	Pmaip1	0.502366357	2.88E-11
9	Gpr171	0.496621985	5.71E-05
9	Rps27	0.493264383	1.51E-14
9	Lactb	0.49280188	2.09E-08
9	Rac2	0.485655302	0.02529
9	Syk	0.482222603	0.028585
9	Ppm1m	0.481811889	0.00033
9	Dennd4a	0.471802816	0.005725
9	Cd47	0.468071908	0.001405
9	Fyn	0.461047027	0.025283
9	Pim1	0.434343586	0.003726
9	Pkp3	0.434169485	4.46E-06

9	Rftn1	0.432576523	0.014403
9	Irf5	0.426577567	4.28E-05
9	Cd24a	0.413053914	0.017064
9	Arhgap45	0.404042269	0.002502
9	Ndufa6	0.402875612	0.000217
9	Malat1	0.386255038	8.50E-08
9	Rps19	0.371667857	0.01507
9	Rps4x	0.356976068	1.42E-07
9	Snx20	0.356799389	0.019232
9	Ankrd44	0.355522223	0.000117
9	Rpl17	0.354173689	1.80E-09
9	Rpl4	0.350224874	1.95E-06
9	Rps11	0.346596094	1.11E-11
9	Eif3f	0.343589672	0.004626
9	Rps7	0.323683605	0.032901
10	Igfbp3	3.1096	3.36E-98
10	Ctla2a	3.0588	1.72E-30
10	Igfbp7	3.0383	9.48E-20
10	Col4a1	3.0001	1.51E-35
10	Fabp4	2.8249	4.35E-77
10	Gpihbp1	2.5731	0
10	Col4a2	2.5656	1.25E-53
10	Cdh5	2.4824	0
10	Cav1	2.4269	4.01E-86
10	Rbp1	2.3260	9.22E-23
10	Egfl7	2.2268	9.17E-100
10	Aqp1	2.2142	2.10E-49
10	Plvap	2.1428	6.54E-228
10	Gng11	2.1231	3.69E-21
10	Esam	2.0276	0
10	Mcam	2.0057	5.22E-138
10	Mest	1.9906	1.84E-96
10	Pecam1	1.9791	8.99E-36
10	Prss23	1.9005	2.24E-33
10	Crip2	1.8892	2.60E-56
10	Cldn5	1.8867	0
10	Ramp2	1.8460	2.17E-149
10	Sparcl1	1.8069	2.69E-41
10	Ecscr	1.7898	6.48E-72
10	Sparc	1.7767	4.06E-13
10	Flt1	1.7672	5.56E-44
10	Hspg2	1.7654	1.42E-26
10	Ppic	1.7480	4.51E-38
10	Fkbp1a	1.6959	2.39E-14
10	Pdlim1	1.6646	9.93E-47
10	Adgrf5	1.6625	3.29E-249
10	Trp53i11	1.6522	2.83E-82
10	Cd93	1.6402	6.84E-29
10	Emcn	1.6104	0
10	AC164564.3	1.5830	1.34E-14
10	Vwa1	1.5782	2.74E-189
10	Eng	1.5718	1.28E-30
10	Cd34	1.5634	2.78E-63
10	Apln	1.5214	1.50E-23
10	Ptprb	1.4816	0
10	Tcf4	1.4633	6.26E-11
10	Col18a1	1.4339	1.22E-24
10	Csrp1	1.4315	2.61E-19
10	Adgrl4	1.4295	0
10	Kdr	1.4142	1.16E-216
10	Adamts9	1.3868	1.03E-105

10	Myct1	1.3646	0
10	Tspan6	1.3425	5.16E-64
10	Ets1	1.3127	8.53E-19
10	Slc9a3r2	1.2955	2.20E-44
10	Epas1	1.2942	1.56E-33
10	Prkcdbp	1.2900	4.21E-14
10	S1pr1	1.2780	6.93E-88
10	Icam2	1.2776	1.26E-148
10	Cxx1a	1.2662	1.32E-41
10	Aplnr	1.2610	0
10	Plxnd1	1.2529	1.01E-30
10	Podxl	1.2390	1.17E-62
10	Ptrf	1.2170	5.79E-19
10	Tm4sf1	1.2161	3.01E-12
10	Rhoc	1.2110	4.12E-10
10	Nid1	1.2098	3.71E-37
10	Vamp5	1.2003	9.51E-65
10	Lama4	1.2000	7.34E-43
10	Cdh13	1.1932	0
10	Apbb2	1.1792	2.38E-21
10	Sema7a	1.1536	1.80E-41
10	Smad1	1.1531	4.93E-16
10	Tmem88	1.1497	1.87E-181
10	Pkig	1.1345	9.81E-15
10	Tmem252	1.1338	0
10	Prnp	1.1311	2.79E-14
10	Tpm4	1.1292	1.97E-10
10	Ece1	1.1212	2.65E-12
10	N4bp3	1.1206	3.56E-37
10	Sox18	1.1204	0
10	Adamts4	1.1178	7.66E-44
10	Serpinh1	1.1135	2.40E-12
10	Cd200	1.1074	1.22E-55
10	AC092855.1	1.1072	7.97E-21
10	Col15a1	1.1017	9.83E-53
10	Vim	1.1007	1.28E-07
10	Mast4	1.0924	5.93E-17
10	Plpp1	1.0883	1.82E-43
10	Actn4	1.0784	9.04E-10
10	Itga6	1.0739	4.84E-20
10	Prex2	1.0689	0
10	Lxn	1.0638	7.04E-12
10	Myo10	1.0624	1.74E-35
10	Cav2	1.0615	6.32E-53
10	Id1	1.0520	8.96E-23
10	Tspan7	1.0412	0
10	Ehd4	1.0372	1.64E-10
10	Nck1	1.0364	7.31E-15
10	Gbp4	1.0343	2.45E-14
10	Lamc1	1.0341	1.96E-17
10	Inhbb	1.0315	1.61E-54
10	Mmrn2	1.0285	0
10	Fam101b	1.0282	3.59E-30
10	Fermt2	1.0263	1.84E-18
10	Fam43a	1.0247	1.68E-68
10	Tmsb10	1.0128	2.31E-05
10	Cald1	1.0071	1.47E-09
10	Cnn3	1.0020	8.98E-11
10	Cd81	1.0010	1.70E-06
10	Fam167b	0.9982	3.95E-70
10	Gimap4	0.9958	4.40E-117

10	Fli1	0.9932	1.23E-19
10	Cyyr1	0.9918	0
10	Arhgap29	0.9911	6.80E-31
10	RP23-101F14.3	0.9848	4.90E-15
10	Gimap1	0.9844	8.62E-116
10	Rhoj	0.9789	3.58E-23
10	Sptbn1	0.9754	1.10E-08
10	Hes1	0.9729	9.25E-10
10	4-Sep	0.9714	5.57E-168
10	Iigp1	0.9632	1.13E-07
10	Oaz2	0.9631	7.43E-06
10	Heg1	0.9556	2.06E-27
10	Adamts1	0.9545	9.96E-16
10	Calm1	0.9493	4.40E-08
10	Grrp1	0.9460	0
10	Esm1	0.9460	1.03E-210
10	Fscn1	0.9423	3.12E-12
10	Rgcc	0.9419	1.86E-07
10	Abhd17a	0.9372	9.73E-16
10	Marcksl1	0.9351	6.26E-09
10	Ptp4a3	0.9311	1.01E-09
10	Tns1	0.9309	5.24E-30
10	Rapgef5	0.9292	4.28E-84
10	Lgals9	0.9268	1.16E-10
10	Meox2	0.9241	3.77E-211
10	Cpe	0.9176	2.89E-05
10	Itgb1	0.9140	5.49E-08
10	Ccdc85b	0.9130	1.63E-11
10	Insr	0.9114	2.10E-09
10	Trim37	0.8895	1.58E-30
10	Tie1	0.8878	7.52E-225
10	Sdpr	0.8825	7.35E-24
10	Rbpms	0.8821	1.10E-27
10	Nid2	0.8746	1.44E-99
10	Palmd	0.8671	0
10	Cdipt	0.8584	3.09E-07
10	Robo4	0.8582	0
10	Efna1	0.8569	2.19E-15
10	Tjp1	0.8564	6.42E-16
10	RP23-349I9.6	0.8538	1.21E-15
10	Gimap5	0.8530	2.05E-166
10	Slfn5	0.8518	2.03E-07
10	Ehd2	0.8508	7.87E-18
10	Lims1	0.8499	1.81E-09
10	Pxdn	0.8403	1.97E-27
10	Pcdh1	0.8364	1.38E-110
10	Kcne3	0.8356	1.81E-160
10	Jam2	0.8314	8.73E-168
10	Plekhg1	0.8277	1.11E-234
10	Mecom	0.8272	1.64E-118
10	Eogt	0.8234	2.36E-16
10	Kank3	0.8144	4.38E-10
10	Tax1bp3	0.8125	5.74E-08
10	Car4	0.8097	9.13E-21
10	Ablim1	0.8047	1.84E-21
10	Lamb1	0.8017	8.38E-08
10	Nckap1	0.7997	2.29E-11
10	Nes	0.7965	4.39E-23
10	Pdlim5	0.7955	3.00E-09
10	Hspa12b	0.7920	1.26E-210
10	Crim1	0.7918	2.07E-16

10	RP23-381H23.2	0.7914	0
10	Dgkh	0.7906	4.64E-28
10	Cxx1b	0.7862	9.00E-38
10	Sema6d	0.7846	1.50E-32
10	Tspan9	0.7789	6.15E-42
10	Pdgfb	0.7764	1.53E-20
10	Mfng	0.7752	1.89E-30
10	Dll4	0.7748	0
10	Akap12	0.7738	2.74E-35
10	Afap1l1	0.7704	3.56E-270
10	Ctnnb1	0.7702	0.004243
10	Tuba1a	0.7699	0.001409
10	Id3	0.7685	0.011613
10	Ldb2	0.7679	0
10	Dysf	0.7679	0
10	Macf1	0.7671	2.72E-07
10	Tnfaip1	0.7669	3.24E-11
10	Hmgn5	0.7657	3.28E-20
10	Endod1	0.7633	8.84E-16
10	Hyal2	0.7591	1.92E-19
10	Nus1	0.7571	4.10E-07
10	Slc38a2	0.7566	9.90E-05
10	Tspan8	0.7506	6.29E-206
10	Kif5b	0.7486	0.000359
10	Rasip1	0.7486	0
10	Kitl	0.7480	1.72E-18
10	Stc1	0.7467	1.74E-27
10	Upp1	0.7455	2.64E-13
10	Calu	0.7424	6.67E-08
10	Sema3f	0.7410	3.98E-16
10	Ifi203-ps	0.7409	6.03E-133
10	Rab11a	0.7404	0.000169
10	Yes1	0.7385	2.66E-13
10	Xist	0.7381	0.003822
10	Ppp1r11	0.7371	0.000149
10	Zbtb20	0.7363	2.08E-08
10	Apold1	0.7350	4.95E-136
10	Edn1	0.7323	1.18E-30
10	Plk2	0.7318	2.56E-05
10	Sptan1	0.7313	1.56E-07
10	Notch4	0.7274	9.07E-203
10	Cd36	0.7270	4.24E-60
10	Procr	0.7236	6.40E-19
10	Ptprm	0.7166	6.26E-143
10	Tinagl1	0.7161	5.89E-05
10	Clic4	0.7157	0.00121
10	Efnb2	0.7154	4.74E-19
10	Nrp1	0.7147	8.14E-10
10	Clca2	0.7134	0
10	Tnfrsf9	0.7096	3.88E-23
10	Abcg2	0.7096	1.04E-13
10	Fbln5	0.7091	1.42E-45
10	Bvht	0.7090	4.80E-34
10	Snrk	0.7080	1.93E-25
10	Itm2c	0.7072	0.012224
10	Peg3	0.7062	1.25E-75
10	Sox4	0.7056	0.002394
10	Dlc1	0.7048	5.86E-32
10	Rasgrp3	0.7043	0
10	Synpo	0.7032	1.73E-20
10	Npdc1	0.7030	2.74E-10

10	Mpzl1	0.7025	4.45E-15
10	F11r	0.6997	5.08E-27
10	Myh9	0.6986	3.24E-05
10	Mylk	0.6975	8.20E-34
10	Calcrl	0.6947	8.86E-42
10	Ctnnbip1	0.6939	2.72E-05
10	Gnai2	0.6923	1.86E-05
10	Elk3	0.6922	7.39E-06
10	Adam10	0.6919	1.57E-06
10	Nrarp	0.6894	8.04E-29
10	Ly6a	0.6893	1.03E-07
10	Ralb	0.6888	0.000269
10	Arhgap31	0.6876	3.70E-10
10	Ppp1r2	0.6859	0.001061
10	Actn1	0.6853	8.99E-05
10	Adgrg1	0.6823	5.79E-130
10	Ephb4	0.6820	8.73E-15
10	Ckap4	0.6770	5.49E-06
10	Dpysl3	0.6762	1.90E-30
10	Lrg1	0.6751	3.54E-07
10	Gimap6	0.6734	1.57E-101
10	Rell1	0.6732	2.42E-09
10	Thsd1	0.6704	1.37E-169
10	Ctnnd1	0.6693	4.08E-05
10	Chst1	0.6690	1.50E-38
10	Fkbp10	0.6677	1.49E-15
10	Alad	0.6675	2.06E-06
10	Pvrl2	0.6663	2.69E-14
10	Wwtr1	0.6647	2.11E-08
10	Eva1b	0.6644	8.15E-06
10	Itga1	0.6641	8.60E-12
10	Luzp1	0.6626	0.011098
10	Angpt2	0.6541	1.31E-14
10	Msn	0.6530	0.000326
10	Rab2a	0.6520	0.006418
10	RP23-211B19.2	0.6515	8.79E-06
10	Myadm	0.6514	2.56E-08
10	Exoc3l4	0.6513	8.39E-11
10	Scarb1	0.6512	9.95E-11
10	Lrrc8a	0.6507	2.26E-06
10	Ctnna1	0.6500	0.003792
10	Dock4	0.6460	0.009905
10	Adam15	0.6459	4.46E-06
10	Dynlt3	0.6416	0.000187
10	Sox17	0.6398	0
10	Nedd4	0.6393	1.36E-06
10	Hoxd8	0.6336	1.83E-63
10	Uaca	0.6332	3.88E-30
10	Gnas	0.6330	0.035134
10	Pomp	0.6307	0.007097
10	Eml1	0.6298	1.23E-39
10	Nfib	0.6281	1.53E-06
10	Npr1	0.6244	1.27E-48
10	Jup	0.6240	0.001077
10	Myo1b	0.6235	7.20E-15
10	Parva	0.6229	3.14E-07
10	Ywhah	0.6227	0.00784
10	Ppfibp1	0.6193	6.57E-07
10	Bmp2	0.6180	1.55E-44
10	Fgd5	0.6175	8.35E-183
10	Ppp1r14a	0.6173	2.83E-94

10	Tmem47	0.6145	1.07E-95
10	Ccser2	0.6138	2.02E-12
10	Fam126a	0.6133	8.49E-10
10	Entpd1	0.6125	8.02E-11
10	Akr1b8	0.6114	4.73E-05
10	Ly6c1	0.6112	3.50E-07
10	Plcb1	0.6070	1.29E-35
10	Rgs3	0.6036	1.21E-13
10	Kctd17	0.6012	4.90E-22
10	Triobp	0.5990	2.04E-07
10	Zeb1	0.5975	8.74E-12
10	Mgll	0.5904	5.34E-76
10	Clec14a	0.5902	6.46E-202
10	Swap70	0.5868	2.74E-05
10	Ushbp1	0.5866	0
10	Bcr	0.5863	2.67E-06
10	Morf4l1	0.583044354	0.000224
10	Itgb3	0.582528613	7.98E-19
10	Prkar1a	0.582026703	3.61E-06
10	F2r	0.580779058	1.55E-07
10	Ypel2	0.580361122	4.83E-48
10	Dynll1	0.580220705	0.000311
10	Cmtm8	0.577497912	1.53E-43
10	Lpp	0.575439134	0.011866
10	Bmpr2	0.575421042	0.000295
10	Tek	0.574892311	8.51E-138
10	Plpp3	0.5725741	2.16E-18
10	Gnb4	0.57245165	5.41E-10
10	Bnip2	0.571775991	4.49E-06
10	11-Sep	0.569822691	8.16E-05
10	Flnb	0.569130029	1.87E-11
10	Myl6	0.564838536	0.000109
10	Ppm1f	0.564835767	2.02E-29
10	Mob4	0.564820265	0.041826
10	AC131992.1	0.563408838	0
10	RP24-171J16.5	0.563197186	4.87E-101
10	Cenpt	0.561206732	3.81E-10
10	She	0.560793071	0
10	Sox7	0.560487911	0
10	Klk8	0.558839979	5.99E-12
10	Grap	0.557851615	1.10E-18
10	Ddah2	0.555754896	0.017675
10	Stx6	0.55554999	2.83E-09
10	Spata13	0.553959448	4.69E-16
10	Unc45b	0.551749562	9.84E-120
10	Lmcd1	0.550919958	6.07E-57
10	Cyb5a	0.549512723	0.000167
10	Cd151	0.549252985	8.78E-05
10	Map4k3	0.54717723	0.00015
10	Pde4b	0.546508568	3.25E-05
10	7-Sep	0.546130799	0.004765
10	Cnih1	0.545852174	0.027611
10	Nudt4	0.543922367	0.001707
10	Arhgef12	0.543481541	8.62E-10
10	Scn1b	0.543109541	7.73E-18
10	Bcl6b	0.543083782	7.19E-291
10	Nos3	0.542562625	9.65E-188
10	Hdac7	0.541455296	0.001517
10	Ebf1	0.540213114	5.36E-11
10	Gchfr	0.539475585	2.41E-11
10	Myl12a	0.539049049	0.011693

10	RP23-21D6.1	0.537973094	7.99E-07
10	Tmem204	0.537800903	4.19E-254
10	Usp14	0.531840471	3.23E-06
10	Selm	0.529139635	2.04E-05
10	Tshz2	0.528451648	3.67E-15
10	Angptl4	0.527904747	5.50E-07
10	C1qtnf6	0.52706479	8.68E-12
10	Dbn1	0.527021325	7.46E-05
10	Kit	0.526153913	9.16E-175
10	RP23-10P23.3	0.525380896	0
10	Mgat4b	0.524959101	0.001877
10	Slc12a2	0.523455196	9.35E-11
10	Clec1a	0.520889871	3.97E-239
10	Lrat	0.518957973	2.10E-115
10	S100a16	0.517656185	1.77E-08
10	Pls3	0.513940444	3.82E-08
10	Fam198b	0.512391026	2.16E-25
10	Grb10	0.505448802	5.60E-19
10	RP23-375N21.2	0.50533711	6.35E-45
10	Arhgef15	0.505176198	1.44E-82
10	10-Sep	0.504958795	0.020099
10	Pde8a	0.504140868	4.68E-15
10	RP24-360B3.2	0.504039156	1.14E-22
10	Adcy4	0.50269117	2.90E-167
10	Prkch	0.500371833	2.38E-17
10	Casp8	0.500327659	0.000656
10	Jag1	0.497867286	0.008492
10	Meox1	0.493139476	1.25E-183
10	Klc1	0.492310916	0.022809
10	Bcam	0.490224387	1.70E-35
10	Adgrl2	0.489800258	0.009182
10	Bace2	0.488940573	2.48E-117
10	Gdpd5	0.488392898	2.89E-05
10	Mmp15	0.488089134	9.67E-216
10	Ppp1r16b	0.487620783	5.44E-59
10	Rps6ka3	0.486660293	1.89E-05
10	RP23-106P7.2	0.486627129	0.000215
10	RP24-247A21.1	0.486064174	0
10	Pglyrp1	0.485382369	5.28E-24
10	Ywhab	0.484692103	0.02423
10	Itga5	0.484624576	9.54E-09
10	Rapgef3	0.484188946	6.81E-11
10	Gprc5b	0.483752226	1.32E-28
10	Cracr2b	0.483545431	5.26E-18
10	Parvb	0.483338166	3.29E-10
10	Snn	0.483069447	1.58E-15
10	Plat	0.482840065	0.045934
10	Hoxd9	0.481726698	8.78E-170
10	Mprip	0.477546282	0.005488
10	Fkbp9	0.477174323	1.57E-08
10	Dst	0.474023063	1.50E-05
10	Stim2	0.472471987	7.57E-07
10	Ppp3ca	0.471543661	0.010649
10	Sorbs2	0.471150124	3.79E-34
10	Dock9	0.470993367	2.34E-09
10	Tfpi	0.468792698	7.24E-21
10	Ipo11	0.465363093	3.36E-21
10	AC163329.3	0.465218319	8.85E-10
10	Lrrc32	0.464056675	1.07E-06
10	Itpr1	0.461483318	9.54E-05
10	Ripk3	0.461454208	0.015898

10	Utrn	0.460332658	1.06E-05
10	Plec	0.460070061	0.003815
10	Ndufaf4	0.459843918	0.010115
10	Dok4	0.459022485	1.07E-09
10	Amotl1	0.457023988	0.000802
10	Scarf1	0.455364848	8.86E-123
10	Plxna2	0.455164288	1.97E-174
10	Pald1	0.453487748	5.38E-08
10	Hoxb7	0.452705654	2.29E-179
10	C1qtnf9	0.452382016	0
10	Ift122	0.45186452	1.31E-26
10	Ptpn12	0.449537789	0.005152
10	Esyt1	0.449509925	0.002206
10	Mkl2	0.448934915	0.00043
10	Ktn1	0.448470192	0.034105
10	Sh2d3c	0.447900373	1.80E-59
10	Bok	0.447792339	1.27E-08
10	Tceal8	0.447099253	7.83E-05
10	Foxp1	0.443195174	0.001676
10	Map4k5	0.442914899	0.003705
10	Fyn	0.442297732	3.65E-06
10	Rasal2	0.441391671	2.44E-06
10	Piezo1	0.440885353	0.006884
10	Cdc42bpa	0.439649286	8.68E-05
10	Armcx2	0.438395249	1.36E-14
10	Tfpi2	0.437922317	2.08E-13
10	Abcc4	0.437162651	6.80E-30
10	Prcp	0.43715409	4.08E-06
10	Rras	0.436836714	0.010916
10	Sacs	0.433782713	4.83E-13
10	Ilf2	0.432452272	0.027073
10	Timp3	0.431869482	8.97E-18
10	P3h2	0.430375784	8.57E-138
10	Btbd3	0.426387579	1.03E-05
10	Gimap9	0.426371406	1.70E-18
10	Lrrc8c	0.426029376	3.47E-06
10	Rnf125	0.425246222	6.48E-68
10	Mxra7	0.424867369	4.61E-29
10	Notch1	0.423675303	0.000167
10	Cd276	0.423366713	0.009302
10	Stard4	0.422008076	6.44E-05
10	Mllt4	0.420564126	2.71E-06
10	Mesdc1	0.419588427	1.27E-06
10	Cd109	0.418389107	6.49E-40
10	Slc30a4	0.417101863	1.34E-05
10	Tspan12	0.413021764	1.55E-53
10	Dusp2	0.412564313	0.000571
10	Csrp2	0.409359757	0.001224
10	Acvrl1	0.409239478	0.000318
10	Ehd3	0.407575015	1.34E-132
10	Tspan13	0.406522713	0.006653
10	Znrf1	0.405040218	0.000104
10	RP23-451C8.2	0.404850477	0
10	Lrrk2	0.403966293	2.92E-10
10	Tmem41a	0.402202355	3.70E-06
10	Tram2	0.401733195	2.46E-07
10	Acer2	0.399584713	1.28E-122
10	Mcf2l	0.397220579	3.56E-39
10	Fmnl3	0.396397376	0.004152
10	Rgs16	0.396139561	2.48E-17
10	Tnfaip8l1	0.395649599	1.60E-23

10	Caskin2	0.395028535	8.77E-11
10	Ubxn7	0.394471204	0.038237
10	Sh3bp5	0.394445434	1.80E-10
10	Smtn	0.394173265	0.000898
10	Nckap5l	0.393560334	5.25E-11
10	Arl2bp	0.393178742	0.005944
10	Amotl2	0.392567293	0.031178
10	Armcx4	0.388545703	1.67E-23
10	Jcad	0.388255312	7.57E-17
10	Kctd10	0.38812867	0.006786
10	Tspan18	0.387574125	1.44E-14
10	Lypla1	0.386992073	0.006207
10	Magi1	0.386465857	1.15E-09
10	Gimap8	0.38617139	3.43E-56
10	Fam57a	0.386113835	1.61E-14
10	Hmg20b	0.385526487	0.00393
10	Cdc42ep2	0.384983536	4.58E-08
10	Fzd6	0.382537735	0.004497
10	Nrep	0.381907089	0.006038
10	Cdc42ep3	0.381634756	2.82E-07
10	Plscr2	0.377764446	2.95E-11
10	Nfic	0.377503822	0.005524
10	Sipa1	0.376972227	0.009773
10	Hras	0.376884407	0.035325
10	Mfap3	0.376871267	5.38E-05
10	Zfp664	0.375006509	0.041452
10	Fam111a	0.374936428	0.000258
10	Arhgap23	0.374606921	4.22E-06
10	Cbfa2t3	0.374508793	1.44E-18
10	Myzap	0.372166392	1.57E-176
10	Cds2	0.366465745	0.008363
10	RP23-162H6.1	0.366443535	0
10	Plod1	0.366123065	0.036833
10	Vamp3	0.366122902	0.017099
10	Chst7	0.365616616	1.33E-191
10	Ppp1r13b	0.364217646	1.99E-16
10	Ddah1	0.364140156	5.37E-09
10	Arhgap18	0.363087567	4.23E-06
10	Smagp	0.361754728	2.73E-12
10	Fzd4	0.359960206	5.64E-61
10	RP23-282C23.1	0.359720248	3.03E-30
10	Ttc9	0.358466706	1.28E-15
10	Mtus1	0.357372339	5.28E-05
10	Lpar6	0.356932346	1.80E-08
10	Sorbs3	0.354184408	5.49E-36
10	RP23-310J6.1	0.353753464	0.027401
10	Slc43a3	0.353272078	1.76E-05
10	Pdia5	0.350738602	1.42E-07
10	Orai1	0.348207046	0.024577
10	Yaf2	0.345324133	0.039381
10	Mall	0.344047341	5.29E-33
10	Kdelr3	0.343810355	0.001453
10	Prkd2	0.342400833	6.08E-06
10	Il6st	0.342106161	0.013804
10	Erc1	0.341625344	2.40E-06
10	Myl4	0.341110442	1.12E-16
10	Bambi	0.340991634	0.000164
10	Msrb3	0.340349206	1.57E-09
10	Arhgef28	0.339825247	1.87E-15
10	Smim10l1	0.33962034	0.044724
10	Myo18a	0.339468226	0.011819

10	Smco4	0.337279044	2.36E-05
10	Gpr4	0.336947699	1.81E-263
10	Rapgef1	0.335866632	0.000148
10	Rapgef4	0.335655219	1.46E-202
10	Nudt14	0.333861804	0.022271
10	Ccny	0.330971731	0.005194
10	Arsa	0.330877687	0.005129
10	Mtmr11	0.330868448	1.57E-05
10	Adarb1	0.329290027	7.24E-05
10	Pdgfd	0.328375043	3.04E-28
10	Rbfox2	0.327822048	0.037626
10	Mycbp	0.327085386	0.036927
10	Ggta1	0.326917313	0.000104
10	Olfml2a	0.326401356	7.94E-278
10	Wsb2	0.325722388	0.034626
10	Tspan15	0.325597485	5.42E-11
10	Sema6b	0.324527697	3.88E-23
10	Jag2	0.324265758	2.80E-15
10	Dennd5b	0.324028848	9.19E-13
10	Cog8	0.324016056	0.004968
10	Fez2	0.323965419	0.012703
10	Map3k20	0.323030032	0.000128
11	Rgs5	3.8505	2.07E-300
11	Acta2	3.3561	6.08E-38
11	Tagln	2.6525	3.43E-57
11	Igfbp7	2.6521	6.69E-17
11	Col4a1	2.6046	2.21E-27
11	Fabp4	2.5331	1.83E-78
11	Sparcl1	2.5150	1.66E-44
11	Col4a2	2.4752	7.08E-47
11	Mgp	2.3211	0.000186
11	Tpm1	2.2994	2.31E-16
11	Tpm2	2.2305	4.45E-45
11	Myl9	2.1690	5.06E-91
11	Ndufa4l2	2.1446	7.89E-88
11	Higd1b	2.1439	0
11	Ctgf	2.1359	0.019142
11	Meg3	2.1197	1.10E-33
11	Col6a3	2.0748	1.62E-25
11	Mylk	2.0716	2.39E-175
11	Ppp1r14a	2.0286	3.00E-207
11	Thy1	1.9700	2.55E-19
11	Gng11	1.9604	1.31E-13
11	RP23-375N21.2	1.8399	2.87E-290
11	Ebf1	1.8372	6.01E-43
11	Timp3	1.8320	5.49E-23
11	Cald1	1.8285	3.87E-15
11	Serpine2	1.8130	4.80E-09
11	Pdgfrb	1.7938	1.34E-71
11	Csrp1	1.7635	8.30E-14
11	Col18a1	1.6632	6.73E-21
11	Hspb1	1.6543	8.27E-06
11	Cox4i2	1.6092	7.53E-51
11	Nr2f2	1.6071	2.27E-26
11	Cd248	1.6061	4.63E-37
11	Gpx3	1.5934	4.50E-07
11	Itga1	1.5682	4.32E-35
11	Actg2	1.5575	8.01E-106
11	Abcc9	1.5423	2.09E-161
11	Cygb	1.5374	6.81E-37
11	Notch3	1.5350	1.33E-211

11	Mustn1	1.5143	5.65E-83
11	Des	1.5123	1.97E-220
11	Serping1	1.5077	6.77E-30
11	Epas1	1.5052	5.75E-33
11	Prrx1	1.4958	3.17E-36
11	Ptp4a3	1.4598	3.24E-25
11	Bgn	1.4538	9.34E-17
11	Cav1	1.4392	2.15E-23
11	Crip2	1.4385	5.64E-38
11	Flt1	1.4346	3.70E-21
11	Col5a3	1.4121	4.77E-25
11	Gja4	1.4112	8.87E-273
11	Serpinh1	1.4079	2.37E-09
11	Sparc	1.3969	1.56E-10
11	Tpm4	1.3906	3.50E-09
11	Mcam	1.3704	2.50E-106
11	Gucy1a3	1.3684	0
11	Col15a1	1.3628	5.44E-31
11	Myh11	1.3606	0
11	Kcnj8	1.3070	3.39E-78
11	Vcl	1.2897	4.97E-07
11	Ednra	1.2896	1.47E-30
11	Col5a2	1.2857	3.64E-14
11	Gnb4	1.2831	1.09E-22
11	Prss23	1.2568	7.36E-17
11	Cryab	1.2565	2.59E-19
11	Marcks	1.2509	4.68E-07
11	Pdlim1	1.2480	1.36E-23
11	Rian	1.2452	2.01E-35
11	Rgs16	1.2274	1.25E-37
11	Esam	1.2031	1.96E-177
11	Tuba1a	1.1797	2.56E-05
11	Aspn	1.1658	8.70E-18
11	Rhoj	1.1518	6.03E-19
11	Rasgrp2	1.1501	1.05E-19
11	Nid1	1.1485	5.25E-17
11	Postn	1.1432	2.33E-12
11	Serpina1b	1.1393	0
11	S100a11	1.1319	1.72E-06
11	Lhfp	1.1302	5.86E-08
11	Crip1	1.1223	0.000803
11	Pdgfa	1.1202	7.51E-14
11	Mfge8	1.1141	2.22E-05
11	Cspg4	1.0968	5.20E-234
11	Prkcdbp	1.0898	6.97E-05
11	Tmem176a	1.0782	0.021998
11	Ramp1	1.0716	1.47E-06
11	Gjc1	1.0581	1.63E-19
11	Col1a1	1.0556	1.37E-06
11	Adamts2	1.0424	7.78E-20
11	Col6a1	1.0417	1.51E-08
11	Fermt2	1.0310	8.68E-17
11	Col6a2	1.0233	0.001818
11	Rgs4	1.0116	3.95E-269
11	Nbeal1	0.9997	8.03E-07
11	RP24-175N4.1	0.9983	2.85E-95
11	Col5a1	0.9906	5.38E-13
11	Myl6	0.9882	9.75E-09
11	Mprip	0.9848	1.14E-09
11	Pcolce	0.9771	9.68E-06
11	Susd2	0.9769	1.98E-132

11	Cnn3	0.9551	0.000974
11	Gcnt2	0.9421	0.002265
11	Vstm4	0.9402	4.73E-128
11	Ptrf	0.9344	0.000198
11	Pten	0.9338	3.60E-05
11	Myadm	0.9249	0.000551
11	Tnc	0.9223	0.004199
11	Dlc1	0.9178	2.09E-13
11	Tgfb2	0.9122	5.71E-17
11	Rbpms	0.9042	6.21E-11
11	Snhg18	0.8964	3.94E-09
11	Pde1a	0.8921	1.27E-83
11	Ppic	0.8918	1.46E-13
11	Actn4	0.8839	0.000982
11	Errfi1	0.8837	0.011095
11	Itgb1	0.8757	0.046262
11	Emid1	0.8753	6.75E-151
11	Adgrf5	0.8725	2.77E-60
11	Nedd4	0.8659	0.003202
11	Cacna2d1	0.8651	2.22E-42
11	Gpc4	0.8608	1.57E-20
11	Parm1	0.8454	4.62E-142
11	Olfml2b	0.8444	6.00E-18
11	Lamc1	0.8301	2.12E-07
11	Hspb2	0.8234	4.59E-25
11	Bcam	0.8232	2.39E-34
11	Ccnd2	0.8226	7.95E-06
11	Pde3a	0.8118	3.55E-81
11	Il6st	0.8099	0.000423
11	Gucy1b3	0.8053	4.54E-258
11	Prkar2b	0.8041	2.91E-12
11	Htra1	0.7992	0.000284
11	Crim1	0.7941	0.000139
11	Lmcd1	0.7933	6.96E-21
11	Sepw1	0.7819	0.001398
11	Trpc6	0.7797	5.52E-89
11	Selm	0.7759	4.55E-05
11	Tspan12	0.7750	3.33E-62
11	Loxl2	0.7735	4.35E-14
11	Tceal8	0.7716	0.004106
11	Sema6d	0.7696	5.01E-13
11	Slc12a2	0.7678	2.37E-05
11	4-Sep	0.7645	5.56E-77
11	Cyp4b1	0.7631	2.05E-18
11	Aoc3	0.7623	4.74E-51
11	Fkbp10	0.7565	6.58E-19
11	Sptbn1	0.7555	0.011377
11	Myl12a	0.7534	0.008301
11	Wnt5a	0.7530	8.46E-31
11	Calu	0.7518	0.006514
11	Apold1	0.7512	8.47E-41
11	Fkbp1a	0.7510	0.01178
11	Rock2	0.7503	0.043257
11	Dlk1	0.7501	1.77E-93
11	Ptms	0.7460	0.020332
11	C1qtnf6	0.7451	1.85E-08
11	Mgst3	0.7410	1.30E-05
11	Sulf1	0.7357	1.50E-11
11	Ano1	0.7345	1.92E-05
11	Nexn	0.7337	1.15E-17
11	Anxa6	0.7310	0.005627

11	Adamts4	0.7260	8.07E-06
11	Lix1l	0.7197	0.021178
11	Laptm4a	0.7195	0.003039
11	Tbx2	0.7130	1.68E-171
11	Msrb3	0.7090	1.53E-08
11	Myo1b	0.7088	8.74E-09
11	Adamts12	0.7076	9.98E-30
11	Ptk2	0.7066	0.003298
11	Ecscr	0.7061	1.70E-18
11	Tns1	0.7042	2.22E-08
11	Alad	0.7011	0.000681
11	Lama4	0.6991	1.18E-13
11	Map3k7cl	0.6972	1.19E-83
11	S1pr3	0.6946	1.96E-08
11	Cbfa2t3	0.6934	2.38E-28
11	Bok	0.6843	4.54E-05
11	Eng	0.6840	0.00176
11	AC166332.1	0.6749	5.53E-05
11	Tax1bp3	0.6618	0.013888
11	Fads3	0.6553	2.53E-31
11	Atp1b2	0.6496	0
11	Mxra7	0.6352	2.90E-05
11	Il1r1	0.6342	2.05E-05
11	Pla1a	0.6323	4.83E-05
11	Tgfb1i1	0.6260	2.06E-10
11	Ehd2	0.6219	0.005627
11	Gas1	0.6126	2.00E-07
11	Tmem47	0.6081	1.21E-11
11	Lrrc32	0.6014	1.15E-15
11	Jph2	0.6010	6.38E-52
11	Pear1	0.6006	0.045102
11	Osmr	0.5974	0.034948
11	Kctd17	0.5966	0.001483
11	Il34	0.5877	0.018279
11	Arhgef17	0.5860	6.98E-10
11	Parva	0.5859	4.63E-05
11	Epb41l1	0.575384223	4.69E-05
11	Ntn4	0.568279106	3.19E-13
11	Meox2	0.565120943	2.74E-49
11	Plxdc2	0.563487228	0.006743
11	Mical1	0.562885806	8.41E-17
11	Hoxb2	0.558529609	5.31E-16
11	Plekhg2	0.557994994	4.58E-05
11	Tnfrsf21	0.553232422	0.00012
11	Arhgef7	0.550775161	0.003725
11	Mkl2	0.548687075	0.021131
11	Sncg	0.547849644	4.40E-74
11	P3h3	0.545842184	0.026555
11	Snrk	0.536911179	0.001777
11	Rem1	0.526644953	2.60E-63
11	H2-M9	0.524994602	0
11	Fstl3	0.523696829	2.01E-05
11	Armcx2	0.520143514	6.56E-18
11	Itga7	0.51392715	2.25E-35
11	Tusc5	0.512805191	5.30E-295
11	Adra2a	0.507105851	3.76E-42
11	Cacna1c	0.506399407	4.12E-30
11	Ptprn	0.503205174	6.07E-11
11	Runx1t1	0.498173159	1.27E-09
11	RP24-370G18.1	0.494418018	0.000149
11	Heyl	0.49429853	1.22E-37

11	Gpx7	0.490026074	0.000188
11	Cdr2l	0.48996346	0.001525
11	Agtr1a	0.489134205	8.45E-33
11	Daam2	0.478675992	1.36E-152
11	RP24-227L14.1	0.476642428	0.003466
11	Dmd	0.470012943	1.26E-15
11	Ednrb	0.46529718	1.71E-07
11	Rasl11a	0.464654763	6.45E-09
11	Pde5a	0.454976162	9.41E-11
11	Fbn1	0.452543177	2.66E-08
11	Kcnmb1	0.451348787	6.16E-269
11	Col12a1	0.44912092	0.00602
11	Nbl1	0.448470477	0.004449
11	Afap1l2	0.447451984	1.90E-10
11	Rbpms2	0.440624791	1.75E-41
11	Fstl1	0.439150826	1.25E-11
11	Naalad2	0.433026253	5.72E-38
11	Akap12	0.42720618	7.80E-14
11	Nfatc4	0.427197913	1.07E-06
11	Hoxc9	0.425932364	4.21E-08
11	Piezo2	0.422841849	8.84E-15
11	Rab13	0.407534321	0.000175
11	Fzd4	0.406930043	1.13E-25
11	Smarca1	0.400243021	5.73E-26
11	Rasl12	0.398216137	9.51E-45
11	Nrarp	0.392555352	8.02E-05
11	Foxs1	0.391332674	3.39E-44
11	Adap2os	0.388000517	7.97E-07
11	Fam49a	0.386449684	0.006702
11	Rbms3	0.380774989	0.00708
11	Slc16a12	0.37920382	3.14E-48
11	Cxx1a	0.377753213	0.000444
11	Spry4	0.376683933	2.50E-15
11	Agrn	0.371506089	0.048567
11	Khdrbs3	0.368749945	1.46E-21
11	Arhgef25	0.362169945	0.001878
11	Pros1	0.361444011	0.037361
11	Smim1	0.346960439	0.029496
11	RP23-204I16.3	0.336323245	2.89E-21
11	AC163329.3	0.335332794	0.005112
11	Adgrl1	0.331030505	0.000535
11	Mrvi1	0.326440358	5.73E-125
11	Fhl5	0.324016372	7.03E-215

Table S2. Genes enriched (fold change >1.25) in each 4T1 tumor-derived CAF cluster compared to all other CAF clusters.

Cluster ID	*Marker Genes*	*Fold Enrichment (log2)*	*p_val_adj*
0	Sfrp4	2.099968279	2.55E-180
0	Ptx3	2.022730638	3.46E-140
0	Dpt	1.981606259	4.25E-202
0	Lbp	1.928929683	2.62E-164
0	Clec3b	1.917099792	8.04E-175
0	Pi16	1.872629543	1.10E-194
0	Saa3	1.673287277	1.64E-28
0	C3	1.605300333	2.66E-174
0	Gsn	1.59407661	3.76E-157
0	Cxcl12	1.589867221	3.89E-147
0	Ogn	1.589719189	5.81E-153

0	Col14a1	1.534241772	1.81E-173
0	Tnxb	1.529358518	2.34E-157
0	Ly6c1	1.467795454	2.50E-126
0	Efemp1	1.393211906	8.11E-158
0	C4b	1.391598347	7.33E-144
0	Lpl	1.389335507	3.34E-110
0	Igfbp4	1.343697262	2.01E-80
0	Prelp	1.311347509	4.81E-145
0	Htra3	1.299723663	3.22E-105
0	Cd34	1.284271294	2.98E-121
0	Gas6	1.2146319	3.15E-43
0	Adamts5	1.204701702	2.99E-116
0	Tnfaip6	1.179514336	3.03E-81
0	Abi3bp	1.177437016	1.11E-117
0	Prg4	1.176427529	6.21E-92
0	Apod	1.172466382	1.21E-70
0	Mmp3	1.165377738	1.06E-75
0	Dcn	1.161805916	3.55E-157
0	Chl1	1.15836595	5.23E-112
0	Itih5	1.142154606	4.11E-93
0	Serpina3n	1.137209025	1.42E-120
0	Emb	1.125583955	1.11E-125
0	Has1	1.12259428	5.67E-70
0	Sfrp2	1.117947394	2.28E-85
0	Heg1	1.109998994	9.12E-100
0	Ackr3	1.089745782	1.19E-100
0	Ugdh	1.087314051	1.76E-50
0	Scn7a	1.085829557	1.07E-124
0	Cxcl1	1.046847081	2.60E-42
0	Slfn5	1.037529056	5.36E-96
0	Klf4	1.027712738	1.20E-78
0	Cd55	1.006343482	1.43E-89
0	C1s1	0.995269254	6.26E-106
0	Gas7	0.995170071	1.07E-63
0	Ly6a	0.984061344	1.55E-102
0	Plpp3	0.965799661	8.85E-78
0	Ctla2a	0.964877399	0.0014725
0	Gda	0.95402405	1.30E-92
0	Zfp36l1	0.952751746	5.96E-81
0	Lgi2	0.935633639	6.83E-76
0	Tgfbr3	0.933916736	1.51E-99
0	Rnase4	0.931276558	1.70E-124
0	Gfpt2	0.923435038	8.45E-59
0	Il33	0.921969706	3.25E-75
0	Thbs3	0.902760579	1.80E-87
0	Ggt5	0.899806954	3.67E-88
0	Man1a	0.893987974	6.83E-74
0	Figf	0.890169681	1.29E-80
0	Pam	0.884663008	3.51E-75
0	Ace	0.881617232	5.77E-86
0	Itm2a	0.875121231	5.51E-49
0	Cygb	0.875010854	7.98E-78

0	Sema3c	0.874600725	3.75E-71
0	Trib1	0.868813268	4.52E-39
0	Sepp1	0.866004059	6.44E-75
0	Cpxm1	0.855508528	1.63E-28
0	C1s2	0.855468309	3.31E-80
0	Wisp2	0.852578156	3.30E-42
0	Cyp1b1	0.845416253	4.88E-46
0	Slc4a4	0.844990891	2.40E-82
0	Tgfbr2	0.837238518	1.75E-95
0	Scara3	0.836861387	4.28E-70
0	Sod3	0.827261071	1.92E-81
0	Nov	0.827109202	4.58E-43
0	C1ra	0.821649007	2.54E-78
0	Il11ra1	0.801987346	1.27E-74
0	Gstm1	0.791739291	6.83E-61
0	Gxylt2	0.789257454	6.73E-70
0	Gpx3	0.784978419	6.12E-56
0	Creb5	0.782628241	3.10E-53
0	Pdgfra	0.772557274	2.96E-59
0	Nfib	0.769089016	8.26E-65
0	Flrt2	0.759917927	3.21E-52
0	Fndc1	0.755072484	1.22E-57
0	Slc43a3	0.747634609	1.98E-67
0	Dpp4	0.747408712	7.26E-70
0	C1rb	0.747241786	7.64E-60
0	Spon2	0.743723718	1.92E-51
0	Zbtb20	0.741734235	1.51E-72
0	Nfia	0.740075312	1.26E-59
0	Tppp3	0.739159449	4.66E-43
0	Fbln1	0.738506079	9.06E-40
0	Col6a5	0.737381246	1.25E-32
0	Svep1	0.736613015	8.37E-46
0	Procr	0.736449169	1.01E-19
0	Sgk1	0.733767864	1.49E-25
0	Pdpn	0.730155968	1.69E-72
0	Hmox1	0.726162647	1.20E-16
0	Ltbp4	0.723421848	4.22E-49
0	Igf1	0.718012895	7.88E-44
0	Galnt16	0.714195688	2.65E-73
0	Mt2	0.71164503	1.08E-55
0	Emilin2	0.709398236	2.75E-52
0	Ccl7	0.708445216	2.81E-46
0	Ltc4s	0.703254502	3.13E-36
0	Fgl2	0.702841274	2.67E-24
0	Adamts1	0.70047837	2.88E-36
0	Bag3	0.699515282	1.29E-31
0	Ltbp1	0.693912899	9.96E-57
0	Cyr61	0.692811406	9.62E-44
0	Cmah	0.68773054	7.08E-50
0	Uap1	0.687449191	3.68E-27
0	Ifi27l2a	0.684263009	2.86E-30
0	Ddr2	0.679539848	7.02E-45

0	Fth1.1	0.672449134	4.92E-108
0	Scara5	0.671319817	3.65E-67
0	Ccl2	0.67067168	9.69E-37
0	Cyb5a	0.666733886	1.32E-48
0	Adamts15	0.666103931	2.83E-42
0	Adgrd1	0.663150906	3.45E-52
0	Ptp4a1	0.66138787	6.41E-40
0	Pcolce2	0.653856187	9.25E-34
0	Tiparp	0.652566867	1.98E-17
0	Mgst1	0.647473469	1.05E-59
0	Sned1	0.644406507	5.07E-37
0	Sash1	0.640975147	1.57E-34
0	Entpd2	0.640231199	1.01E-54
0	Il6st	0.639754918	3.89E-40
0	Mmp2	0.638314119	1.44E-69
0	Marcks	0.637821046	2.35E-42
0	Ahnak	0.637719901	5.76E-68
0	Celf2	0.634253869	1.20E-47
0	Fgf7	0.633198735	3.40E-47
0	Daglb	0.632180552	6.67E-32
0	Pamr1	0.629786853	5.26E-62
0	Nid1	0.6219775	1.78E-47
0	Bmper	0.619470495	5.94E-47
0	Steap3	0.619287699	1.51E-47
0	Podn	0.619060637	1.07E-35
0	Cadm3	0.617672992	1.92E-45
0	Anxa3	0.617384206	1.05E-19
0	Il1rl1	0.612023419	6.24E-06
0	Nfkb1	0.606833012	1.75E-20
0	Ptrf	0.605964783	2.86E-34
0	Cdh13	0.602870268	6.96E-41
0	Ppp1r2	0.600827702	1.00E-34
0	Lgmn	0.600765582	1.08E-39
0	Tmeff2	0.596668731	2.76E-56
0	Man2a1	0.588106329	1.50E-33
0	Dusp1	0.587953926	1.17E-16
0	Il6	0.58729896	1.74E-17
0	Ier5	0.583676412	6.19E-25
0	RP24-291M22.2	0.580624956	1.50E-41
0	Fstl1	0.575730966	5.54E-75
0	Vasn	0.569336619	1.08E-24
0	Clip4	0.566725306	1.69E-46
0	Arsi	0.564791502	6.52E-50
0	Ablim1	0.564039479	6.03E-41
0	Jak1	0.562462123	9.75E-44
0	Lpar1	0.56165053	3.77E-38
0	Mat2a	0.558508457	4.53E-25
0	Irak3	0.557624885	1.76E-42
0	Ifrd1	0.553197678	6.98E-26
0	F3	0.552022622	3.18E-18
0	Ngf	0.548440074	4.03E-39
0	Kdm6b	0.548038763	1.81E-18

0	Maff	0.543826032	1.37E-14
0	Serpinb6a	0.543429567	1.08E-41
0	RP23-63A19.3	0.543342897	3.99E-42
0	Dpysl3	0.541499691	6.09E-38
0	Klf9	0.528465853	7.72E-23
0	Steap4	0.524621731	4.08E-47
0	Dapk1	0.524325105	2.54E-42
0	Slc16a2	0.522317173	8.90E-34
0	Sirpa	0.520451185	3.92E-27
0	S100a16	0.518235576	3.12E-25
0	Igfbp6	0.517443041	3.57E-34
0	Ugcg	0.516428897	1.61E-35
0	Basp1	0.51495128	1.43E-19
0	Pnp	0.508248711	6.39E-16
0	Atf3	0.505553841	4.43E-11
0	St3gal1	0.504056359	7.08E-32
0	Serping1	0.503142133	9.36E-61
0	Ifi205	0.500590125	1.49E-35
0	Srxn1	0.495827754	8.02E-14
0	Fxyd1	0.493689277	2.40E-26
0	S100a10	0.492851264	2.73E-26
0	Lrrn4cl	0.491662749	1.21E-37
0	Lum	0.489841346	2.16E-50
0	Fosb	0.489722151	8.36E-22
0	Lysmd2	0.489327073	8.21E-28
0	Nr4a3	0.488540977	3.26E-07
0	Mycbp2	0.487964634	4.34E-28
0	Col3a1	0.486703401	1.06E-66
0	Ebf2	0.48665158	1.62E-17
0	Dcbld2	0.485454935	8.17E-21
0	Ramp2	0.484522925	2.12E-37
0	Nr4a1	0.48146252	1.51E-07
0	Dpep1	0.480113366	1.96E-27
0	Jund	0.479315572	3.95E-42
0	S100a13	0.477074202	4.40E-31
0	Nsg1	0.476875252	1.38E-23
0	Svil	0.47679935	1.36E-18
0	Cebpd	0.476376582	1.53E-20
0	Pim1	0.475801919	3.28E-19
0	Mcc	0.473984533	1.43E-26
0	Mndal	0.472648893	1.02E-13
0	Clic4	0.462115519	2.52E-24
0	Fam102b	0.461379915	1.89E-22
0	Yod1	0.459953117	0.0001778
0	Cd44	0.459920366	1.17E-13
0	Timp2	0.458974732	5.50E-46
0	Islr	0.458423865	2.14E-18
0	Flnc	0.455572692	2.91E-07
0	Adora2b	0.453502971	6.78E-22
0	Hk2	0.452691547	3.80E-07
0	Csrnp1	0.450659314	8.56E-14
0	Ext1	0.450071644	2.24E-14

0	Phlda1	0.447848949	7.21E-09
0	Raph1	0.446793272	4.08E-10
0	Pcsk6	0.445204342	3.98E-33
0	Smoc2	0.444992735	4.92E-08
0	Ctsl	0.444257095	4.29E-53
0	Epdr1	0.44353172	3.94E-30
0	Sntb2	0.441843095	3.55E-23
0	Zfp36l2	0.439428421	8.84E-11
0	Mesdc1	0.438306064	2.77E-18
0	Dbi	0.437920727	5.67E-18
0	Tsc22d2	0.437346935	7.62E-11
0	Midn	0.437233475	6.21E-14
0	CAAA01194877.2	0.437045449	5.79E-11
0	Lrrc17	0.436085956	1.04E-18
0	Il6ra	0.435012331	5.02E-26
0	Tshz2	0.43369458	5.95E-20
0	Cpq	0.433337157	1.20E-20
0	Add3	0.43321993	1.36E-18
0	Baiap2	0.42828915	1.80E-13
0	Ifi202b	0.426430488	1.37E-12
0	Prr13	0.426408952	2.56E-15
0	Ebf1	0.424032398	2.16E-17
0	Dst	0.424001487	8.93E-18
0	Frmd6	0.422133364	1.89E-17
0	Cd302	0.42112751	1.60E-17
0	Arhgap29	0.420954253	1.08E-15
0	Prnp	0.4205739	8.30E-17
0	Gpc3	0.419098933	8.38E-16
0	RP23-159L13.1	0.418062086	3.72E-35
0	Ccnl1	0.417028473	4.71E-20
0	Azin1	0.416925657	1.48E-09
0	Ccl11	0.416688147	9.99E-26
0	Adm	0.415690423	4.53E-09
0	Spry2	0.413704345	5.74E-22
0	Ndrg1	0.410906711	1.43E-09
0	Lama4	0.4082198	1.73E-18
0	Kitl	0.407742689	4.44E-20
0	Psap	0.40345187	1.11E-40
0	Ugp2	0.403185616	1.72E-09
0	Egfr	0.402218364	2.29E-16
0	Cdkn1c	0.401821015	4.60E-20
0	Bicc1	0.401696909	9.72E-27
0	Pde8a	0.399145855	7.10E-25
0	Errfi1	0.398685527	1.53E-10
0	Ces2g	0.395204082	1.64E-33
0	Duoxa1	0.394874831	3.13E-18
0	Rhou	0.394795597	2.14E-12
0	Samd4b	0.391324836	2.56E-13
0	Dnajb4	0.388274395	2.41E-12
0	Cebpb	0.384608612	4.88E-15
0	Osr1	0.383437835	1.09E-20
0	Nfix	0.381818728	1.19E-21

0	Grb10	0.377838085	4.68E-11
0	Adamtsl1	0.374869189	1.25E-12
0	Osr2	0.374559666	1.32E-08
0	Dnajb1	0.373468381	3.45E-08
0	Cd47	0.372637149	1.61E-21
0	Fyn	0.371469328	2.27E-11
0	Psd3	0.371075167	1.58E-10
0	Sema3b	0.37019205	1.75E-21
0	Gadd45g	0.369877637	1.44E-09
0	Il17ra	0.368196921	1.86E-12
0	Plod2	0.367537142	1.47E-12
0	Csf1	0.367100833	7.47E-17
0	Cd248	0.366834221	4.89E-11
0	Hes1	0.365836894	7.58E-07
0	Nfasc	0.364801973	1.27E-25
0	Nfkbiz	0.364625578	8.75E-09
0	Ecm1	0.363973879	1.68E-09
0	Dram1	0.363092988	2.22E-12
0	Il1r1	0.362828317	2.02E-09
0	Reep3	0.361876611	5.71E-08
0	Vcan	0.361866824	6.52E-17
0	Rarres2	0.359230849	5.44E-28
0	Nav1	0.357696169	5.28E-11
0	Med13	0.357290756	8.23E-09
0	Rras2	0.352606584	0.0028139
0	Prss23	0.350887097	1.05E-09
0	Wbscr17	0.350199995	1.49E-16
0	RP23-322E23.1	0.348570297	4.27E-08
0	Ptgis	0.347815318	1.08E-15
0	Icam1	0.347793304	6.88E-06
0	Hsd11b1	0.347467729	1.83E-07
0	Chd1	0.347291435	5.18E-05
0	Vcam1	0.346073367	4.33E-07
0	Aig1	0.345210909	0.0093625
0	Gns	0.34503606	8.05E-12
0	Plagl1	0.344641016	1.53E-05
0	Fam129a	0.344190018	5.29E-19
0	Glul	0.343795058	0.0166957
0	Cyp7b1	0.343244201	0.0018505
0	Sdk1	0.34140222	3.26E-23
0	Lrp6	0.341059623	4.04E-06
0	Smad7	0.340302883	2.45E-05
0	Zfp703	0.339164816	1.69E-08
0	Rock2	0.338389078	9.63E-08
0	Adgra2	0.336864184	1.74E-06
0	Ctps	0.336673947	1.13E-08
0	Nbl1	0.336555429	8.10E-10
0	Srgn	0.335697109	5.34E-18
0	Fosl2	0.335211236	0.0035829
0	Cd164	0.33357189	6.25E-08
0	Vwa5a	0.332101704	2.13E-08
0	Pkdcc	0.330694056	1.81E-13

0	Sulf2	0.325622549	5.98E-07
0	Blvrb	0.324036899	1.20E-10
0	Clmp	0.323268972	2.65E-10
1	Igfbp3	2.240311671	6.76E-139
1	Tagln	1.887004618	1.04E-143
1	Acta2	1.84503353	3.74E-141
1	Tnc	1.786989747	2.42E-162
1	Inhba	1.718086029	1.63E-157
1	Col8a1	1.576362565	3.64E-183
1	Lrrc15	1.49118478	2.78E-150
1	Cxcl9	1.437469026	1.90E-40
1	Tpm2	1.363079591	3.32E-126
1	Col7a1	1.346861492	4.30E-84
1	Pgf	1.344979927	1.42E-84
1	Ctgf	1.299594308	8.45E-73
1	Grem1	1.251741209	1.08E-50
1	Sparcl1	1.206156917	3.31E-47
1	Tmem119	1.149529676	1.39E-108
1	Cxcl14	1.140848044	1.92E-58
1	Myl9	1.131581957	2.70E-80
1	Thbs2	1.077212268	1.32E-100
1	Col15a1	1.056532889	4.44E-94
1	Fabp5	1.01624463	2.87E-26
1	Col12a1	1.012686684	2.71E-106
1	Tpm1	0.991145063	1.65E-63
1	Iigp1	0.967039952	3.27E-46
1	Bpgm	0.96486156	3.42E-87
1	Actg2	0.956560847	3.66E-24
1	Csrp1	0.92831851	5.91E-82
1	Gng11	0.926770228	1.30E-83
1	Cpxm2	0.915457275	1.44E-95
1	Olfml3	0.897400988	1.25E-86
1	Vegfa	0.89548381	1.55E-58
1	Tgfb1	0.894187458	2.35E-94
1	Spon1	0.882327126	4.47E-105
1	Ndufa4l2	0.856103444	2.25E-50
1	Rgs16	0.854243799	5.17E-69
1	Pgk1	0.847603694	5.28E-67
1	Pkm	0.835285779	9.16E-102
1	Igfbp7	0.824459182	1.67E-105
1	Spp1	0.800827204	1.55E-86
1	Dkk3	0.785748792	6.41E-61
1	C1qtnf3	0.78350849	4.10E-37
1	Ptprn	0.780706925	1.62E-97
1	Cald1	0.774830958	7.22E-128
1	Ctsc	0.774825327	1.09E-68
1	Eno1	0.765216981	1.32E-59
1	Slc16a3	0.761391748	1.57E-67
1	Mmp9	0.754563397	2.82E-38
1	Cnn2	0.754458626	1.24E-74
1	Crlf1	0.753734297	2.26E-45
1	Wnt5a	0.752387582	1.78E-72

1	Serpine2	0.751858919	1.15E-13
1	Tpi1	0.747892534	8.26E-56
1	Col6a3	0.747269247	1.98E-57
1	Isg15	0.743186544	9.69E-41
1	Mfge8	0.74181333	1.36E-78
1	P4ha2	0.741155292	1.71E-40
1	Pdgfrb	0.740679065	1.80E-65
1	Fbxo32	0.739910869	8.56E-50
1	Loxl3	0.738160411	6.89E-65
1	Hs6st2	0.734286085	1.44E-74
1	Angptl2	0.728480797	1.24E-35
1	Col5a2	0.728037962	1.06E-117
1	Cthrc1	0.724822346	6.32E-33
1	Pmepa1	0.722111897	5.47E-102
1	Mical2	0.720910805	5.85E-76
1	Stra6	0.712419907	4.83E-97
1	Ltbp2	0.707427524	7.93E-46
1	Col1a1	0.705426259	7.71E-81
1	Ero1l	0.700064727	3.27E-37
1	Cspg4	0.6942687	8.64E-76
1	Pgam1	0.690720097	1.19E-68
1	Myh9	0.690124287	7.20E-72
1	Rbp1	0.676219071	1.29E-59
1	Hbegf	0.673159033	1.16E-40
1	Ptk7	0.664861785	8.44E-76
1	Ncam1	0.664719571	8.25E-75
1	Cdc42bpa	0.663088842	2.33E-55
1	Nrp2	0.660014781	1.80E-49
1	Tmsb4x	0.653580417	2.04E-30
1	Lgalsl	0.645417546	1.01E-52
1	Itgb1	0.632202043	8.27E-83
1	Wisp1	0.630191744	5.46E-59
1	Cnn3	0.629332771	3.17E-59
1	Gapdh	0.62915953	9.40E-53
1	Pdgfrl	0.622634513	5.24E-54
1	Fam198b	0.617972801	1.88E-70
1	Bnip3	0.614951097	1.26E-38
1	Gbp4	0.611503815	2.60E-25
1	Unc5b	0.610671587	6.98E-67
1	Fermt2	0.609414694	3.33E-62
1	Mgp	0.607113509	0.0053464
1	Fam162a	0.604866224	9.12E-24
1	Thbs1	0.602958536	4.00E-25
1	Kremen1	0.600272371	2.30E-59
1	Pdgfa	0.600130259	1.15E-42
1	Slfn2	0.599318858	1.26E-56
1	Ddit4	0.597993958	1.15E-41
1	Aldoa	0.597884757	8.13E-59
1	Slc2a1	0.596185711	1.41E-50
1	Igtp	0.594037485	2.08E-41
1	Pla2g7	0.59258357	4.08E-46
1	Tnfrsf23	0.571660872	3.56E-43

1	Irf7	0.570989185	9.67E-29
1	Cdh11	0.569498678	6.03E-45
1	Esyt2	0.569199757	1.83E-39
1	Piezo2	0.56100549	2.90E-65
1	Ankrd44	0.559573644	1.38E-42
1	Nrep	0.558701717	1.55E-42
1	Ass1	0.558578359	2.18E-40
1	AC130827.2	0.554671687	4.26E-47
1	Kif26b	0.5517048	9.89E-55
1	Bsg	0.551382799	4.24E-28
1	Myl6	0.550794593	2.86E-54
1	RP23-8J15.3	0.550166174	2.50E-26
1	Adam19	0.549747131	3.03E-42
1	Sema7a	0.549343253	1.51E-56
1	Tpm4	0.548958422	9.82E-60
1	Hilpda	0.540420031	1.41E-16
1	Adamts4	0.538602725	1.25E-42
1	Postn	0.537626609	1.89E-35
1	RP23-310J6.1	0.5334485	2.30E-22
1	Fmod	0.531965188	9.29E-20
1	Chst11	0.53089636	5.32E-43
1	Egln1	0.528704687	2.18E-34
1	P4ha1	0.525808713	1.06E-39
1	Tln2	0.524046266	1.00E-40
1	Nudt4	0.523881002	2.81E-34
1	Actn1	0.523612742	1.26E-43
1	Pfkl	0.522738962	1.25E-42
1	Cryab	0.517048681	1.98E-12
1	Tspan3	0.513358043	3.07E-43
1	Oaf	0.511673894	4.80E-40
1	Nkd2	0.509822008	1.36E-52
1	Kctd11	0.508296322	1.00E-27
1	Pappa	0.507644969	8.76E-36
1	Pqlc1	0.50638474	1.46E-40
1	Gbp7	0.504590175	1.45E-27
1	Tgfb3	0.503335289	4.30E-42
1	Flna	0.50271184	5.54E-34
1	Uck2	0.500843986	6.66E-43
1	Ccnd2	0.499455955	3.27E-28
1	Plod1	0.496738511	2.96E-33
1	Slc20a1	0.491874262	6.08E-18
1	Myl12a	0.491814793	3.76E-42
1	RP24-286N17.2	0.490433528	2.69E-45
1	Srpx2	0.488189515	5.55E-36
1	Tfpi2	0.487763542	7.21E-31
1	Akr1b8	0.485366504	4.44E-27
1	Tln1	0.480884406	6.72E-35
1	Mif	0.478049099	3.22E-37
1	Palld	0.474477753	3.43E-32
1	Nxn	0.470831798	2.99E-41
1	Furin	0.469848918	4.17E-28
1	Tgfb2	0.46786874	4.92E-23

1	Irf1	0.467738032	1.26E-19
1	Dhrs3	0.464271209	5.29E-37
1	H2-Q7	0.463573993	4.31E-26
1	Higd1a	0.461410938	7.64E-25
1	Fstl3	0.459702681	3.17E-36
1	Eva1b	0.457308308	3.45E-29
1	Ostf1	0.455914746	8.37E-38
1	Serpine1	0.454693311	2.77E-16
1	Rnf149	0.453618138	6.20E-40
1	Soat1	0.450363529	6.29E-34
1	Tmem86a	0.450318266	1.58E-33
1	Ldha	0.45002771	3.64E-25
1	Gpi1	0.449481682	1.94E-25
1	Mast4	0.448441387	1.41E-24
1	Zbp1	0.439361337	1.35E-21
1	Psmb8	0.436485965	9.83E-22
1	Prokr2	0.433792474	1.71E-54
1	Dok1	0.431908351	2.76E-35
1	P3h1	0.431347013	8.54E-30
1	Tcf4	0.429852367	1.74E-29
1	Itgb5	0.429845606	1.51E-26
1	Emilin1	0.429578219	6.14E-26
1	Actb	0.428566985	1.07E-25
1	Timp3	0.42340125	2.87E-21
1	Tmem2	0.422160872	4.59E-25
1	Ak1	0.416060843	1.71E-40
1	Fam101b	0.413914687	9.16E-34
1	Prdm1	0.410924025	2.13E-27
1	Gbp2	0.408714691	2.18E-09
1	Fkbp10	0.408009139	2.71E-29
1	Runx1	0.407515891	2.51E-28
1	Picalm	0.407398632	7.60E-22
1	Stat1	0.406702846	2.38E-22
1	Gbp2b	0.40598585	3.37E-15
1	RP23-285C18.2	0.40588443	1.47E-19
1	Prkar1a	0.405763404	6.00E-31
1	Serpina3g	0.405177131	2.22E-14
1	Fam46a	0.402867565	1.82E-14
1	Iqgap1	0.402459171	1.22E-22
1	4-Sep	0.401774395	1.38E-27
1	Tmem45a	0.397790797	2.60E-16
1	Cdc42ep3	0.397509026	1.74E-33
1	H2-K1	0.396492837	1.17E-23
1	Bace1	0.395016839	2.77E-31
1	Ier2	0.394495768	2.23E-10
1	H2-Q6	0.39233569	2.37E-17
1	Loxl2	0.38995085	5.00E-20
1	Rgcc	0.387055325	6.11E-30
1	Crispld2	0.384922618	2.24E-16
1	Tes	0.383411016	5.76E-31
1	RP23-8J15.4	0.381538712	6.85E-06
1	Abracl	0.380405645	5.59E-19

1	Rcn3	0.379446342	1.33E-28
1	Pgp	0.37757694	1.10E-19
1	Lgals1	0.375884914	4.02E-40
1	Tmeff1	0.375565043	2.87E-34
1	Ilk	0.375248455	2.17E-25
1	Dusp4	0.374728185	8.84E-27
1	Tap1	0.372045368	1.27E-21
1	Rai14	0.37138301	4.38E-22
1	RP23-8J15.2	0.370908697	0.0009988
1	Pkig	0.370531544	5.00E-24
1	Rasl11a	0.369446457	2.91E-12
1	Zfpm1	0.367880006	1.16E-30
1	Moxd1	0.366852497	1.26E-33
1	Myo1e	0.366833126	4.27E-30
1	Adamts6	0.366753008	2.30E-35
1	Dad1	0.366355913	3.96E-28
1	Nrip1	0.36550794	1.32E-12
1	Olfr1033	0.36504169	5.22E-08
1	Colec12	0.363365662	2.42E-26
1	Myo10	0.362208948	9.79E-24
1	Irgm1	0.360976075	8.84E-36
1	Bst2	0.360618199	4.07E-11
1	Scarf2	0.359988599	1.61E-21
1	H2-D1	0.358176433	3.86E-14
1	Vdac2	0.357800078	9.77E-26
1	Adamts12	0.356974811	1.43E-18
1	Cyp51	0.356545755	1.96E-23
1	9-Sep	0.3530132	8.51E-25
1	Actn4	0.350144826	1.09E-18
1	Col1a2	0.34849004	5.77E-30
1	Capg	0.347418497	2.75E-24
1	Tns3	0.345892461	1.00E-26
1	Pold4	0.345247776	5.56E-22
1	Gadd45b	0.34463135	1.93E-12
1	Flnb	0.344356926	4.03E-16
1	C2	0.344327332	1.71E-13
1	P3h3	0.344155966	4.43E-21
1	AC162528.2	0.343839495	9.29E-37
1	Pdrg1	0.343271149	2.62E-22
1	Mdk	0.343161479	1.43E-14
1	Ctxn1	0.342207897	1.60E-38
1	Msn	0.341914589	3.43E-22
1	Ercc1	0.341579084	1.13E-23
1	Pstpip1	0.338966273	5.75E-40
1	Pla2g16	0.336922093	7.55E-19
1	Foxs1	0.332965564	9.41E-35
1	Bgn	0.332485005	9.80E-30
1	Mtch1	0.332041787	1.28E-22
1	Tnfaip2	0.329981041	1.53E-12
1	Ifi44	0.328605272	9.55E-21
1	Ifi47	0.328449069	1.94E-11
1	B2m	0.328115223	3.24E-14

1	Bhlhe41	0.328055231	3.29E-29
1	Tsc22d1	0.32803242	9.07E-15
1	Htr2a	0.327749865	2.90E-30
1	Sparc	0.327555806	5.80E-30
1	Fam78b	0.327065586	1.33E-28
1	Ccdc80	0.326091085	4.94E-17
1	RP23-189B9.3	0.32328181	4.36E-11
2	Ube2c	1.748479306	2.47E-165
2	Top2a	1.627197322	2.74E-175
2	H2afz	1.445885398	1.75E-75
2	Stmn1	1.431418556	2.65E-142
2	Mki67	1.392105326	1.08E-187
2	Birc5	1.370263761	9.10E-174
2	Prc1	1.309871377	2.36E-176
2	Pclaf	1.273905766	4.53E-195
2	Cenpf	1.269050412	7.62E-165
2	Cdk1	1.252991886	1.21E-147
2	Smc2	1.238154262	3.24E-141
2	Cenpa	1.186437212	2.08E-118
2	Tubb5	1.181989305	1.39E-65
2	Hmgb2	1.155877127	1.85E-100
2	Tpx2	1.13912155	1.50E-181
2	Smc4	1.130249142	6.46E-83
2	Cks2	1.077129584	1.84E-100
2	Cdca8	1.064796961	2.85E-192
2	RP23-387F9.3	1.039092568	6.64E-60
2	Cdc20	1.026470859	1.03E-154
2	Tuba1b	0.984387564	6.69E-52
2	Nucks1	0.970399158	3.13E-52
2	Tyms	0.958073807	2.30E-100
2	Ube2s	0.958071621	2.69E-45
2	Hmmr	0.920610905	7.75E-160
2	Cenpe	0.918637203	2.63E-149
2	Ccna2	0.908107401	1.84E-210
2	Tmpo	0.894385332	1.56E-82
2	Hist1h1b	0.874348774	1.01E-114
2	Pttg1	0.87094609	3.55E-101
2	Tubb4b	0.870115081	1.49E-43
2	Rrm1	0.86923936	7.55E-76
2	Ccnb2	0.868863869	5.02E-112
2	Ran	0.856003844	1.08E-49
2	H2afx	0.848953549	9.42E-65
2	Cks1b	0.835675147	2.16E-55
2	Racgap1	0.833614954	6.01E-173
2	Rrm2	0.833440363	1.72E-160
2	Ccdc34	0.824634566	2.04E-67
2	Spc24	0.821228585	1.67E-121
2	Tubb6	0.800843325	1.42E-39
2	Tk1	0.799745486	3.91E-139
2	Dek	0.7954834	2.06E-46
2	Ckap2l	0.782711241	2.44E-160
2	Anln	0.773625928	1.36E-138

2	Arl6ip1	0.765903846	1.07E-21
2	Tm4sf1	0.753274687	1.66E-14
2	Hn1	0.74978947	1.12E-28
2	Plk1	0.744868173	3.09E-149
2	Cdca3	0.742146723	1.99E-122
2	Ccnb1	0.732768521	9.68E-152
2	Nusap1	0.701597352	3.36E-137
2	Ranbp1	0.699461225	3.83E-33
2	Pbk	0.6887089	4.68E-172
2	Ckap4	0.684236788	3.61E-38
2	Ptma	0.679266892	1.86E-44
2	Tacc3	0.67706631	6.26E-115
2	Slfn9	0.664075699	4.68E-83
2	Cdkn2d	0.657331898	4.83E-37
2	Dut	0.655733848	1.75E-34
2	Spc25	0.650634905	3.49E-145
2	Rangap1	0.646070426	1.95E-51
2	Snrpd1	0.643756765	8.70E-33
2	H2afv	0.643718957	1.61E-26
2	Ssrp1	0.632965735	2.48E-33
2	Nasp	0.623092886	1.05E-34
2	Rad21	0.605672197	1.72E-34
2	Lig1	0.605273394	7.06E-51
2	Casc5	0.602312285	1.11E-144
2	Mcm6	0.596650092	7.10E-74
2	Lmnb1	0.595647649	3.04E-75
2	Kif22	0.594917758	1.48E-118
2	Diaph3	0.591476137	4.31E-111
2	Fam111a	0.583826971	3.12E-77
2	Anp32b	0.582678801	1.04E-26
2	Usp1	0.580774412	2.61E-42
2	Incenp	0.575234715	5.19E-105
2	Knstrn	0.573688069	1.60E-101
2	Atad2	0.569619767	2.67E-41
2	Fam64a	0.568676084	4.58E-142
2	Cxcl10	0.56722303	1.01E-12
2	Dnajc9	0.561777311	1.04E-75
2	Cxcl9	0.557382549	0.0004861
2	Bub3	0.552020367	4.37E-24
2	Dhfr	0.550277536	3.64E-89
2	Hnrnpab	0.546217338	2.55E-23
2	Cenpm	0.545546905	6.55E-101
2	Kif11	0.533078522	8.55E-140
2	Alyref	0.519619485	1.54E-24
2	Smc1a	0.519202772	2.00E-22
2	Mad2l1	0.517500269	6.12E-93
2	Plk4	0.514441042	1.36E-70
2	Snrpe	0.512648375	6.77E-24
2	Mcm3	0.512289354	8.26E-53
2	Nme1	0.512265197	1.86E-17
2	Kif23	0.510014485	1.04E-111
2	Dbf4	0.49866179	1.16E-76

2	Mcm5	0.497988992	4.70E-67
2	Ywhah	0.49724296	3.06E-17
2	Uhrf1	0.494804206	4.46E-73
2	Psip1	0.491208511	2.86E-24
2	Anp32e	0.48679698	5.33E-30
2	Cdkn3	0.48190755	5.39E-76
2	Srsf3	0.481842059	1.55E-17
2	Hmgn2	0.481572602	1.49E-39
2	Cenpw	0.47863396	1.49E-75
2	Lsm2	0.477735252	6.59E-30
2	Gmnn	0.474501237	4.88E-69
2	Cbx3	0.474398784	1.21E-19
2	Rfc5	0.473480348	2.15E-64
2	Hjurp	0.473352356	4.71E-30
2	Serbp1	0.465099672	1.92E-18
2	Npm1	0.464478734	6.55E-14
2	Dtymk	0.463949044	3.51E-22
2	Nuf2	0.463176774	3.02E-130
2	Nap1l1	0.46080447	3.25E-12
2	Kif20b	0.459877288	4.21E-100
2	Rbm3	0.459858821	2.10E-22
2	Bub1b	0.458022296	4.00E-108
2	Tipin	0.456223669	1.43E-20
2	Arhgap11a	0.455011407	2.05E-115
2	Ncapg2	0.454841953	1.79E-58
2	Exosc8	0.454444612	1.58E-29
2	Aurka	0.453584235	1.96E-98
2	Idh2	0.453261994	4.58E-19
2	Mcm7	0.452198791	1.15E-39
2	Hnrnpa1	0.4515517	1.15E-14
2	Mcm2	0.447877006	5.00E-36
2	Sgol2a	0.447271759	1.72E-101
2	Lsm6	0.445178489	1.35E-25
2	Tagln2	0.443964672	5.85E-15
2	Clspn	0.440725249	6.49E-90
2	Aurkb	0.440576354	7.60E-110
2	Gins2	0.439462257	8.71E-54
2	RP23-185B14.1	0.438551262	2.14E-92
2	Sae1	0.437829686	2.91E-18
2	Ncl	0.43582957	9.56E-14
2	Cbx5	0.433896872	1.29E-20
2	Ddx39	0.433633698	6.78E-21
2	Lsm8	0.432749561	9.64E-25
2	Ckap2	0.426961734	9.20E-101
2	Topbp1	0.424356004	4.35E-43
2	Hint1	0.424313669	3.44E-18
2	Pmf1	0.421171698	1.16E-61
2	Aspm	0.421079481	1.82E-113
2	Slc25a5	0.420599352	5.48E-11
2	Melk	0.420492	3.89E-127
2	Dok1	0.415991141	7.40E-09
2	Ndc1	0.415181436	1.69E-62

2	Lrrc59	0.414423697	7.15E-13
2	Rpa2	0.41430223	4.14E-45
2	Calm2	0.410219259	2.72E-14
2	Kif4	0.409628453	9.37E-97
2	Nsd2	0.409454669	1.62E-23
2	Rcc1	0.408774679	7.10E-46
2	Hdgf	0.408329114	3.40E-13
2	Dlgap5	0.408272627	2.24E-108
2	Kif2c	0.407438986	2.37E-102
2	Cmc2	0.404279339	3.55E-25
2	Lsm3	0.403808539	1.07E-21
2	Ppia	0.40340766	2.31E-16
2	AC155158.1	0.403393329	1.51E-12
2	Snrpa1	0.402991228	1.24E-16
2	Ybx1	0.402381556	6.23E-18
2	Csrp2	0.40221687	7.79E-08
2	Bub1	0.401138125	1.66E-97
2	Nsmce4a	0.39999467	2.41E-21
2	Pdap1	0.399542682	2.46E-10
2	Cep55	0.398158983	4.12E-100
2	Kpnb1	0.398050822	6.45E-14
2	Snrpb	0.397616815	1.05E-16
2	Lyar	0.395394135	1.18E-18
2	Nt5dc2	0.391401088	1.42E-16
2	Fst	0.391381332	8.27E-09
2	Tubb2b	0.390917872	1.62E-11
2	Rad51	0.390361052	8.51E-72
2	Cdc45	0.389991595	2.37E-45
2	Hells	0.38702523	3.89E-38
2	Hat1	0.386995527	4.20E-30
2	Mxd3	0.386231646	1.84E-98
2	Srsf2	0.385063636	5.78E-13
2	Hmgn1	0.384970278	8.23E-14
2	Ccl2	0.38417039	0.0277353
2	Shcbp1	0.383527405	3.66E-106
2	Mcm4	0.382931771	1.06E-28
2	Arpp19	0.380011951	1.04E-13
2	Phf5a	0.379560285	1.43E-10
2	Smc3	0.378560338	3.89E-14
2	Tpr	0.377710414	3.31E-13
2	Trip13	0.377525871	1.17E-73
2	Nsmce1	0.375938964	2.51E-15
2	Ezh2	0.375859044	2.35E-29
2	Ncapg	0.375335435	2.69E-96
2	Nop58	0.375124339	8.05E-13
2	Kif20a	0.375038842	2.76E-80
2	Hnrnpf	0.373606741	1.21E-13
2	Ptms	0.372983947	2.71E-07
2	Ckap5	0.372958541	7.46E-25
2	Nhp2	0.372005443	2.09E-11
2	Hnrnpd	0.371819767	1.96E-13
2	Cdkn2c	0.370592032	6.16E-32

2	Reep5	0.369131148	2.72E-12
2	Rad51ap1	0.36908726	4.43E-103
2	Foxm1	0.36602037	6.16E-82
2	Siva1	0.365928878	4.72E-07
2	Sumo2	0.365063973	3.05E-12
2	Tfdp1	0.364179661	1.25E-18
2	H2afy	0.364027846	8.87E-09
2	Naa50	0.363945462	1.22E-11
2	Tcof1	0.363657445	2.77E-21
2	Mthfd2	0.363231149	2.65E-17
2	Anapc11	0.362300667	1.19E-10
2	Ubtf	0.362136667	4.59E-07
2	Cbx1	0.358967748	4.45E-13
2	Actg1	0.35696293	7.19E-10
2	Rfc2	0.356920103	8.84E-19
2	Cenph	0.354960228	8.27E-90
2	Eif5a	0.353521302	4.50E-06
2	Dctpp1	0.352661724	2.79E-17
2	Gas2l3	0.352148446	9.65E-21
2	Hnrnpa2b1	0.350888504	1.08E-10
2	Nono	0.3490272	3.01E-11
2	Nans	0.34819424	7.83E-11
2	Alad	0.344922917	6.81E-12
2	Vim	0.344158052	3.21E-09
2	Set	0.344110715	3.93E-09
2	Kif15	0.34241708	1.77E-101
2	Pin1	0.342253862	1.22E-08
2	Sf3b5	0.34203946	3.16E-10
2	Smchd1	0.341603779	2.61E-14
2	Ndc80	0.34112883	8.08E-105
2	Lockd	0.340489678	6.71E-62
2	Cenpi	0.340486925	5.41E-98
2	Ncapd2	0.340054707	2.36E-90
2	Nxt1	0.339467485	1.86E-22
2	Cox7a2	0.339042132	4.04E-09
2	H1f0	0.338571498	1.67E-07
2	Tubb2a	0.338535558	1.99E-06
2	Banf1	0.338103863	1.84E-10
2	Ucp2	0.335948902	6.27E-11
2	Lsm4	0.335859364	8.30E-10
2	Stub1	0.332095101	1.07E-09
2	Cct7	0.331666733	1.27E-07
2	Srsf7	0.330997083	5.86E-09
2	Eef1d	0.328724927	3.81E-05
2	Rpa1	0.32799024	2.77E-30
2	Hnrnpa3	0.327913797	3.07E-08
2	Pole3	0.32751679	2.33E-13
2	Hirip3	0.324607991	2.79E-43
2	Rad18	0.324506025	6.11E-45
2	Bcat1	0.324318382	8.55E-11
2	Tuba1a	0.324051414	1.49E-07
2	Snu13	0.323540436	1.10E-07

3	Spp1	1.908868403	5.91E-43
3	Timp1	1.489270578	4.73E-36
3	Nupr1	1.407135802	2.09E-34
3	Flt1	1.360207292	1.59E-74
3	Car4	1.295349809	8.19E-31
3	Sdc1	1.280811571	1.53E-40
3	Cd53	1.256513769	9.22E-75
3	Srgn	1.187586145	6.30E-22
3	Lgals3	1.051014552	7.33E-35
3	Ndufa4l2	1.04681359	4.45E-27
3	Igfbp6	1.020683527	1.51E-26
3	Itga1	0.978975285	6.52E-30
3	Calcrl	0.927253856	8.59E-43
3	Csf1	0.886655357	2.47E-20
3	Il13ra2	0.885685153	2.51E-71
3	Timp3	0.870568182	9.81E-15
3	Fn1	0.857513793	2.80E-28
3	Serpina3g	0.831952637	4.18E-23
3	Atf5	0.824136097	2.36E-16
3	Des	0.791833383	4.81E-18
3	Col6a5	0.79079377	1.37E-05
3	Cxcl16	0.759712536	1.08E-14
3	Olfr810	0.759050377	4.20E-98
3	Dio3	0.741174682	5.76E-20
3	Cd81	0.735296693	1.63E-30
3	Ccl11	0.718912174	4.51E-09
3	Ncam1	0.7188485	5.91E-22
3	Eif4ebp1	0.715700908	2.46E-14
3	Lox	0.685806133	4.13E-15
3	Serpinf1	0.681910093	5.94E-12
3	Capg	0.681232611	1.17E-13
3	Timp2	0.66836847	1.17E-24
3	Fabp5	0.665574638	5.66E-08
3	Cryaa	0.660745412	2.06E-63
3	Pla2g15	0.648861127	1.16E-17
3	Arg1	0.647519224	7.88E-12
3	Cyp7b1	0.640171666	1.26E-11
3	Gas5	0.63693912	2.19E-06
3	Sh3bgrl3	0.635418576	4.72E-16
3	Epas1	0.630823281	1.67E-16
3	Edil3	0.630062288	1.23E-67
3	Ecscr	0.619375337	2.75E-11
3	Clic1	0.593944431	3.21E-12
3	Sulf1	0.588112847	1.21E-11
3	Rasl11a	0.584017249	7.76E-15
3	Fndc3a	0.583154024	6.20E-11
3	Npc2	0.575567324	3.29E-24
3	Ctsk	0.561035797	2.75E-05
3	Rgcc	0.557307362	0.0063192
3	Cyba	0.548859764	3.87E-11
3	Tnfrsf9	0.536894394	1.51E-19
3	Asns	0.535455029	2.83E-06

3	S100a6	0.533373714	1.49E-20
3	Il4ra	0.530500633	1.02E-09
3	Crip2	0.528032884	9.56E-12
3	Il2rg	0.525941167	7.09E-41
3	Fxyd5	0.524848256	1.26E-08
3	Tpi1	0.518316541	7.56E-16
3	Upp1	0.516712903	2.71E-62
3	Cd151	0.503210359	5.68E-09
3	Hivep3	0.500051797	3.37E-18
3	S100a1	0.497869935	3.61E-10
3	Pmp22	0.494958822	7.08E-09
3	Rab15	0.490393739	3.56E-33
3	Ltbp2	0.490125387	7.26E-09
3	Brinp3	0.487871879	1.36E-22
3	B4galt5	0.486393643	1.31E-07
3	Angptl8	0.483961176	8.29E-23
3	Mthfd2	0.480795593	8.31E-05
3	Rabac1	0.47711849	1.28E-11
3	Tspo	0.475114827	8.70E-13
3	Wt1	0.474201916	1.40E-20
3	RP23-121N17.2	0.472907314	2.25E-60
3	RP24-175N4.1	0.468855372	1.93E-16
3	Plxdc2	0.467325755	1.53E-09
3	Emp3	0.464094815	2.43E-05
3	Aldoa	0.460018889	2.43E-08
3	Mt1	0.45793411	0.0007529
3	Pde4b	0.448806595	4.14E-06
3	RP23-234K24.8	0.444246583	0.000374
3	Tspan4	0.443748188	6.87E-06
3	Casp6	0.436244768	2.49E-10
3	Eno1	0.433832479	8.52E-06
3	Ctsc	0.422374227	2.39E-10
3	Ctsl	0.421758145	1.01E-14
3	Pkm	0.419774013	5.95E-11
3	Serpina3n	0.419193711	5.51E-05
3	Hdac9	0.418937969	7.47E-23
3	Mxra8	0.417955983	2.24E-06
3	Jun	0.414752684	2.78E-07
3	Hexa	0.412187568	1.27E-06
3	Ecm1	0.411677033	5.62E-06
3	Aif1l	0.411049007	7.67E-13
3	Gatm	0.409728988	1.24E-34
3	Rpl23	0.406189053	2.39E-13
3	Slc7a2	0.405241902	5.13E-07
3	Mif	0.40203207	2.27E-05
3	Cd63	0.401047568	3.92E-07
3	C1qtnf1	0.400453795	3.08E-11
3	Pcolce2	0.398323197	0.0002999
3	Fbln2	0.393069486	1.18E-12
3	Rassf1	0.390686312	0.0002652
3	Nptn	0.38867855	6.21E-05
3	Fos	0.386277117	0.0005151

3	Ak2	0.385557177	0.0018333
3	Azin2	0.384549536	5.97E-09
3	Slc44a2	0.382560075	3.97E-08
3	Cdh6	0.381688041	3.04E-24
3	Ass1	0.381464684	0.0300897
3	Gapdh	0.375634791	0.0007421
3	Ero1l	0.371929945	0.0008941
3	Cd200	0.368977977	2.44E-06
3	Zcchc24	0.365819085	0.0001148
3	Uqcrq	0.361418954	8.58E-05
3	Chst1	0.358940486	9.95E-27
3	Slc7a5	0.356038379	1.78E-08
3	RP23-285C18.2	0.352185462	4.51E-08
3	Tnip1	0.351345115	6.34E-05
3	Pgls	0.350821045	0.001669
3	Bgn	0.349856953	5.12E-07
3	Slc16a3	0.348852075	0.0004894
3	Gm2a	0.343910685	0.0014548
3	H2afj	0.342688561	0.0001273
3	Cblb	0.342229974	0.0159883
3	Plec	0.34163686	0.0008303
3	Serpine2	0.340817858	0.0005771
3	Rpl32	0.338545191	1.28E-06
3	Eef2	0.335562372	5.43E-08
3	Abca1	0.33475472	0.0022016
3	Pgk1	0.332840798	0.0001379
3	Sdf2	0.32977305	0.0033812
3	Eef1a1	0.327889934	2.26E-09
3	Rpl14	0.327676089	1.29E-05
3	Aga	0.325476996	0.0053864
4	Crabp1	2.293140322	2.53E-57
4	Penk	1.750895798	2.14E-50
4	Reg3g	1.648284636	1.00E-23
4	Igfbp2	1.505429306	8.69E-47
4	Igf1	1.404890032	8.46E-28
4	Slit2	1.394365551	1.65E-23
4	Lum	1.299925721	2.24E-29
4	Cpz	1.284368402	3.35E-25
4	Pla1a	1.250083382	3.65E-26
4	Enpp2	1.224280165	8.25E-40
4	Rgs2	1.190753525	5.83E-06
4	Col18a1	1.149916101	1.18E-40
4	Sfrp2	1.14290653	3.64E-17
4	Hc	1.127635468	2.82E-63
4	Col4a2	1.118730539	1.64E-21
4	Hmcn1	1.104050011	6.17E-22
4	Spon1	1.033831992	2.02E-15
4	Masp1	1.007136132	5.87E-18
4	Lama2	1.001186054	5.32E-24
4	Col4a1	1.001007984	9.66E-16
4	Dcn	0.995537293	2.60E-21
4	Col15a1	0.968834146	4.76E-14

4	Col13a1	0.965517479	7.57E-124
4	Steap4	0.963776786	3.97E-21
4	Gas1	0.944733173	4.47E-11
4	Mafb	0.943525306	1.13E-13
4	Bmp7	0.938430398	1.02E-38
4	Mmp19	0.913173349	7.25E-11
4	Srpx	0.898661209	2.49E-12
4	Mmp2	0.878286853	7.60E-18
4	Il11ra1	0.870054162	1.75E-11
4	Fcgr2b	0.847155951	3.89E-38
4	Apod	0.822510588	1.85E-14
4	Ptpre	0.804295018	2.02E-37
4	Hsd11b1	0.80105091	4.05E-05
4	Serpine2	0.800669108	0.001623
4	Thy1	0.781015301	4.76E-15
4	Tcf4	0.777681529	4.75E-06
4	Col6a2	0.768877153	1.15E-13
4	Scube1	0.766220804	9.36E-53
4	Col6a1	0.763278086	1.11E-13
4	Svep1	0.757071042	1.91E-07
4	Kif1a	0.756902444	3.13E-34
4	Mfap2	0.756667946	6.20E-10
4	Cyp4b1	0.750214941	4.21E-11
4	Zfp36l2	0.747915421	4.87E-10
4	Cpxm1	0.746985199	1.74E-05
4	Ednra	0.74221385	1.12E-15
4	F2r	0.734558946	0.006068
4	Fbln7	0.734435105	1.34E-08
4	Frem1	0.72820415	6.23E-32
4	Postn	0.71790753	3.11E-05
4	Crispld1	0.716406824	1.40E-63
4	Fzd1	0.700966121	6.50E-08
4	Eln	0.699061543	4.74E-07
4	Ereg	0.695436683	7.73E-05
4	Plpp1	0.685156212	6.69E-06
4	Amn	0.665453479	7.13E-35
4	Nnmt	0.665165488	2.13E-07
4	Abi3bp	0.659709882	0.0001203
4	Pdlim2	0.656739157	0.0018844
4	Rarres1	0.653807989	1.21E-31
4	Bdnf	0.651665498	7.71E-23
4	Igsf10	0.643239465	4.51E-08
4	Egflam	0.642224223	2.70E-96
4	Sntb2	0.639048621	1.75E-08
4	Fbln5	0.638511853	2.26E-07
4	Sdc1	0.636300021	2.80E-08
4	Twist1	0.63367156	6.08E-05
4	Lhfpl2	0.631671687	0.0006432
4	Lpl	0.629464983	2.79E-06
4	Tmod3	0.623837817	0.0102168
4	Zbtb20	0.623731099	3.95E-08
4	Ramp2	0.621118179	1.07E-05

4	Ism1	0.606328573	3.48E-27
4	Entpd1	0.604227021	5.97E-16
4	Ace	0.603458487	0.0229554
4	Igfbp4	0.601115896	2.84E-08
4	Abca8a	0.596798432	4.96E-17
4	Tpst1	0.59348672	2.00E-08
4	Mir99ahg	0.593411413	1.32E-07
4	Sema5a	0.591881571	1.10E-07
4	Lhfp	0.584308798	4.39E-10
4	Pth1r	0.576201688	0.0006747
4	Adcyap1r1	0.573932051	5.22E-31
4	Tmem132c	0.566876184	2.73E-31
4	Cst3	0.56682105	1.40E-11
4	Auts2	0.565514956	9.81E-06
4	Sh3pxd2a	0.554538441	4.18E-05
4	Ppic	0.546943166	1.13E-05
4	Crlf1	0.542120556	0.0211961
4	Ifitm2	0.536713597	2.38E-09
4	Tmsb10	0.532604246	7.18E-05
4	Cpq	0.532413798	9.61E-05
4	Gria3	0.53070308	1.09E-11
4	Lrig1	0.52065697	7.71E-07
4	Creg1	0.517476928	0.0182971
4	Pde5a	0.517198874	0.0001258
4	Mgat5	0.515284334	0.0070068
4	Podxl2	0.514922226	3.21E-05
4	RP23-459L15.8	0.511713048	0.0214473
4	Celf2	0.510194168	0.0115895
4	Sbspon	0.506620063	1.85E-18
4	Gpm6b	0.506194539	0.0005823
4	Apoe	0.502992091	0.0013822
4	Fkbp9	0.499071182	0.0002925
4	Ociad2	0.494433598	0.0034959
4	Angptl4	0.488469197	0.025518
4	Tgfbr3	0.487885714	0.002466
4	Rnase4	0.48407405	8.07E-07
4	Zmiz1	0.478495559	0.0007672
4	Sesn3	0.478473314	2.46E-07
4	Hspg2	0.475020385	0.0002341
4	Cd302	0.470244474	0.0003901
4	Slc9a3r2	0.46813681	1.74E-11
4	Cotl1	0.466482618	0.0060734
4	Efna5	0.46611794	1.81E-05
4	Adamts2	0.462743137	0.0023211
4	Pld3	0.462712579	0.0006555
4	Cd9	0.460408603	1.61E-05
4	C1qtnf2	0.459901879	1.12E-07
4	Ltbp1	0.455766489	0.0375307
4	S1pr1	0.455311451	1.24E-08
4	Shc4	0.45450591	0.0037167
4	Gpt2	0.44758261	1.67E-11
4	Sh3bgrl3	0.44636041	0.0488474

4	Lpin1	0.445652629	0.0012667
4	Tpbg	0.443231478	0.0003069
4	Ccnd2	0.44187951	0.003219
4	Adam23	0.438641309	4.26E-11
4	Il1rap	0.435171026	0.0488782
4	Nr2f2	0.432980744	0.0096965
4	Aldh7a1	0.428717009	0.0068106
4	Loxl1	0.42846326	0.0003388
4	RP23-195A18.3	0.427265054	7.80E-18
4	Matn2	0.424871127	0.0024875
4	Cdkn2b	0.42387348	0.0020094
4	Plscr4	0.4237924	6.62E-07
4	Fuca1	0.414796963	0.0008503
4	C1ra	0.412463147	0.0044901
4	Htra1	0.410588439	0.0460715
4	Arhgef7	0.404887788	0.0069103
4	Rgs10	0.400653767	0.0019372
4	Mark1	0.398113384	0.0378953
4	Bace2	0.396399816	1.26E-08
4	Abcc5	0.39078619	0.0001596
4	Plpp3	0.389147032	0.0034903
4	Phactr1	0.387897334	6.68E-06
4	Pard3b	0.38541878	0.0026812
4	C1qtnf6	0.384334581	0.0158324
4	Dlg2	0.374913948	2.98E-24
4	Steap3	0.374835776	0.0189887
4	Sfrp1	0.372104028	0.0115971
4	Malat1	0.368779271	6.70E-05
4	Cd81	0.36816684	0.0277317
4	Fam132a	0.367127634	0.0028996
4	Col3a1	0.364387947	7.64E-06
4	RP24-511A23.2	0.363973272	1.79E-05
4	Kdm7a	0.362939	0.0109818
4	Ndnf	0.362006805	4.73E-06
4	Mgp	0.361298425	2.67E-09
4	Fth1.1	0.360257674	1.96E-05
4	Ephb2	0.359223113	1.90E-05
4	Pcdh18	0.358720826	0.0051967
4	Kitl	0.352076318	0.0329346
4	Gcnt4	0.350174287	1.59E-13
4	Cd34	0.349445084	0.0019614
4	Itga2b	0.346985724	4.51E-10
4	Tril	0.340268115	2.36E-06
4	Xist	0.327250218	0.0366521
5	Krt18	2.541657331	1.28E-19
5	Wfdc2	2.468196471	7.14E-18
5	Krt8	2.208654757	7.36E-23
5	Cd74	2.093274113	2.22E-15
5	Ccnd1	1.794934763	1.26E-18
5	H2-Eb1	1.725575035	1.16E-22
5	Epcam	1.635838554	2.26E-28
5	Lcn2	1.48539433	1.59E-07

5	H2-Aa	1.429205079	1.16E-19
5	Krt7	1.324507447	9.31E-25
5	Cldn4	1.19996318	4.80E-56
5	Fgfbp1	1.140791148	3.67E-49
5	Ndufb9	1.118100427	8.63E-07
5	Sfn	1.057609068	4.86E-50
5	Gm47283	1.052133427	3.12E-12
5	Tnnt2	1.039559485	3.33E-37
5	H2-Ab1	1.028453205	4.06E-08
5	Prkg2	1.026431421	1.85E-15
5	Cldn7	1.026151882	1.70E-53
5	Il23a	0.999075012	1.09E-58
5	Mtap	0.990754963	6.53E-08
5	Clu	0.977037984	1.49E-21
5	Spint2	0.963531934	1.68E-40
5	Dut	0.93683038	9.29E-08
5	Hmgb2	0.933139663	0.0234554
5	Itga3	0.931615545	1.64E-88
5	Top2a	0.931462724	9.83E-05
5	Neurl3	0.921703174	1.67E-32
5	Trps1	0.908211979	6.37E-19
5	Mmp9	0.907202371	8.37E-05
5	RP23-465M17.1	0.891953941	2.14E-40
5	RP23-52N2.1	0.889526283	4.81E-09
5	Polr2f	0.879997799	2.56E-05
5	Cpe	0.878484953	1.34E-43
5	Psme1	0.874894963	4.42E-06
5	Cldn3	0.859485041	1.97E-49
5	RP23-71J17.1	0.853518372	2.84E-09
5	H2-DMa	0.83429913	2.89E-36
5	S100a4	0.828525426	0.0007085
5	Aprt	0.821003138	0.0008799
5	Sox4	0.812192438	0.0084491
5	Alcam	0.807502827	8.91E-58
5	Csf3	0.788445751	4.13E-11
5	Npm1	0.765520312	0.0001965
5	Psmb9	0.76535477	0.000316
5	Casp3	0.758644849	2.19E-08
5	RP23-442M18.1	0.74402415	3.99E-22
5	Kcnn4	0.741043948	1.64E-68
5	Tmprss11e	0.740446428	9.95E-128
5	Krt14	0.729854913	1.85E-23
5	Hmga2	0.725116465	4.85E-51
5	RP23-310J6.1	0.719892361	0.0019245
5	Ramp1	0.709863437	5.52E-22
5	Nav2	0.705352967	1.64E-72
5	Etv1	0.703786583	9.54E-26
5	Mtss1	0.699433078	3.55E-32
5	RP23-234K24.8	0.698096345	0.0044344
5	Sdc4	0.69395822	0.0115794
5	Ppia	0.688116308	0.015046
5	Psma6	0.686078185	0.0081344

5	Ccbe1	0.685540574	0.0003729
5	Itga6	0.684755054	2.70E-47
5	Cgnl1	0.679450786	4.52E-14
5	Ezr	0.678397305	3.96E-10
5	Cxadr	0.678293398	1.00E-73
5	H2-DMb1	0.672364	1.88E-41
5	Rp9	0.667849858	0.0147562
5	Sesn3	0.666318267	6.16E-08
5	Hoxa5	0.661030918	2.80E-43
5	Ptma	0.655676335	0.0027989
5	Krtcap3	0.652018076	4.12E-91
5	Cks1b	0.64552079	0.0004128
5	Eno3	0.643322865	3.19E-19
5	Tns4	0.640558918	1.78E-81
5	Sox9	0.626224339	9.02E-08
5	Cd24a	0.625199703	8.45E-42
5	Amotl1	0.62407593	0.0243621
5	Npnt	0.621966917	3.89E-55
5	Ngfrap1	0.621455624	0.0001166
5	Gas5	0.615800451	0.042156
5	Fermt1	0.614104239	1.14E-136
5	Atad2	0.613187424	0.0006335
5	Snhg1	0.606736789	0.0001927
5	Slpi	0.606734509	9.67E-10
5	Tra2a	0.603760808	0.018773
5	Smc3	0.603523906	0.0008947
5	Gm26825	0.5927952	0.0022285
5	Luc7l3	0.591110024	0.015131
5	Fam49b	0.590328936	0.0003078
5	Hnrnpa1	0.58936279	0.0444092
5	Psip1	0.58568319	0.0112725
5	Traf1	0.585456369	2.28E-19
5	Slco2a1	0.581092803	3.52E-10
5	Dusp6	0.571947183	1.87E-07
5	Gsto1	0.568030125	0.0001013
5	Slc12a2	0.567767842	1.61E-07
5	Ptprk	0.565622397	2.88E-12
5	Sssca1	0.564493638	0.0002433
5	Gpa33	0.558543765	7.60E-68
5	RP23-214L4.1	0.552860909	8.41E-05
5	H3f3b	0.549348083	0.0474996
5	Egln3	0.539946607	4.86E-15
5	Hells	0.53687448	2.67E-06
5	Mtmr2	0.535543197	0.0083115
5	Stmn1	0.529673924	0.001167
5	Lamc2	0.529238565	1.04E-10
5	Vars	0.528782017	0.0001588
5	Fnbp1l	0.528572132	8.83E-09
5	Eif4a2	0.524089329	0.04301
5	Gata3	0.522513456	6.33E-18
5	Slc4a11	0.522143494	1.96E-14
5	Perp	0.516104185	7.21E-52

5	Insl6	0.515025232	2.62E-34
5	Rassf9	0.511848484	5.53E-50
5	Gsap	0.502398822	0.0182949
5	Tmem238	0.500291618	1.72E-14
5	Tmprss6	0.493732011	5.33E-32
5	H2-DMb2	0.492693649	2.07E-44
5	Acap1	0.492059083	7.41E-82
5	Slc15a3	0.489341216	1.05E-09
5	Ank	0.486764601	0.0040198
5	Ift172	0.485231798	3.15E-14
5	Padi4	0.483932269	1.76E-84
5	Med24	0.47430876	6.76E-07
5	Lama5	0.471293644	2.15E-30
5	Il24	0.470754897	8.22E-57
5	Igf2bp2	0.470053678	1.15E-05
5	Elovl6	0.468090382	7.80E-18
5	Ets2	0.467316817	0.0072547
5	Gcat	0.466119359	1.26E-09
5	Flrt3	0.463800957	1.18E-11
5	Stard10	0.462411828	3.39E-13
5	Rfc3	0.462157386	2.08E-13
5	Cenpv	0.461100843	9.40E-22
5	Cdkn2aipnl	0.458856574	2.36E-13
5	Bcl2	0.458746092	3.13E-07
5	Clcn3	0.457185068	6.78E-11
5	Ibsp	0.454806976	3.27E-21
5	Pnn	0.453135719	0.0485581
5	Acsl3	0.451514632	0.0013967
5	Tk1	0.450992875	1.36E-05
5	Itpr3	0.450190164	1.20E-13
5	Adgrl2	0.450169155	2.04E-06
5	Ankrd1	0.449092997	0.0004909
5	Trmt1	0.448662451	4.81E-06
5	Phgdh	0.447796022	1.24E-15
5	Ehd4	0.442860592	1.53E-08
5	Ppp1r14c	0.436099707	2.17E-43
5	Pick1	0.434941778	1.69E-10
5	Fah	0.430260976	0.0002578
5	Tinagl1	0.429444343	4.14E-33
5	Zfas1	0.428651591	0.0083859
5	Tmem128	0.427963252	0.0002007
5	Urah	0.4235314	5.12E-47
5	Glrp1	0.423214983	5.40E-47
5	Slc29a1	0.423031393	4.18E-05
5	Cyld	0.422626154	0.006443
5	Mcm6	0.421056892	0.0017746
5	Fut8	0.420361125	0.0020945
5	Plcg2	0.420046292	6.60E-45
5	Cdh1	0.420002961	2.14E-39
5	Ilf3	0.415998097	0.0045228
5	Ncoa6	0.409229006	0.0017596
5	Gbp4	0.407415185	9.00E-07

5	Nlrc5	0.407389086	2.96E-07
5	Tmem132a	0.40721149	7.70E-06
5	St14	0.406814573	2.01E-77
5	Pdgfb	0.40661349	2.66E-55
5	H2-Ea-ps	0.404520598	6.04E-49
5	Irx2	0.404383923	1.07E-10
5	2010300F17Rik	0.403213105	4.42E-28
5	AC162528.2	0.40281106	0.001375
5	Sms	0.402579181	2.66E-12
5	Trim33	0.398989835	0.0028827
5	Kank3	0.398002706	8.89E-33
5	Wdr36	0.396750596	0.0014329
5	Cd82	0.395457693	0.0168021
5	Tcof1	0.395042396	0.0015751
5	Scx	0.394585433	1.02E-32
5	Card10	0.393507167	1.20E-53
5	Lig1	0.393441462	1.22E-07
5	Cp	0.392168843	0.0028327
5	Itgb4	0.392141077	2.72E-39
5	Prss22	0.392131394	6.48E-34
5	Arhgef5	0.391518469	2.33E-05
5	Prps1	0.391259624	0.0003896
5	Epb41	0.388947885	3.12E-16
5	Fam76b	0.387942389	3.44E-05
5	Tcf7l2	0.38596649	0.0081233
5	Dctpp1	0.38505823	0.0399192
5	Lad1	0.38413312	1.17E-44
5	Phlda2	0.380443523	8.06E-51
5	Prrc2b	0.379565334	0.0159374
5	Usp15	0.378946095	0.0019059
5	Wnt10a	0.378560414	2.28E-21
5	Arg1	0.374575703	0.0251348
5	Rap2c	0.373886928	0.0149885
5	Slc43a2	0.373708164	4.70E-20
5	Msln	0.372455142	3.63E-55
5	Ptpre	0.36823732	3.59E-11
5	Hmgb3	0.368166178	1.64E-15
5	Ppp1r12b	0.366656088	0.0048998
5	Wnt7b	0.366413529	3.10E-73
5	Mta1	0.364743203	0.0305713
5	Esrp1	0.363375174	1.13E-72
5	BC003965	0.362862593	0.0261309
5	Lsr	0.362631422	1.98E-43
5	Rnpep	0.362523655	2.03E-05
5	Wee1	0.360121939	1.92E-06
5	Dkc1	0.359895448	0.0084373
5	Nipsnap1	0.357777118	6.92E-42
5	Tia1	0.354261076	0.0357222
5	Dnajc2	0.354096904	0.0158102
5	Zc2hc1a	0.353923679	0.0123782
5	Dtl	0.35374156	2.83E-05
5	Las1l	0.353580468	0.0062374

5	Endod1	0.35125629	0.0035997
5	Hook1	0.351182521	2.07E-59
5	Txnrd2	0.350428877	0.0002586
5	Il18rap	0.350381705	1.18E-16
5	Trim59	0.347905142	1.89E-07
5	Pop5	0.347852526	0.0257022
5	Capn5	0.34700343	4.82E-07
5	Eva1c	0.346074763	5.45E-68
5	Smpdl3b	0.344942897	7.47E-17
5	Med19	0.343479585	0.0013442
5	Pcna	0.341308601	0.0074367
5	RP23-101F14.3	0.339091897	0.0172935
5	Cx3cl1	0.337861495	0.0278126
5	Cdcp1	0.337482191	1.22E-69
5	Phka2	0.336602642	0.0037377
5	Trap1	0.335722584	0.0005386
5	Rpa2	0.335661164	0.0038206
5	Ppat	0.334795214	0.0001781
5	AC164564.3	0.33411637	1.60E-05
5	Ptpn18	0.332509571	2.02E-16
5	Mrps5	0.330418582	0.0012793
5	Gmnn	0.330326507	6.93E-07
5	Hspa4l	0.32984808	0.0008613
5	Rabepk	0.328551726	0.0024525
5	Emp2	0.328389975	0.0419896
5	Chaf1b	0.327749354	4.69E-08
5	Spint1	0.326853206	1.57E-68
5	Plet1	0.326814219	3.55E-11
5	Usp10	0.325348366	0.0017764
5	Gjb3	0.324927622	1.46E-14
5	Echdc3	0.324218992	3.01E-21
5	Etv4	0.323977639	0.0204293
5	Nt5e	0.323602314	5.61E-07

Table S3. Genes enriched (fold change >1.25) in each normal mammary fibroblast cluster compared to all other clusters.

Cluster ID	*Marker genes*	*Fold enrichment (log2)*	*p_val_adj*
0	Mgp	1.705374041	5.02E-60
0	Cxcl1	1.020577968	6.40E-85
0	Fmo2	0.965584584	1.89E-59
0	Gstm1	0.944751011	7.20E-111
0	Ccl7	0.931839485	4.49E-54
0	Sparcl1	0.900902479	1.07E-99
0	Rbp1	0.867038719	1.65E-92
0	Lpl	0.844519554	2.80E-59
0	Gpx3	0.810399653	2.95E-77
0	S100a1	0.803408734	9.32E-101
0	Ccl2	0.801805164	1.68E-38
0	Bgn	0.783597333	7.79E-67
0	Fth1.1	0.780786883	7.88E-110
0	Hpgd	0.756091178	3.48E-71
0	Itm2b	0.746960864	2.49E-100
0	Igfbp7	0.736648284	1.86E-66
0	Pnp	0.729766962	4.87E-72
0	Cst3	0.721585102	2.62E-79
0	Tpt1	0.719187075	4.08E-91
0	Dcn	0.716458288	3.04E-103
0	Dpep1	0.711386513	1.73E-60
0	Cyb5a	0.710134423	4.69E-84
0	Cd81	0.699058035	7.71E-89
0	Sepp1	0.696202845	1.90E-89
0	Rps4x	0.693775673	4.42E-75
0	Maff	0.692271707	4.07E-59
0	Smoc2	0.687119232	3.15E-54
0	Ogn	0.680014694	3.53E-73
0	Ccl11	0.677082195	4.69E-28
0	Dbi	0.663287326	2.08E-61
0	Rplp1	0.663138171	1.25E-79
0	Cxcl14	0.661590088	2.03E-11
0	Ech1	0.659808235	9.28E-71
0	Mt1	0.659232992	9.72E-54
0	Rpl32	0.650143262	6.16E-71
0	Rps14	0.643636816	6.93E-71
0	Rps5	0.641977973	1.06E-70
0	S100a6	0.637108696	3.42E-85
0	S100a10	0.634975332	7.78E-73
0	Rpl39	0.632627822	5.01E-67
0	Rps3	0.627805936	1.38E-71
0	Eef1a1	0.619481023	8.87E-82
0	Acaa2	0.618930651	1.18E-61
0	Ifitm2	0.618145365	3.81E-67
0	Cygb	0.61464255	9.20E-54
0	Rps24	0.614028073	2.44E-63
0	Cfh	0.612983409	4.76E-33
0	S100a11	0.61262632	2.13E-68
0	Rpl34	0.612326832	2.53E-66
0	Ftl1	0.609495646	5.30E-68
0	Gas1	0.608282823	7.21E-29
0	Rpl23	0.605788619	1.29E-65
0	Rps11	0.60527904	5.26E-69
0	Emp3	0.603011175	6.66E-63
0	Ifitm3	0.597904433	5.57E-59
0	Cox7a2l	0.597150579	7.91E-69
0	Rps9	0.596768713	4.34E-66
0	Serping1	0.595978781	1.16E-72

0	Rps15a	0.593211235	4.39E-66
0	Prdx5	0.593207683	2.77E-60
0	Ubb	0.592464633	3.78E-60
0	Adh1	0.590244699	9.63E-57
0	Snhg18	0.58558029	1.22E-69
0	Fxyd5	0.585096892	4.56E-78
0	Apoe	0.583118795	1.68E-17
0	Tmsb10	0.58036375	3.57E-56
0	Rpl22	0.579695236	4.39E-58
0	Gng5	0.577249719	5.48E-61
0	Rpl8	0.575660757	1.60E-69
0	Ctsl	0.574314619	4.71E-77
0	Sqstm1	0.571987315	1.67E-42
0	Rplp0	0.569885408	1.57E-60
0	Rpl14	0.568894918	7.31E-60
0	Gstt1	0.565245964	1.41E-69
0	Fbln1	0.564819588	9.84E-42
0	Selm	0.561614382	2.19E-56
0	Ctsh	0.55973032	3.62E-55
0	Cd302	0.556949732	2.32E-57
0	Ppib	0.554781048	1.67E-62
0	Gpx8	0.55096127	1.13E-63
0	Rps21	0.550114545	2.99E-65
0	Actb	0.548553294	8.02E-57
0	Prdx1	0.547828041	2.89E-53
0	Rabac1	0.545541866	2.03E-60
0	Maged2	0.545121375	8.25E-49
0	Uqcrh	0.544218905	1.96E-54
0	Plau	0.542801097	3.13E-44
0	F3	0.537943905	1.20E-37
0	Cxcl16	0.537027427	8.06E-53
0	Ppia	0.535738971	1.20E-61
0	Phlda1	0.533267369	4.06E-35
0	Gnb2l1	0.532056022	2.94E-58
0	Mmp23	0.531514102	2.32E-59
0	Ndufa4	0.530974199	6.70E-57
0	Nop58	0.530457184	2.27E-35
0	Lum	0.529103246	5.08E-47
0	Cox8a	0.528389444	6.19E-55
0	Tuba1a	0.526004972	3.73E-34
0	RP23-79J21.3	0.52589353	6.91E-52
0	Rpl37	0.525820254	2.30E-53
0	Dnajb1	0.524326921	6.29E-30
0	Tmsb4x	0.522087703	1.04E-56
0	Tubb4b	0.520318967	3.46E-36
0	Dstn	0.518523216	4.17E-48
0	Thbd	0.51812443	3.42E-33
0	Rps3a1	0.517655993	3.18E-49
0	Rpl4	0.515868866	4.34E-56
0	Rps26	0.514040281	1.88E-46
0	AC026478.1	0.509875469	4.06E-50
0	Rpl41	0.509553499	5.97E-51
0	Npc2	0.50947967	6.45E-55
0	Txn1	0.50802935	1.01E-47
0	Cstb	0.506645044	8.29E-42
0	Pfdn5	0.501169439	3.01E-50
0	Cxcl2	0.500277212	0.00048007
0	Eif1	0.498925801	1.50E-58
0	Gabarap	0.498922572	7.46E-47
0	Myl12a	0.494167887	8.61E-57
0	Pdia3	0.49223974	9.39E-51

0	Cebpd	0.489317633	6.02E-31
0	Rpl35	0.489183492	2.82E-45
0	Tspo	0.489031578	2.78E-48
0	Igfbp4	0.48834381	2.75E-46
0	Rplp2	0.486639154	1.53E-50
0	Park7	0.486409749	1.48E-58
0	Gnai2	0.486401319	3.28E-51
0	Aldh2	0.484669756	1.61E-52
0	Drap1	0.484366328	3.37E-63
0	Arl13b	0.483929085	9.52E-36
0	Hmox1	0.482679898	6.50E-23
0	Atp5e	0.4817292	2.31E-45
0	Clic1	0.480805967	2.31E-44
0	Jund	0.479921417	3.80E-46
0	S100a13	0.479678517	1.60E-48
0	Rps15	0.478968871	2.55E-49
0	Rps10	0.478445051	1.54E-43
0	Vim	0.476804398	1.47E-59
0	15-Sep	0.476710862	2.65E-51
0	Figf	0.476556153	6.72E-50
0	Cox7a2	0.47524667	2.70E-47
0	Dpm3	0.474757291	4.12E-51
0	Mfap4	0.474054308	1.01E-12
0	Slc25a4	0.473155927	1.09E-46
0	Actg1	0.471611007	1.43E-40
0	Clta	0.467905669	1.76E-48
0	Sdpr	0.467862005	1.47E-51
0	Uqcr11	0.466424556	8.96E-52
0	Selk	0.46542694	1.96E-42
0	Cebpb	0.46496045	5.56E-36
0	Fxyd1	0.463249235	1.36E-52
0	Cryab	0.463186933	3.24E-19
0	Anxa5	0.45970448	2.78E-48
0	Eif3h	0.459704239	7.96E-51
0	Btf3	0.457492946	1.76E-43
0	Aldoa	0.45642906	3.92E-46
0	Cox4i1	0.455951352	5.24E-43
0	Junb	0.455819586	6.49E-34
0	Atp5j	0.455654005	5.75E-47
0	Nedd4	0.454696582	2.39E-56
0	Naca	0.454235485	3.95E-41
0	Colec12	0.452877603	6.20E-52
0	Hint1	0.451972456	7.81E-46
0	Sparc	0.451775097	8.05E-46
0	H3f3b	0.450536677	8.48E-54
0	Rps29	0.448316216	5.43E-43
0	Gstm2	0.448299503	6.23E-50
0	Ms4a4d	0.447054081	1.68E-53
0	Tagln2	0.446753849	1.30E-37
0	Anxa1	0.446614324	3.37E-37
0	Sod2	0.445783805	4.93E-31
0	Eif3k	0.445630137	4.84E-46
0	Eloc	0.444910754	8.27E-46
0	Cox7b	0.444572262	6.68E-47
0	Krtcap2	0.444167068	1.91E-46
0	Rps20	0.442760299	4.83E-36
0	Eif3f	0.442140653	5.66E-43
0	Atp5h	0.441073539	2.82E-52
0	Eef1d	0.440413359	1.36E-44
0	H3f3a	0.440106993	3.92E-43
0	Gem	0.439618997	1.06E-31

0	Ctsz	0.439173397	2.26E-46
0	Tcf21	0.437130367	5.21E-59
0	Ndufa7	0.435019669	1.06E-46
0	Nenf	0.434910928	7.41E-38
0	Rpl37a	0.434740453	7.07E-37
0	Swi5	0.434058737	2.54E-46
0	Fbln5	0.432862252	7.55E-28
0	Vcam1	0.43279746	6.27E-48
0	Cd63	0.430056369	4.25E-44
0	Map1lc3a	0.428008831	7.41E-43
0	Rps8	0.427749403	1.30E-35
0	Atp6v1f	0.426334916	1.13E-44
0	Rpl18	0.425289765	6.86E-48
0	Rpl22l1	0.422102556	9.43E-39
0	Lsp1	0.422017821	3.35E-16
0	Tmed3	0.420717893	2.70E-41
0	Timp2	0.416278892	1.48E-35
0	Sec61b	0.4162696	3.08E-40
0	Eef1b2	0.415385092	1.56E-31
0	Myc	0.414587119	6.74E-30
0	Nfkbia	0.414130382	6.69E-36
0	Rnase4	0.413516013	9.34E-41
0	Rspo1	0.412875319	9.67E-55
0	Gpm6b	0.412179103	5.33E-39
0	H2afz	0.411814748	1.22E-32
0	Clec3b	0.41127358	6.58E-43
0	Bri3	0.409212633	7.82E-38
0	Tomm7	0.409103854	1.83E-43
0	Crip2	0.406246898	1.99E-49
0	Calm2	0.40619215	9.06E-33
0	Sh3bgrl	0.405692426	1.83E-53
0	Cyba	0.404934387	3.01E-44
0	Rexo2	0.404530952	3.71E-41
0	Calm1	0.404528248	1.38E-36
0	Cfl1	0.403863999	3.10E-35
0	Mpc2	0.40166899	1.81E-41
0	Myl6	0.400181001	5.74E-38
0	Dynll1	0.400152026	3.09E-36
0	Map1lc3b	0.399576579	2.35E-35
0	Smdt1	0.398886514	2.97E-38
0	Chchd2	0.398329587	7.31E-34
0	Rpl38	0.398224395	4.34E-30
0	Aes	0.39795807	1.47E-44
0	Psmb1	0.3979052	7.91E-43
0	Sumo1	0.397820588	4.02E-43
0	Penk	0.397738716	1.74E-35
0	Rps28	0.397221619	8.11E-39
0	Srp14	0.397062758	8.11E-44
0	Pltp	0.396229876	1.03E-47
0	Atp5c1	0.395830585	8.28E-41
0	Socs3	0.395468192	8.65E-21
0	RP23-52N2.1	0.393668012	3.47E-33
0	Ier5	0.393510707	1.09E-24
0	RP23-263B18.4	0.393126309	7.12E-41
0	Spon2	0.393024736	1.92E-34
0	Sumo2	0.392985342	9.98E-40
0	Tomm20	0.392575875	4.82E-35
0	Cox6b1	0.390654935	7.62E-36
0	Pcolce	0.389825477	1.12E-29
0	Lgals1	0.389770357	6.82E-45
0	Pfn1	0.388096862	6.31E-39

0	Fabp4	0.387915536	2.51E-13
0	Atp5d	0.385695579	5.27E-36
0	Bsg	0.38488648	5.06E-37
0	Hspb1	0.38379216	7.95E-20
0	Tm4sf1	0.382230558	1.07E-45
0	Pdlim2	0.381962146	8.50E-43
0	Ier3	0.381561342	3.83E-11
0	Psmb2	0.381546819	2.89E-44
0	Arpc3	0.381522607	5.43E-38
0	Mxra7	0.381044238	2.18E-40
0	Rnd1	0.380835804	1.19E-20
0	Dpt	0.380588079	1.04E-37
0	Oaz1	0.378975391	3.72E-34
0	Hcfc1r1	0.378795463	2.33E-41
0	Calr	0.378302369	1.59E-37
0	Mxra8	0.376927702	1.59E-42
0	Fosl1	0.375584898	3.97E-29
0	Ube2b	0.375380236	2.55E-33
0	Pdia6	0.375265566	8.44E-36
0	Serpinh1	0.374734421	1.66E-29
0	Rpl36	0.374405243	1.32E-37
0	Laptm4a	0.374248383	5.92E-31
0	Sdc2	0.374150651	1.48E-36
0	Ddah2	0.373446345	1.51E-35
0	Serpinf1	0.373418937	5.54E-27
0	Rpl19	0.373252356	1.08E-41
0	Edf1	0.371567042	2.35E-38
0	Sde2	0.371290097	2.24E-32
0	Tuba1c	0.371204259	3.22E-39
0	Sepw1	0.370929664	3.16E-30
0	Cnpy2	0.370222207	1.38E-41
0	Spcs1	0.370083268	2.91E-35
0	Tmem256	0.37000996	2.87E-36
0	Entpd2	0.369308099	1.29E-39
0	Ostc	0.368789811	3.33E-37
0	Gpx4	0.367717153	1.11E-42
0	Nnmt	0.366996383	4.01E-29
0	Tceal9	0.366645243	1.37E-33
0	Vkorc1	0.366639659	2.47E-39
0	Cox5b	0.365046192	4.13E-37
0	Polr1d	0.363488098	9.74E-39
0	Tmem59	0.363390692	3.40E-35
0	Rpl7	0.36311003	5.35E-39
0	Vtn	0.363022124	9.80E-26
0	B2m	0.362698873	7.74E-28
0	Ndufb5	0.362676104	2.42E-39
0	Naalad2	0.362445457	1.59E-52
0	Cdc42	0.360442877	4.18E-32
0	Atp5j2	0.359996156	3.99E-32
0	Nr4a3	0.359278826	5.30E-20
0	Slc43a3	0.359062322	9.64E-32
0	Nedd8	0.358198343	3.22E-38
0	Gnas	0.357733124	5.26E-33
0	Fau	0.356571106	4.37E-37
0	Etfb	0.35597259	7.38E-37
0	Ergic3	0.355810845	2.88E-40
0	Atp5f1	0.355600823	3.53E-36
0	Ubl5	0.3550648	9.79E-35
0	Rps19	0.354920888	1.24E-29
0	Ost4	0.354037614	1.02E-33
0	Steap3	0.353863255	2.98E-34

0	Kdelr1	0.35333525	3.99E-36
0	Ndufv3	0.353304716	2.86E-36
0	Txndc17	0.352807701	1.12E-38
0	Iscu	0.352405715	3.23E-38
0	Elob	0.351157505	1.34E-33
0	Hsbp1	0.35079161	7.61E-35
0	Lamp1	0.350545927	1.07E-31
0	Atp5b	0.350470487	1.59E-30
0	Lamtor2	0.349962443	1.24E-41
0	Arpc2	0.348124494	2.15E-28
0	Fundc2	0.34776309	8.28E-40
0	Ndufa13	0.346734778	3.67E-31
0	Klf4	0.346599327	8.73E-17
0	Tmed10	0.346530964	3.48E-33
0	Atp6v0e	0.346286253	6.89E-36
0	Ldha	0.345941987	2.59E-27
0	Fkbp2	0.345685001	5.46E-39
0	Tmem258	0.345222787	5.61E-33
0	Ssr4	0.345097748	5.11E-29
0	Tmem50a	0.344392923	5.38E-35
0	RP24-142P6.5	0.344106223	3.73E-33
0	Ywhaq	0.344099919	4.70E-34
0	Cpq	0.344024845	1.15E-33
0	Ptges	0.343074712	1.48E-32
0	Npm1	0.342896723	5.51E-28
0	Atpif1	0.342176988	1.16E-33
0	Mrps14	0.341912518	1.06E-38
0	Rpl13a	0.341610604	1.75E-35
0	Myl12b	0.339834205	2.35E-36
0	Rps27l	0.33918473	6.60E-31
0	Mpc1	0.339174206	1.33E-37
0	Ndufa6	0.338691505	4.00E-38
0	H2-D1	0.338622833	6.38E-21
0	H2afj	0.338259187	1.18E-31
0	Pmp22	0.338119235	8.00E-41
0	Copb2	0.336211489	1.69E-33
0	Id3	0.335567986	5.09E-26
0	Nfix	0.334960993	6.49E-27
0	Tppp3	0.334590934	4.97E-22
0	Esd	0.334294087	3.18E-36
0	Eif5a	0.334081402	2.84E-27
0	RP23-55L3.5	0.334031281	8.28E-37
0	Ndufb6	0.33396174	2.77E-36
0	Plin2	0.33378454	1.50E-25
0	Islr	0.332518749	3.78E-22
0	Atraid	0.332195904	4.45E-36
0	Fcgrt	0.33170614	8.17E-30
0	Gnb2	0.331228712	3.67E-30
0	Ndufb11	0.330688504	2.16E-32
0	Spon1	0.330644195	6.10E-31
0	Ufm1	0.330602406	1.60E-33
0	RP23-123N23.5	0.330006693	3.99E-30
0	Sdc4	0.328058742	2.41E-23
0	Serf2	0.327808731	4.83E-31
0	Rhoj	0.326206972	3.96E-23
0	Erp29	0.32554258	1.30E-30
0	Hsp90b1	0.325126959	2.59E-26
0	Cope	0.324659853	1.57E-33
0	Etfa	0.324522225	3.08E-41
0	AC110186.1	0.323355865	9.31E-33
1	RP23-8J15.3	1.431858143	6.91E-143

1	RP23-8J15.4	1.413077751	4.19E-146
1	RP23-8J15.2	1.358620289	5.17E-138
1	RP23-8J15.7	1.320496482	3.17E-128
1	RP23-81C12.1	1.297981601	5.77E-41
1	RP23-8J15.6	1.284943223	1.36E-118
1	Malat1	1.282545066	7.37E-146
1	RP23-407K8.2	1.269321638	4.17E-86
1	RP23-8J15.5	1.242179523	2.61E-80
1	RP23-8J15.1	1.223483345	4.02E-138
1	Xist	1.15846246	1.17E-119
1	RP24-458F14.3	1.127803049	3.41E-44
1	RP23-407K8.3	1.037638019	1.78E-66
1	Mir6236	1.023724701	1.64E-57
1	Tsix	0.967526535	3.79E-19
1	Meg3	0.963458919	1.06E-15
1	Lars2	0.960897283	8.09E-17
1	Nktr	0.889793613	3.67E-55
1	RP24-459E13.2	0.88448528	4.67E-11
1	Kcnq1ot1	0.877377168	5.76E-07
1	RP23-446O11.1	0.876473147	9.60E-22
1	Ccnl2	0.872850348	4.27E-13
1	Neat1	0.837200585	3.58E-29
1	Nfat5	0.831491437	8.11E-43
1	RP24-458F14.4	0.817635959	8.42E-13
1	Fus	0.810350258	1.29E-31
1	Col5a3	0.788475849	4.49E-17
1	Ascc3	0.780652278	7.44E-11
1	Hspa1a	0.750957357	0.00127444
1	Prpf4b	0.74920385	9.40E-07
1	Ankrd17	0.741884852	3.81E-12
1	Myadm	0.739892071	0.00019306
1	Son	0.732510985	9.26E-61
1	Tnxb	0.725423957	6.04E-10
1	Dst	0.715537307	1.54E-10
1	Srsf11	0.704864683	3.93E-22
1	Taf1d	0.700447615	1.79E-10
1	Auts2	0.686184099	0.04596991
1	Fubp1	0.683456675	0.00026208
1	Ranbp2	0.668167159	5.78E-10
1	Bclaf1	0.664906242	0.01170637
1	Nfkb1	0.660641081	4.80E-12
1	Luc7l2	0.652609112	4.39E-17
1	Map4k4	0.651234218	8.10E-06
1	Fgfr1	0.648661603	0.00240405
1	Ewsr1	0.622069217	0.03909986
1	Ccnl1	0.618129052	4.01E-31
1	Cdk12	0.605327382	1.01E-05
1	Tra2b	0.601951238	2.05E-17
1	Hnrnph1	0.601360299	6.27E-14
1	Nsd3	0.597466169	3.09E-06
1	Tsc22d2	0.596995601	2.02E-06
1	Jmjd1c	0.594703718	0.02512025
1	Slc38a2	0.573776937	0.01347769
1	Gls	0.556606327	1.10E-09
1	Ebf1	0.551247059	7.42E-13
1	Srsf2	0.537441051	0.0002435
1	Rock2	0.530897828	2.45E-10
1	Rbm39	0.528460086	4.51E-22
1	Akap13	0.526618006	0.00286064
1	Spag9	0.526258236	7.06E-16
1	Sfpq	0.504450208	9.91E-11

1	Wnk1	0.498675684	0.00532318
1	Ahnak	0.472952762	5.80E-07
1	Zbtb20	0.442210417	3.25E-12
1	RP23-81C12.3	0.433366701	2.89E-21
1	Hivep2	0.425416496	0.0250658
1	Prrc2c	0.371422788	0.00076931
1	Col1a1	0.365167335	0.00876317
1	Ube2d3	0.355418286	0.03670237
2	Igfbp5	1.539467488	1.06E-32
2	Fgl2	1.121610095	1.99E-36
2	Pi16	1.099644222	2.32E-14
2	Timp3	1.025598564	9.52E-45
2	Pcolce2	0.928607273	6.72E-37
2	Ly6c1	0.897151879	6.23E-29
2	Apod	0.861662176	5.02E-55
2	Itm2a	0.842575325	1.12E-52
2	Il1rl1	0.822069003	2.66E-14
2	Akr1c18	0.822042411	2.29E-46
2	Plac8	0.800931958	1.10E-19
2	Csrp2	0.794661582	1.57E-38
2	Timp1	0.7515394	7.81E-05
2	Anxa3	0.747426874	5.47E-19
2	Mfap5	0.74098564	1.26E-36
2	Ly6a	0.740698708	8.13E-23
2	Gas7	0.724553381	5.87E-24
2	Cd34	0.707031055	1.29E-30
2	Fndc1	0.7042264	2.45E-18
2	Dpp4	0.691817071	2.95E-23
2	Crispld1	0.689534064	2.01E-76
2	Igfbp6	0.644286452	4.00E-29
2	Ctsk	0.637038798	6.70E-26
2	Uap1	0.613615151	5.10E-16
2	Postn	0.603383121	1.19E-16
2	Fst	0.598885624	1.75E-14
2	Procr	0.584979751	1.65E-07
2	Basp1	0.570178934	2.57E-23
2	Sfrp2	0.565911525	1.23E-06
2	Scg3	0.564673609	1.44E-17
2	Crip1	0.56281021	6.80E-27
2	Plpp3	0.559230517	4.36E-19
2	Marcks	0.547204226	1.01E-12
2	Cd9	0.542727351	1.45E-18
2	Zfhx4	0.54209568	4.55E-67
2	Col14a1	0.535380903	1.13E-10
2	Rarres2	0.534927494	7.85E-29
2	Mgst1	0.534732224	7.98E-28
2	Mmp2	0.529526712	5.82E-30
2	Ptx3	0.528219755	1.08E-05
2	Gpc3	0.527464591	6.69E-25
2	Htra1	0.520475902	4.74E-15
2	Igf1	0.519413519	1.03E-09
2	Thbs2	0.515152961	8.64E-17
2	Serpinb6a	0.503053872	3.51E-25
2	Gpx1	0.501530461	8.82E-14
2	Slco3a1	0.497460107	3.98E-34
2	Fstl1	0.495652813	1.30E-25
2	Has1	0.493047413	1.55E-15
2	Wnt2	0.488702589	1.49E-35
2	Bst2	0.487266167	0.00042895
2	Il1r2	0.47510548	9.37E-51
2	Sema3c	0.474794157	1.49E-15

2	Lbp	0.465873662	5.02E-14
2	Ecm1	0.463293222	5.97E-07
2	Lgals1	0.460490441	1.36E-12
2	Id2	0.454138828	1.52E-11
2	Mmp14	0.451227959	7.40E-22
2	Srpx	0.450053061	6.66E-30
2	Aspn	0.449621255	1.72E-05
2	Mmp3	0.447870562	7.35E-21
2	C4b	0.447219195	7.62E-14
2	Qpct	0.439877257	3.71E-18
2	Emb	0.43946867	2.10E-15
2	Mfap2	0.439205501	3.82E-28
2	Fn1	0.438750101	2.06E-14
2	Ppic	0.437787848	7.73E-22
2	Lgals3	0.437282026	1.96E-11
2	Atf3	0.434488069	1.47E-09
2	Cmah	0.434390227	2.68E-35
2	Ly6e	0.428816869	4.59E-11
2	Rbm3	0.428353597	1.32E-17
2	Ptgs2	0.428141546	6.87E-06
2	Anxa2	0.426612589	5.46E-13
2	Thy1	0.419543264	6.40E-20
2	Ace	0.419110784	2.47E-13
2	Rnase4	0.418275535	5.14E-16
2	Ackr3	0.417393763	5.06E-07
2	Ppp1r14b	0.41640043	3.02E-19
2	Sdc1	0.414056468	5.70E-26
2	Calcrl	0.413092285	4.02E-20
2	Loxl1	0.412377018	2.58E-19
2	Eef1b2	0.411998725	1.67E-22
2	Ccl8	0.408733863	7.74E-13
2	Arl4d	0.407791896	3.70E-15
2	Rbp4	0.399826656	3.24E-12
2	Oaf	0.399242952	1.26E-10
2	Dpt	0.397951236	2.08E-14
2	Gnpnat1	0.397748828	8.32E-24
2	Prrx1	0.394047928	4.58E-11
2	Lrrc17	0.39278163	5.55E-21
2	Ckb	0.391403173	1.49E-14
2	Actg1	0.388602409	3.34E-14
2	S100a16	0.386668669	4.74E-17
2	Abi3bp	0.386034907	6.00E-07
2	Efhd1	0.385376431	2.94E-26
2	Abracl	0.385039622	1.13E-10
2	Tubb5	0.382954263	8.00E-13
2	RP23-453B4.1	0.379638219	1.42E-20
2	Osr2	0.378736789	3.30E-09
2	Tnfaip6	0.378098594	1.41E-10
2	Cnih1	0.377170311	1.29E-25
2	Ybx1	0.377102797	7.39E-18
2	Tmem158	0.37702478	1.13E-16
2	Antxr1	0.374999051	1.41E-16
2	Mt2	0.374631178	7.62E-06
2	Cox6a1	0.37420338	3.30E-17
2	Cox5a	0.374031239	3.30E-15
2	Gng12	0.373954482	2.54E-19
2	Pla1a	0.373328696	2.24E-06
2	Tmsb4x	0.368819493	1.34E-15
2	Col1a2	0.366499312	6.39E-12
2	Slco2a1	0.365762268	2.14E-17
2	Hbegf	0.36270731	4.64E-08

2	Lrrn4cl	0.362091565	5.74E-12
2	Ndrg1	0.361659329	5.64E-08
2	Ugp2	0.361098584	0.00137799
2	Sem1	0.360798503	5.62E-18
2	Prss23	0.360717828	8.52E-07
2	Metrnl	0.357414755	7.42E-09
2	Rpl41	0.356814267	2.19E-18
2	Reep5	0.356118269	3.94E-16
2	Ybx3	0.355997607	7.65E-14
2	Anxa1	0.35403055	9.08E-10
2	Chrdl1	0.349716113	1.29E-22
2	Atox1	0.348617013	3.60E-15
2	Prkcdbp	0.348125496	3.81E-11
2	Plat	0.347909874	3.65E-13
2	Scand1	0.346880875	7.98E-12
2	Twist1	0.346507453	6.12E-14
2	Slc25a5	0.346337212	4.54E-09
2	Ttyh2	0.344869333	4.50E-28
2	Uqcc2	0.34450145	2.50E-15
2	Emilin2	0.344082103	1.53E-11
2	Rpl32	0.34267318	9.82E-16
2	Cadm3	0.339406791	2.93E-12
2	Scara3	0.334849495	4.51E-16
2	Nupr1	0.332437619	2.10E-07
2	Myl6	0.331611984	1.35E-08
2	Gnas	0.331387613	7.52E-16
2	Rps24	0.329687524	7.48E-16
2	AC129337.1	0.328293123	4.14E-22
2	Bcap31	0.327571966	4.92E-12
2	Car8	0.327142805	8.30E-23
2	Reep3	0.326881958	1.84E-14
2	Fap	0.326275937	8.96E-15
2	Cd248	0.325623898	5.01E-08
2	Rpl38	0.3253701	2.42E-14
2	Serpinf1	0.325275611	5.30E-12
2	Mtpn	0.324142463	2.68E-17
2	Rps10	0.323706406	1.32E-14
2	Cox6c	0.323648939	4.75E-14
